# Supplementary material for: Dual AAV gene therapy using laminin-linking proteins ameliorates muscle and nerve defects in LAMA2-related muscular dystrophy
Source: Mol Ther. 2026 Feb 2;34(5):2818–39. doi: 10.1016/j.ymthe.2026.01.041 (PMC13154268; doi:10.1016/j.ymthe.2026.01.041)
Supplement: Supplementary file 1 [file mmc1.pdf]

YMTHE, Volume *34*

Supplemental Information

Dual AAV gene therapy using laminin-linking proteins ameliorates muscle and nerve defects in LAMA2-related muscular dystrophy

Judith R. Reinhard, Shuo Lin, Eleonora Maino, Daniel J. Ham, and Markus A. Rüegg

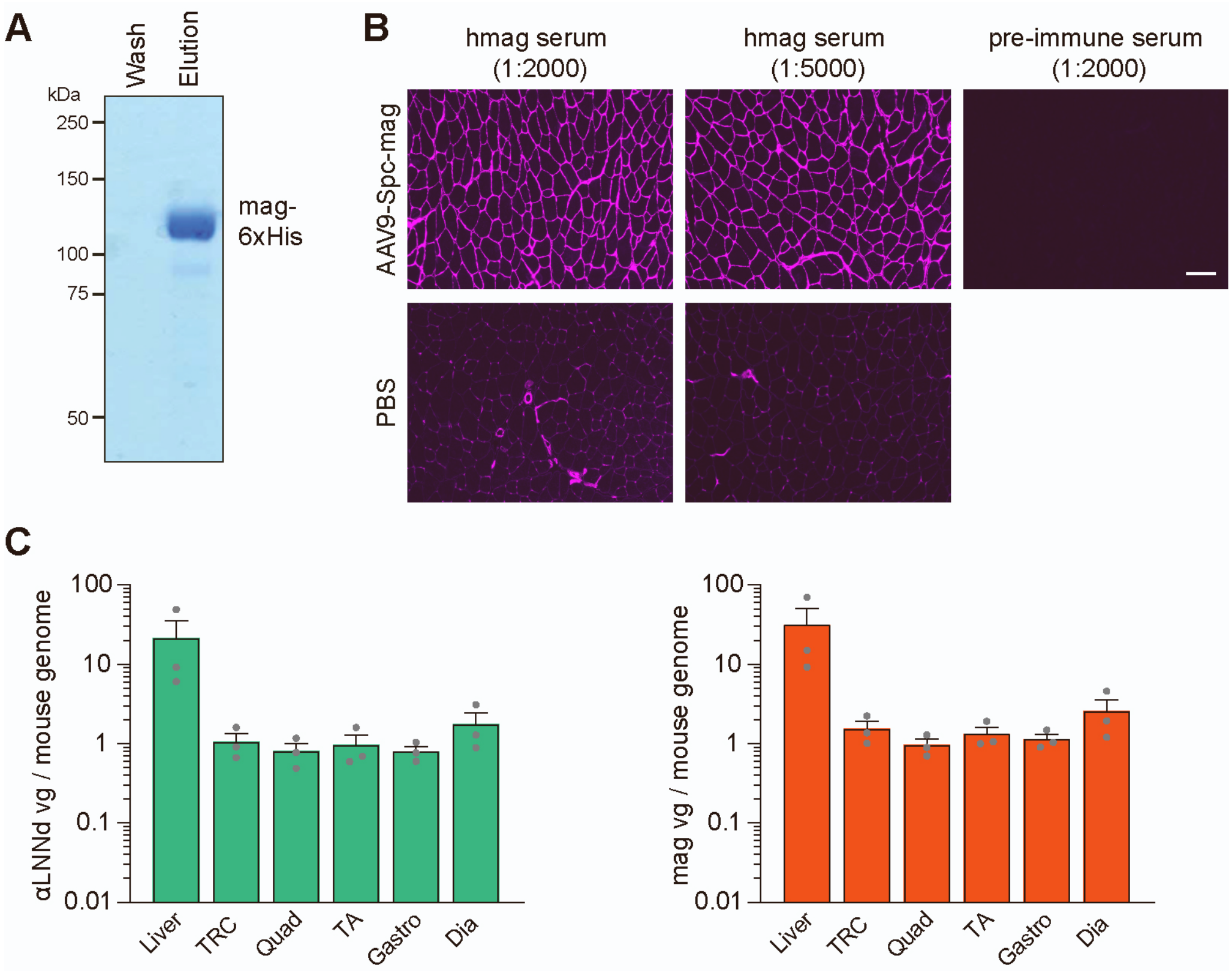

Figure S1. Generation of anti-human mag antisera and assessment of AAV9-Spc-DL systemic transduction efficacy. (**A**) Coomassie Blue-stained SDS-PAGE of wash and eluate fractions from Ni-NTA columns loaded with supernatant from transiently transfected HEK293 cells expressing mag-6×His. (**B**) Immunofluorescence images of triceps muscle cross-sections from 8-week-old C57BL/6 mice injected intravenously at P1 with AAV9-Spc-mag (1.5×10^{14} vg/kg) or vehicle (PBS). Sections were stained using serum (various dilutions) from a rabbit immunized with mag or with pre-immune serum from the same rabbit. (**C**) Quantification of vector genomes (vg) normalized to mouse genomic DNA by qPCR from different tissues of 8-week-old C57BL/6 mice injected at P1 with AAV9-Spc-αLNNd and AAV9-Spc-mag (1.5×10^{14} vg/kg per construct). Abbreviations are: Triceps brachii (TRC), quadriceps (Quad), tibialis anterior (TA), gastrocnemius (Gastro) and diaphragm (Dia). Data are presented as mean ± SEM. Scale bar: 100 µm. *n* = 3 mice per group.

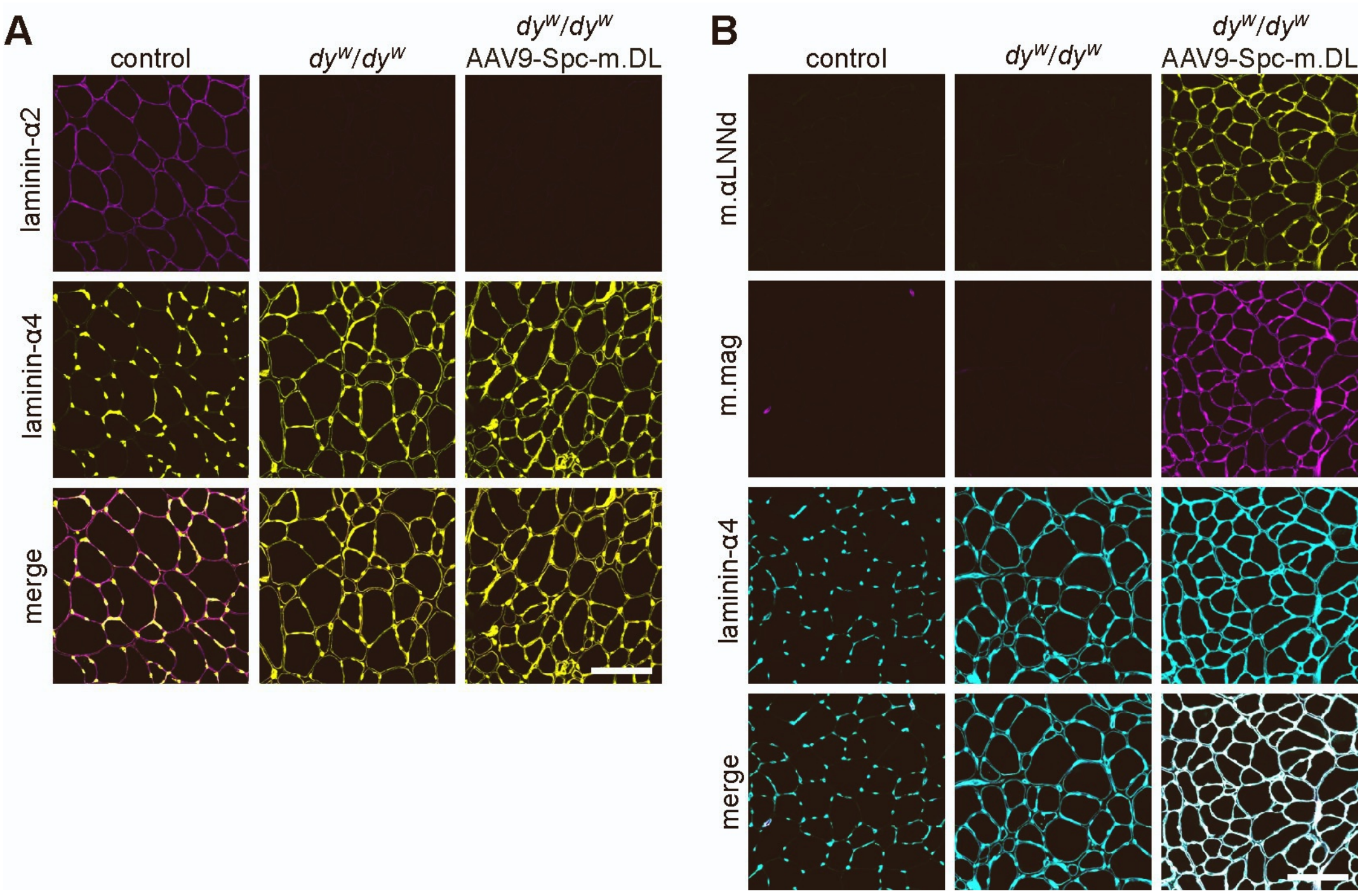

Figure S2. Laminin and linker protein localization in dy^W/dy^W mice and dy^W/dy^W AAV9-Spc-m.DL mice.
(**A-B**) Representative immunofluorescence images of cross-sections form TA muscle of 8-week-old non-injected healthy controls, untreated dy^W/dy^W mice, and dy^W/dy^W mice injected intravenously at P1 with AAV9-Spc-m.DL (1.5×10^{14} vg/kg per construct). Cross-sections were stained for laminin-α2 and laminin-α4 (**A**), or for m.αLNNd, m.mag and laminin-α4 (**B**). Note that laminin-α4 localizes to blood vessels in healthy controls but appears in the muscle fiber ECM in dy^W/dy^W mice and in dy^W/dy^W mice injected with AAV9-Spc-m.DL. Scale bars: 100 µm

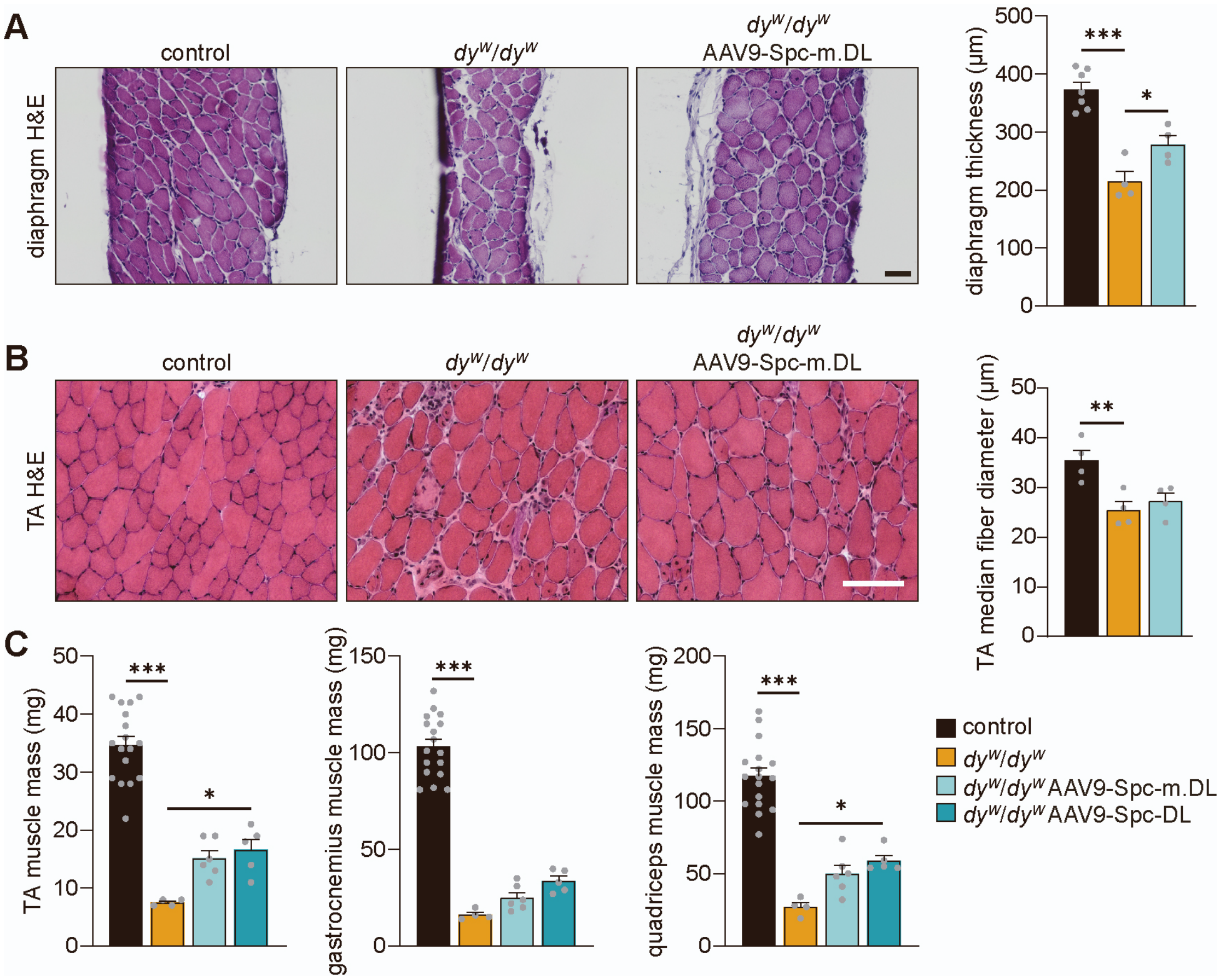

Figure S3. Therapeutic benefit of AAV-mediated, muscle-targeted expression of linker proteins in *dy^W/dy^W* mice. (**A-C**) Histology and muscle mass of non-injected healthy control, *dy^W/dy^W* mice, and *dy^W/dy^W* mice injected intravenously at P1 with AAV9-Spc-m.DL or the corresponding human linker constructs (AAV9-Spc-DL) (1.5×10^{14} vg/kg per construct). (**A-B**) Representative H&E-stained cross-sections of diaphragm (**A**) or TA (**B**) from 8-week-old mice, with quantification of diaphragm thickness (**A**) and TA median fiber diameter (**B**). (**C**) Muscle mass in 8-week-old mice. Data are presented as mean ± SEM. $^*P < 0.05$, $^{**}P < 0.01$, $^{***}P < 0.001$ by one-way ANOVA with Dunnett's post hoc test, comparing each group to untreated *dy^W/dy^W* mice. Scale bars: 50 µm (A), 100 µm (B). *n* = 4-17 mice per group.

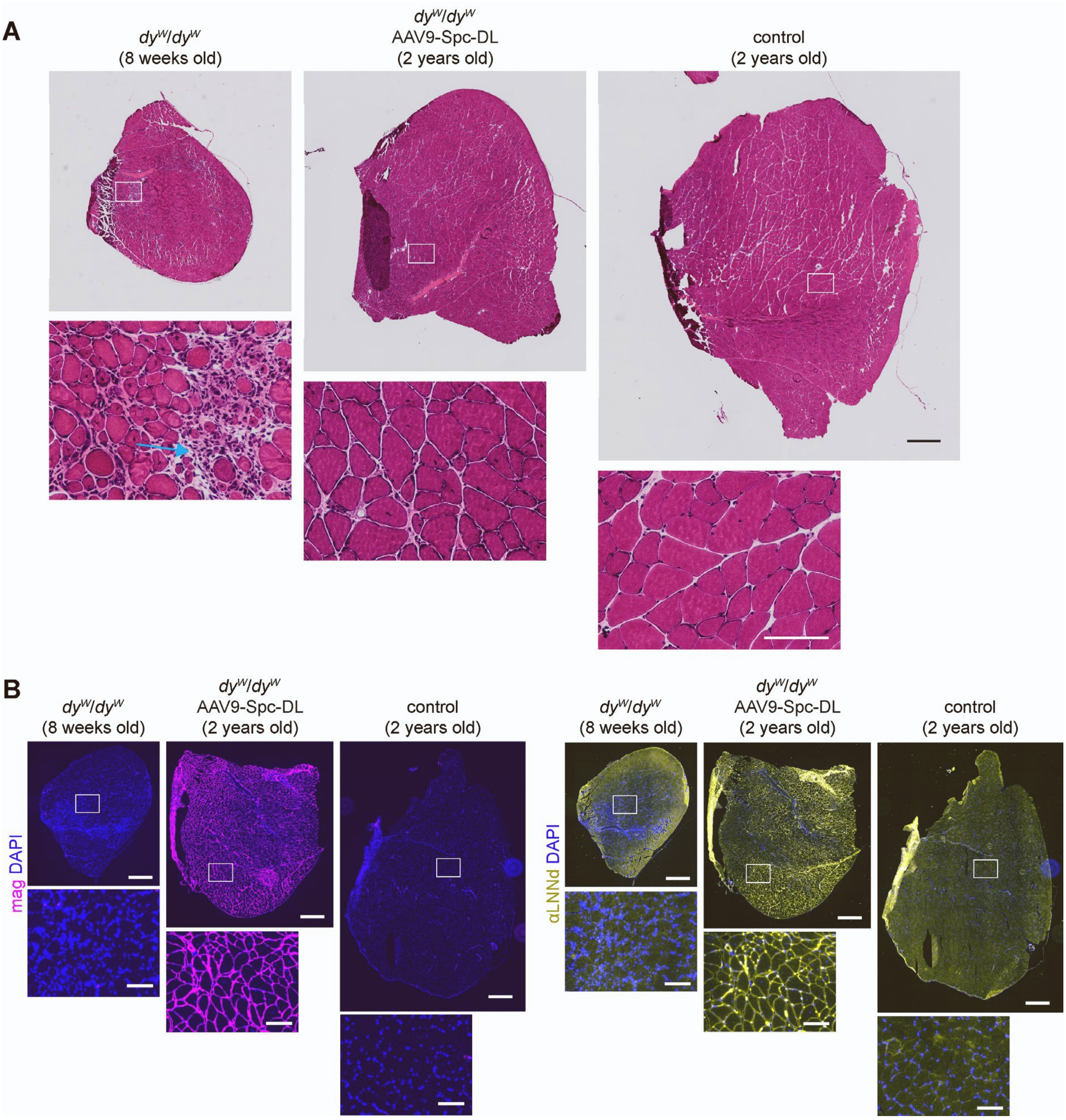

Figure S4. Long-term expression and sustained therapeutic benefit of AAV-mediated, muscle-targeted expression of linker proteins in dy^W/dy^W mice.

(**A-B**) Histology and immunofluorescence of non-injected healthy control, dy^W/dy^W mice, and dy^W/dy^W mice injected intravenously at P1 with AAV9-Spc-DL (1.5×10^{14} vg/kg per construct). (**A**) Representative H&E staining of triceps muscle cross-sections from an 8-week-old dy^W/dy^W mouse, a 2-year-old dy^W/dy^W mouse injected with AAV9-Spc-DL and a 2-year-old healthy control. Notably, muscle from untreated mice dy^W/dy^W showed mononuclear cell infiltrates (arrow), which were absent in the treated dy^W/dy^W mouse. (**B**) Immunofluorescence images stained for mag and αLNNd. Scale bars: 500 µm (A and B, top) and 100 µm (A and B, bottom).

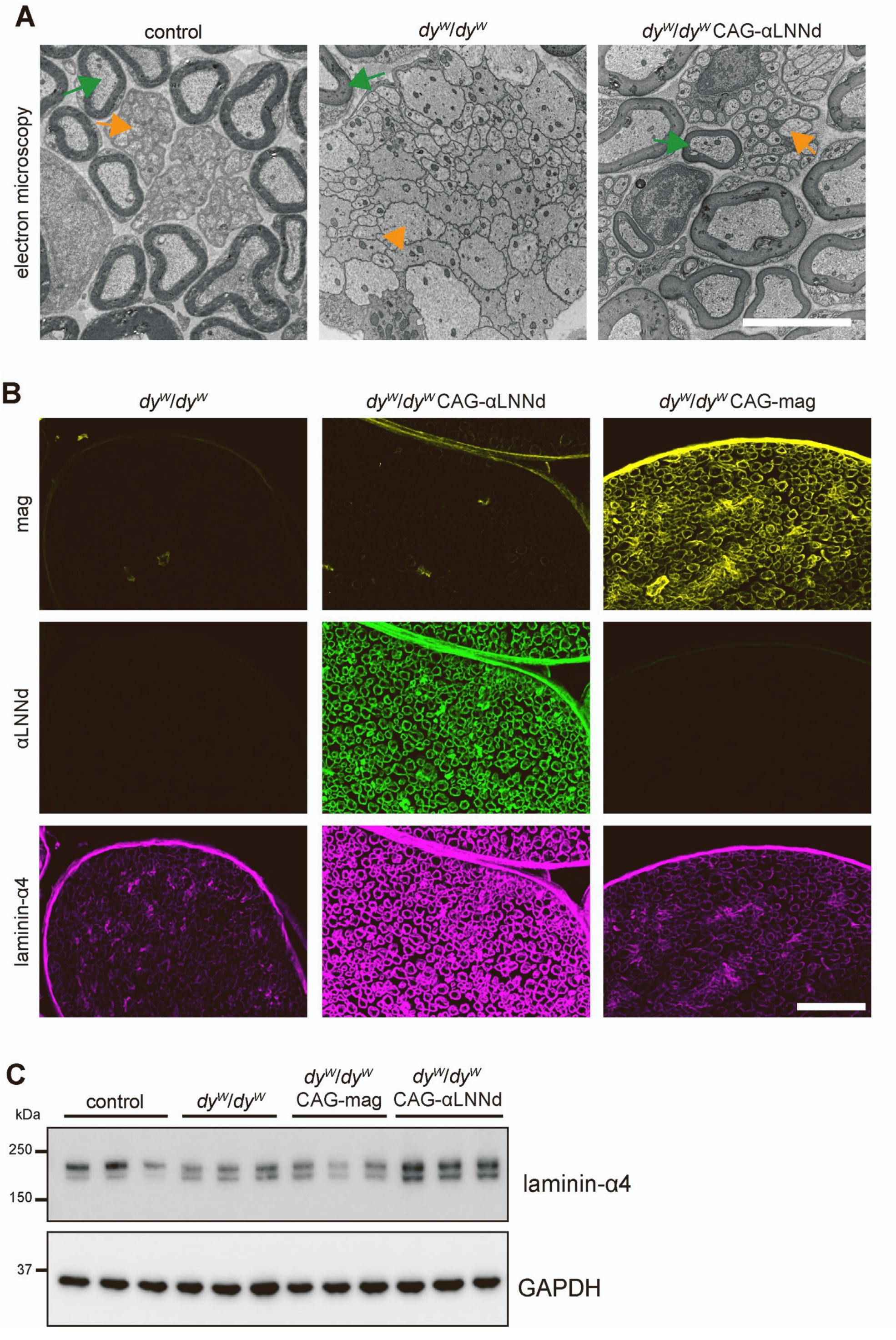

Figure S5. Restoration of axonal sorting and increase in laminin-α4 in sciatic nerve following ubiquitous transgenic expression of αLNNd.

(**A-C**) Analysis of sciatic nerve pathology and laminin-α4 levels in 8-week-old healthy control mice, *dy*^{W}/*dy*^{W} mice, and *dy*^{W}/*dy*^{W} transgenic mice that ubiquitously express mag or αLNNd under the control of the CAG promoter (CAG-mag or CAG-αLNNd). (**A**) Representative images of transmission electron microscopy of sciatic nerve from mice with the indicated genotypes. Note that in healthy controls, large-caliber motor axons are typically myelinated (green arrows), whereas small-diameter sensory axons are unmyelinated, ensheathed by non-myelinating Schwann cells, and clustered in Remak bundles (orange arrows). In contrast, *dy*^{W}/*dy*^{W} mice display large regions of unsorted, non-myelinated axons, including large-caliber axons (orange arrowhead) not ensheathed by myelinating Schwann cells. In *dy*^{W}/*dy*^{W} mice expressing CAG-αLNNd, axonal sorting appears similar to that observed in healthy controls with myelinated large axons (green arrow) and Remak bundles (orange arrow). (**B**) Immunofluorescence images of sciatic nerve cross-sections stained for mag, αLNNd and laminin-α4. Please note the strong increase in laminin-α4 in *dy*^{W}/*dy*^{W} CAG-αLNNd mice compared to the other two genotypes. (**C**) Western blot analysis of laminin-α4 in sciatic nerve lysates. GAPDH was used as loading control. Scale bars: 5 µm (A), 50 µm (B).

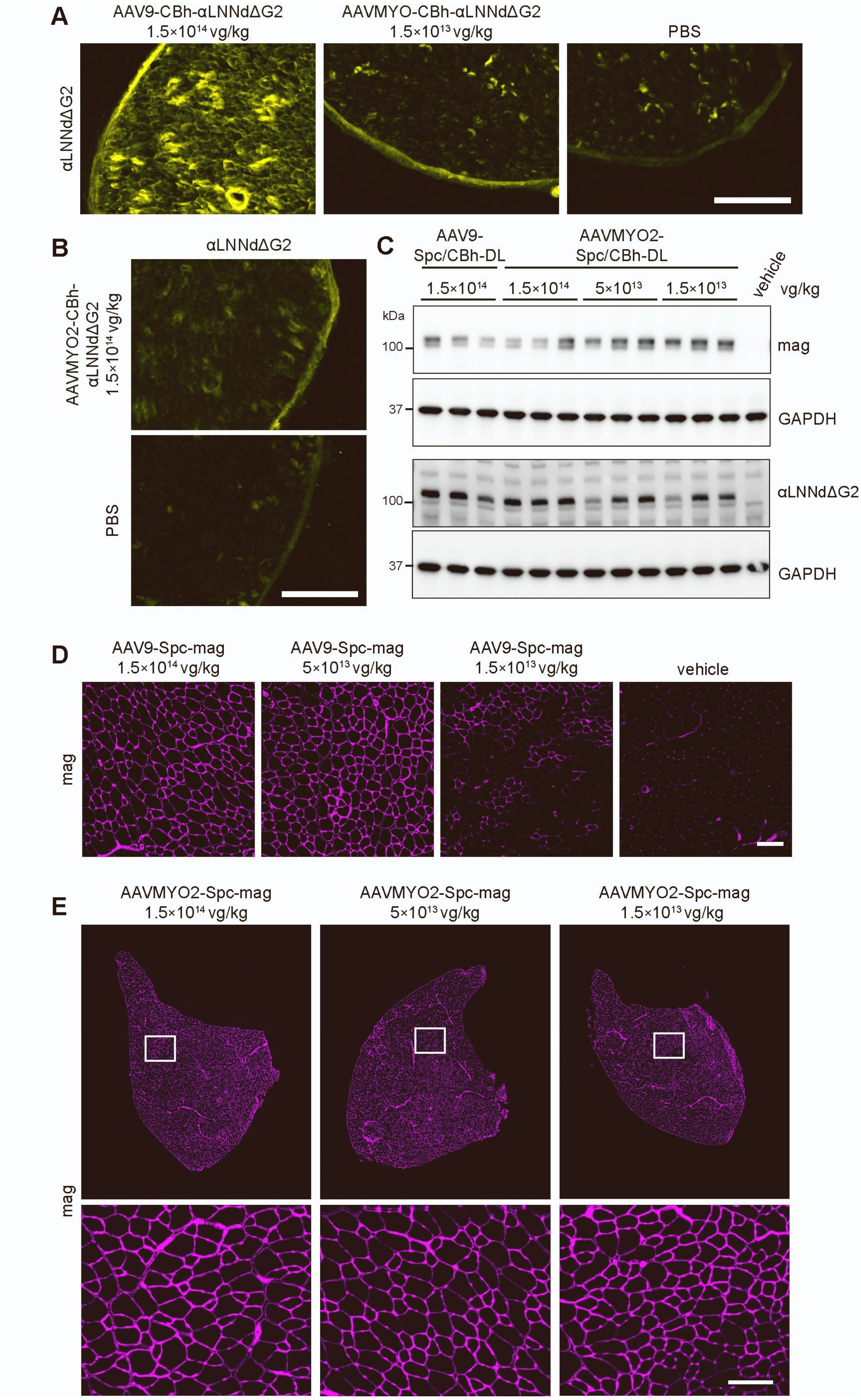

Figure S6. Comparison of AAVMYO, AAVMYO2, and AAV9-mediated expression in sciatic nerve and skeletal muscle. (**A-D**) Analysis of linker protein amount in 8-week-old C57BL/6 mice injected intravenously at P1 with the indicated construct and doses. (**A-B**) Immunofluorescence images of sciatic nerve cross-sections stained for αLNNdΔG2. Notably, only AAV9-mediated transduction resulted in αLNNdΔG2-positive basement membranes within the endoneurium. Some background staining was also observed in axons of PBS-injected mice. (**C**) Western blot analysis of triceps muscle lysate to detect αLNNdΔG2 or mag. GAPDH was used as loading control. (**D-E**) Immunofluorescence staining for mag of TA muscle cross-sections. Scale bars: 50 µm (A, B), 100 µm (D, E).

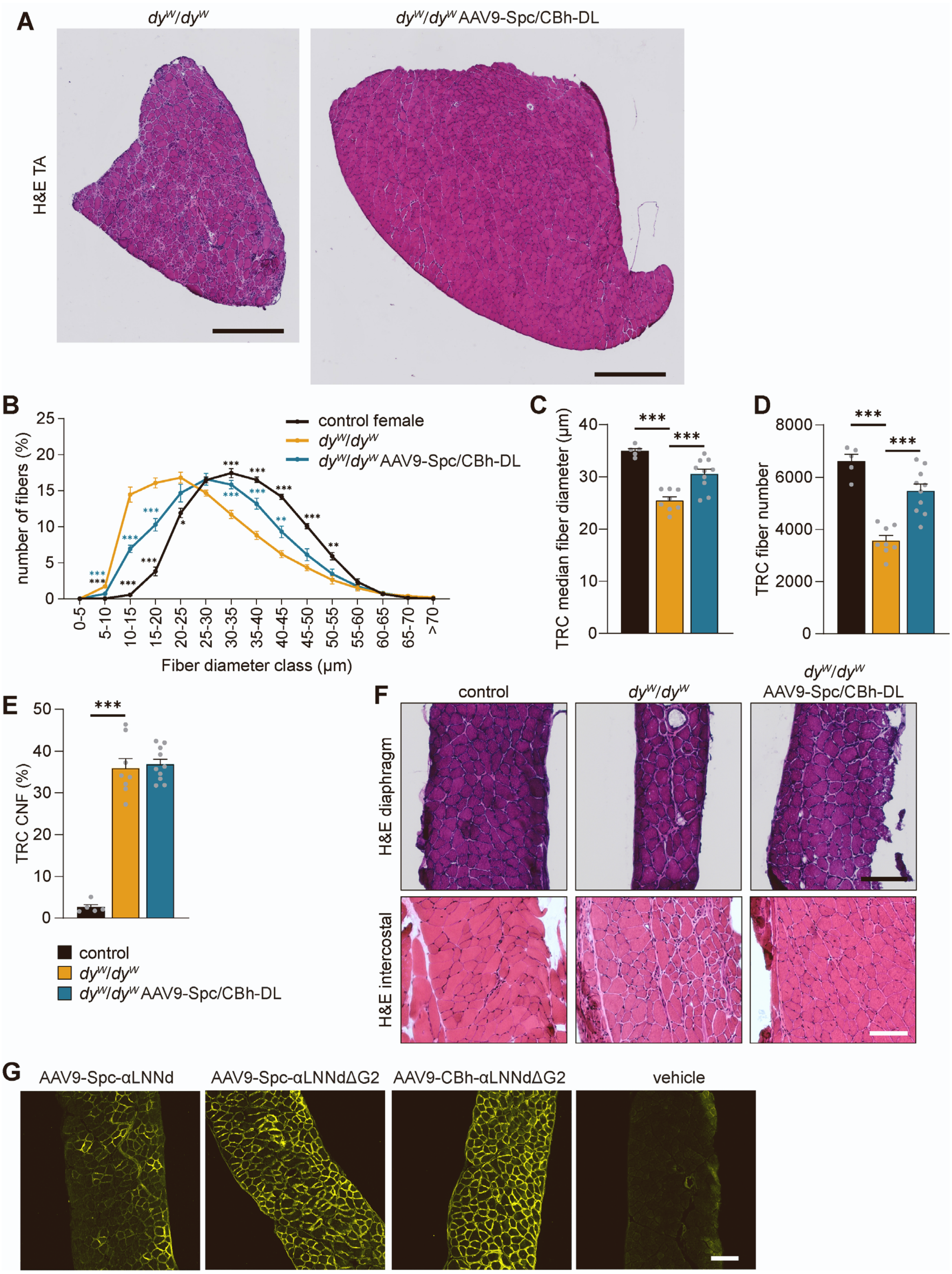

Figure S7. AAV9-mediated improvement of histology in TA, triceps and respiratory muscles in *dy^W/dy^W* AAV9-Spc/CBh-DL mice.

(**A-F**) Histological analysis of skeletal muscle from 8-week-old healthy control, *dy^W/dy^W* and *dy^W/dy^W* mice injected intravenously at P1 with AAV9-Spc/CBh-DL (1×10^{14} vg/kg per construct). (**A**) Representative H&E-stained cross-sections of TA muscle. (**B-E**) Quantification of triceps muscle histology. (**B**) Distribution of muscle fiber diameters. (**C**) Median muscle fiber diameter. (**D**) Total number of muscle fibers. (**E**) Percentage of fibers with centralized nuclei. (**F**) Representative H&E-stained cross-sections of diaphragm and intercostal muscles. (**G**) Immunofluorescence images of diaphragm cross-sections from 8-week-old mice stained for αLNNd or αLNNdΔG2 using antibodies against the LN domain of laminin-α1. Mice were injected intravenously at P1 with the indicated constructs (1.5×10^{14} vg/kg per construct). Data are presented as mean ± SEM. *P < 0.05, **P < 0.01, ***P < 0.001 by one-way ANOVA with Dunnett's post hoc test, comparing each group to untreated *dy^W/dy^W* mice. Scale bars: 500 µm (A), 100 µm (F, G). *n* = 3-10 mice per group.

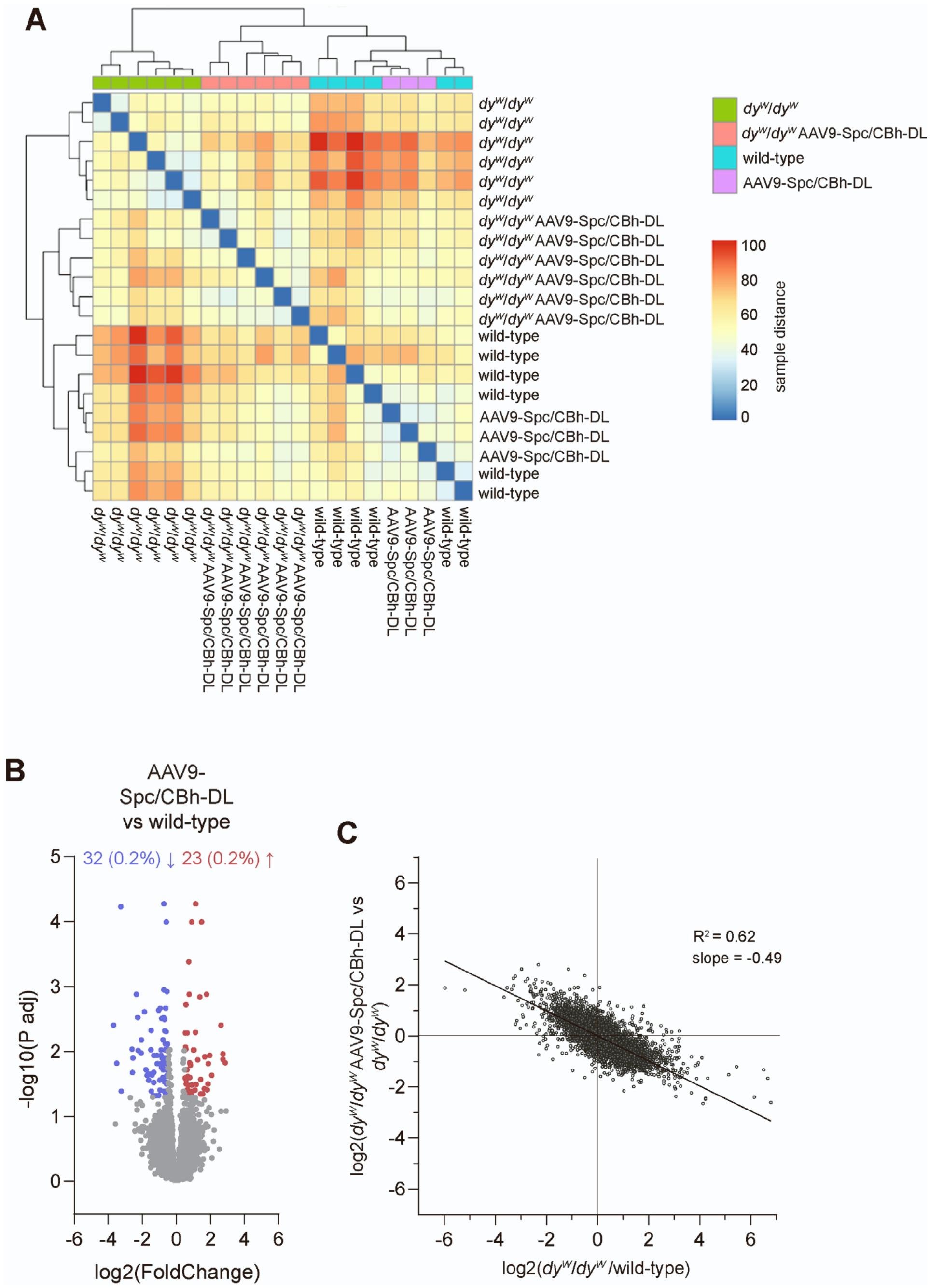

Figure S8. RNA-seq analysis of transcriptional changes by AAV9-mediated expression of mag and αLNNdΔG2 in ***dy^W/dy^W*** **mice and wild-type controls.**

(**A-E**) RNA-seq analysis of TA muscle from 8-week-old mice: non-injected wild-type and dy^W/dy^W mice, and wild-type and dy^W/dy^W mice injected intravenously at P1 with AAV9-Spc/CBh-DL (1×10^{14} vg/kg per construct). (**A**) Sample correlation blot of RNA-seq data. (**B**) Volcano plot showing the 14,905 transcripts detected in wild-type mice injected with AAV9-Spc/CBh-DL versus non-injected wild-type mice. Differentially expressed genes ($\log_2$ fold change > ±0.5; adjusted $p < 0.05$) are shown in red (upregulated) or blue (downregulated). Numbers above indicate the total number and percentage of upregulated (red) or downregulated (blue) transcripts. (**C**) Pairwise comparison of gene expression changes: Fold change in dy^W/dy^W versus wild-type mice plotted against fold change in dy^W/dy^W AAV9-Spc/CBh-DL versus untreated dy^W/dy^W mice. *n* = 3-6 mice per group.

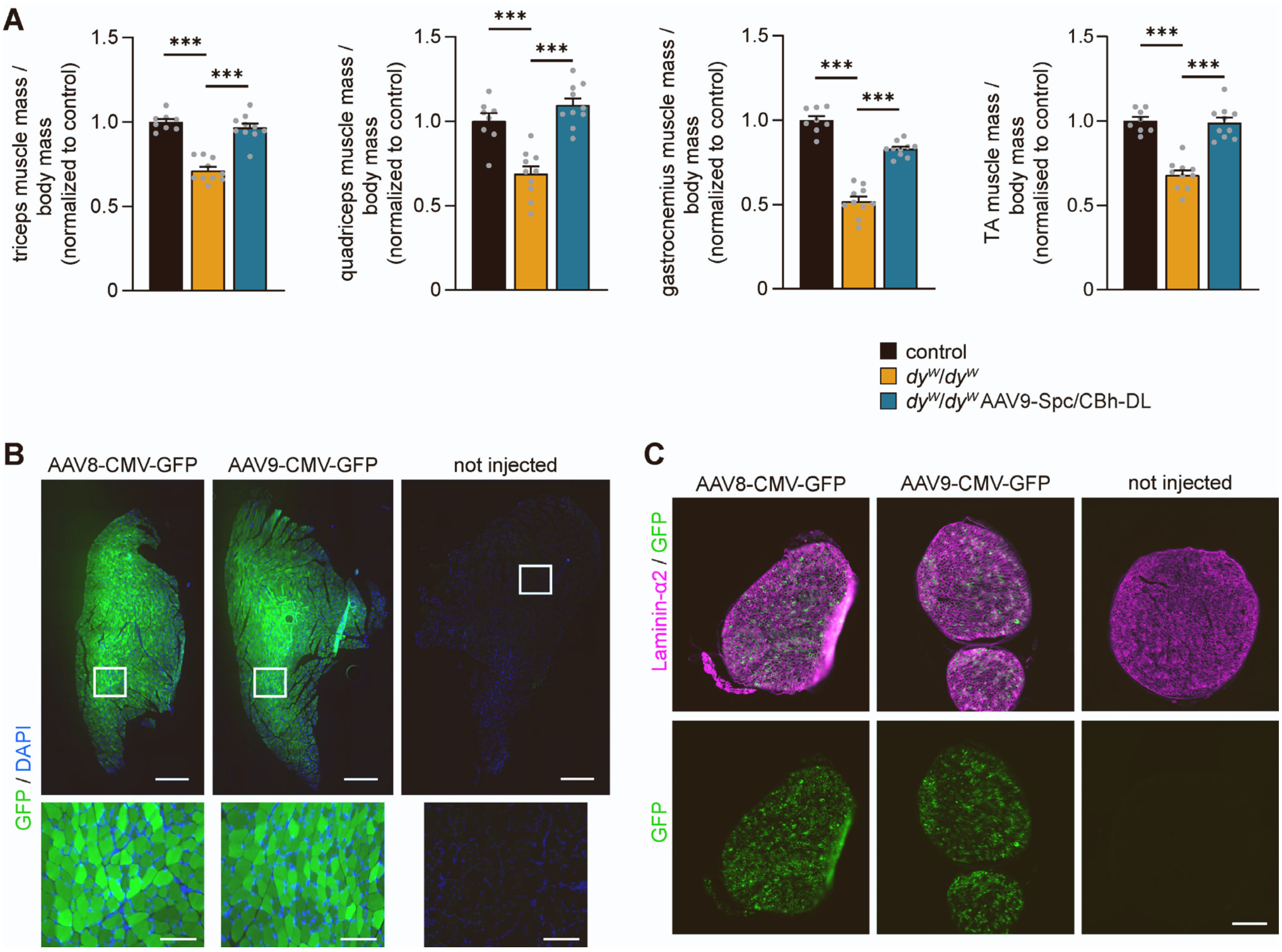

Figure S9. Comparison of AAV9 and AAV8 transduction efficiency in muscle and peripheral nerve. (**A**) Muscle mass normalized to body mass of 8-week-old wild-type, *dy^{W}/dy^{W}* or *dy^{W}/dy^{W}* mice intravenously injected at postnatal day 1 (P1) with AAV9-Spc/CBh-DL (1×10¹⁴ vg/kg per construct). (**B-C**) C57BL/6 mice were injected intravenously at P1 with CMV-GFP packaged into AAV9 or AAV8 (1×10¹⁴ vg/kg). Tissue was analyzed at 4 weeks of age. (**B**) Immunofluorescence images of TA muscle cross-sections stained for GFP. (**C**) Immunofluorescence images of sciatic nerve cross-sections stained for laminin-α2 and GFP. Data are presented as mean ± SEM. ****P* < 0.001 by one-way ANOVA with Dunnett's post hoc test, comparing each group to untreated *dy^{W}/dy^{W}* mice. Scale bars: 500 µm (B, top) and 100 µm (B, bottom and C).

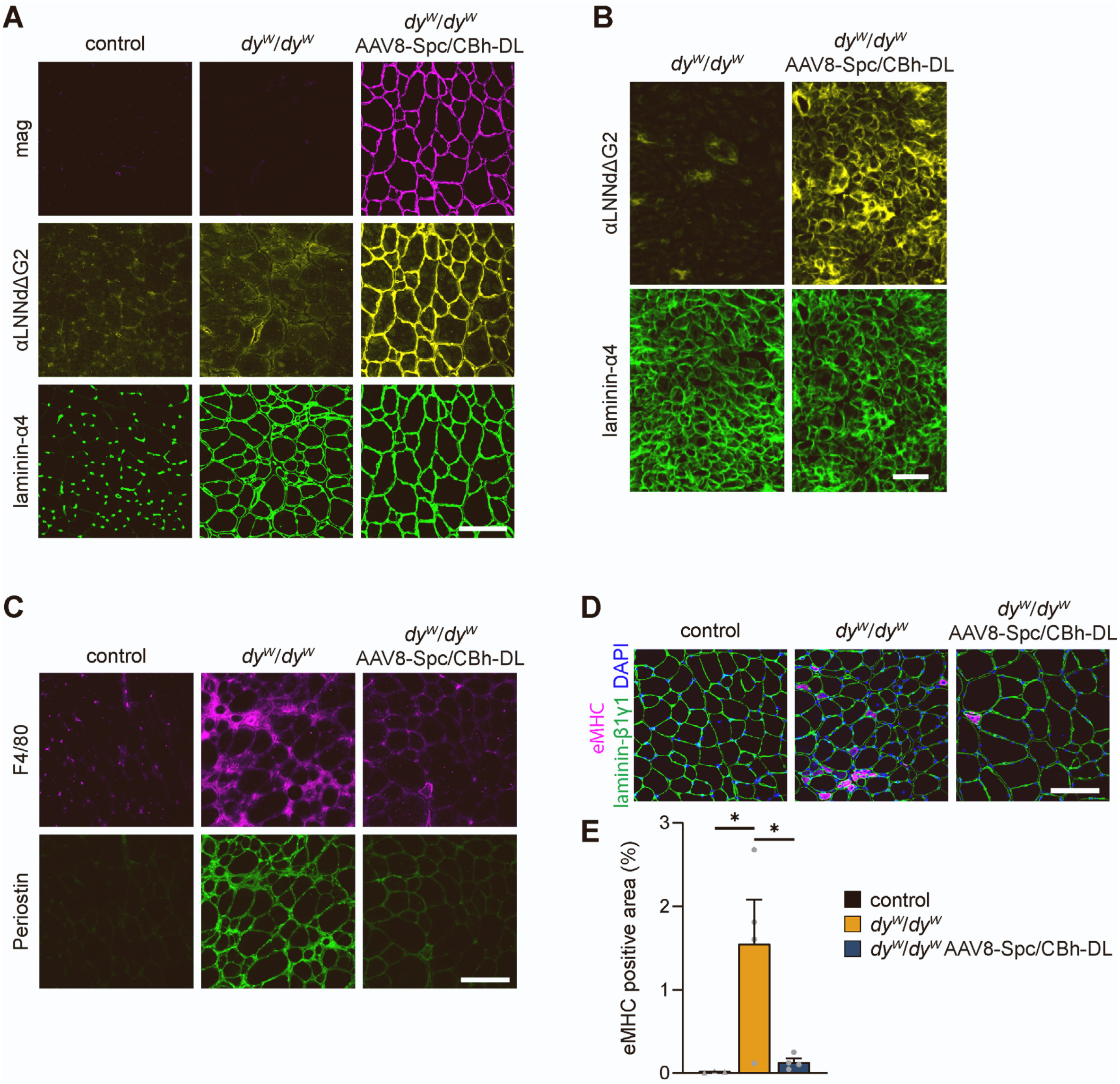

Figure S10. Improvements in degeneration/regeneration, inflammation and fibrosis by AAV8-mediated co-expression of mag in muscle and αLNNdΔG2 in muscle and nerve.
(**A-D**) Representative immunofluorescence images of TA cross-sections (A, C and D) or sciatic nerve (B) from 8-week-old heathy control, dy^W/dy^W or dy^W/dy^W mice injected at P1 with AAV8-Spc/CBh-DL ($1×10^{14}$ vg/kg per construct). Cross-sections were stained for mag, αLNNdΔG2 and laminin-α4 (**A-B**); F4/80 (macrophage marker), Periostin (fibrosis marker) (**C**); eMHC and laminins (β1γ1-chains) (**D**). (**E**) Quantification of eMHC-positive area. Data are presented as mean ± SEM. **P* < 0.05 by one-way ANOVA with Dunnett's post hoc test, comparing each group to untreated dy^W/dy^W mice. Scale bars: 100 µm (A, C, D) and 20 µm (B). *n* = 3-4 mice per group.

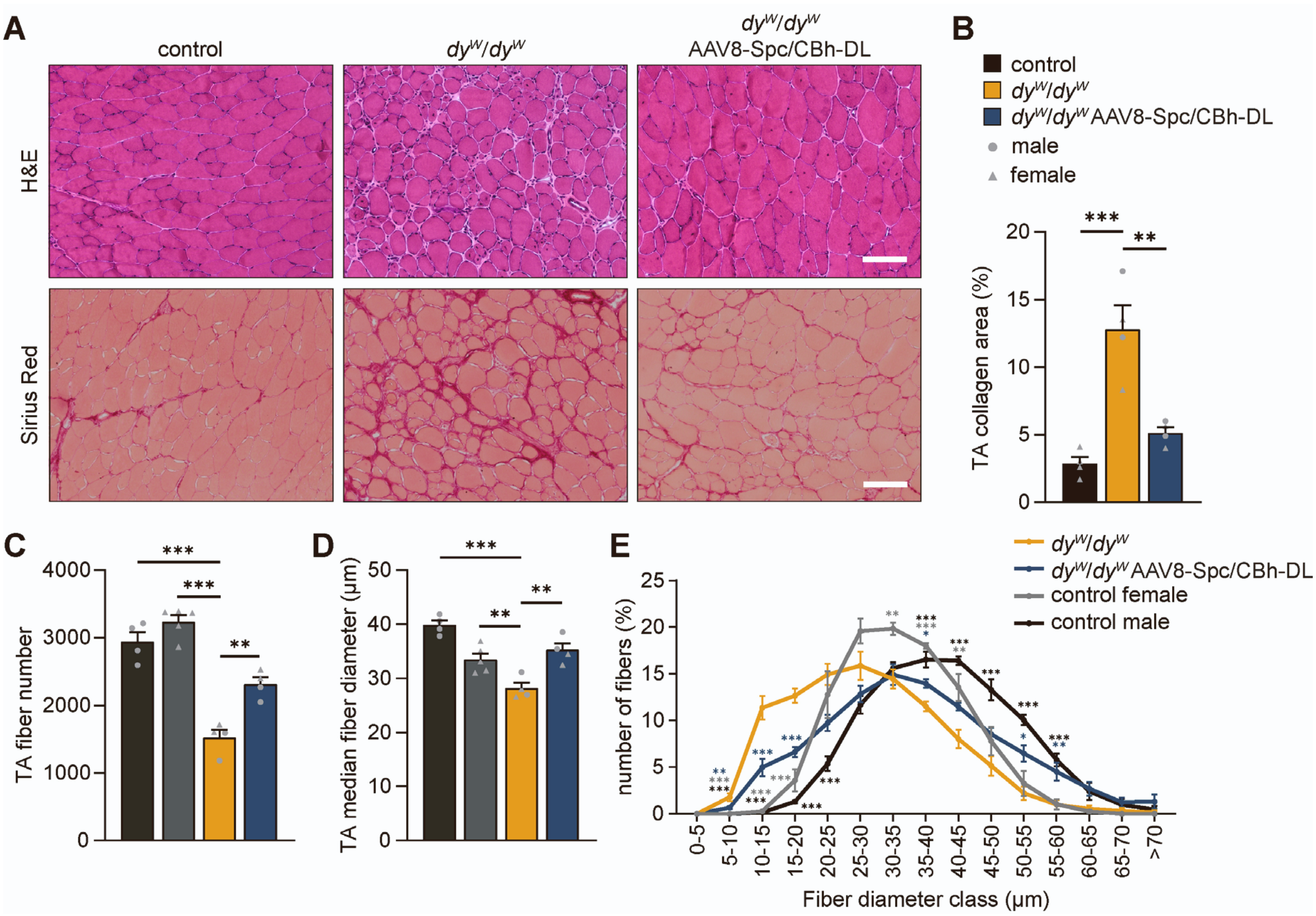

Figure S11. Improvements of TA muscle histology by AAV8-mediated co-expression of mag in muscle and αLNNdΔG2 in muscle and nerve.

(**A-E**) Analysis of TA muscle histology from 8-week-old wild-type, *dy^W/dy^W* or *dy^W/dy^W* mice injected at P1 with AAV8-Spc/CBh-DL ($1×10^{14}$ vg/kg per construct). (**A**) Representative images of H&E- and Sirius Red-stained TA muscle cross-sections. (**B**) Quantification of muscle fibrosis based on Sirius Red-positive area in TA cross-sections. (**C**) Total muscle fiber number. (**D**) Median muscle fiber diameters. (**E**) Distribution of muscle fiber diameters. Data are presented as mean ± SEM. ***P* < 0.01, ***P* < 0.01, ****P* < 0.001 by one-way ANOVA with Dunnett's post hoc test, comparing each group to untreated *dy^W/dy^W* mice. Scale bar: 100 µm. *n* = 4 mice per group.

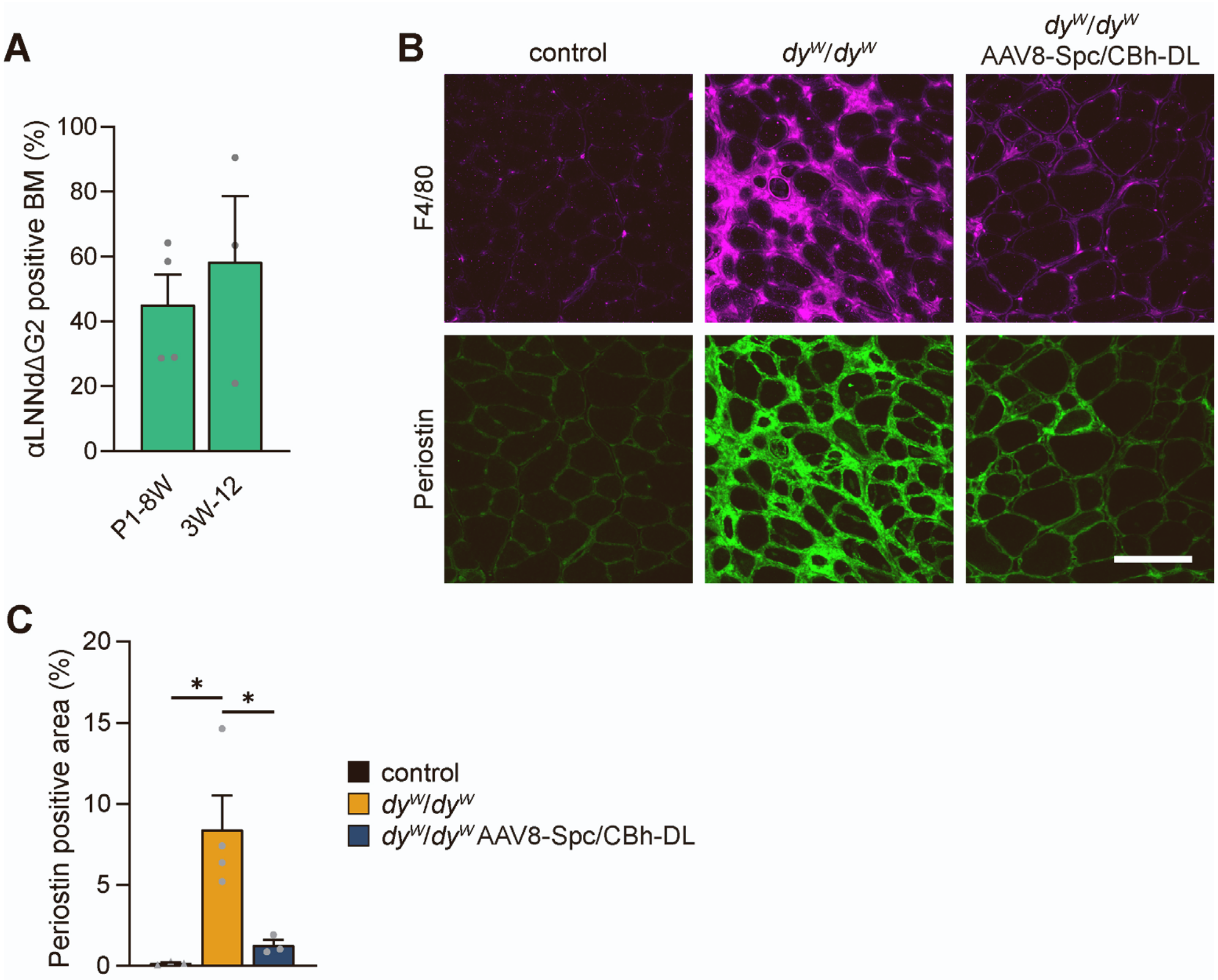

Figure S12. Linker protein basement membrane coverage in peripheral nerve and histological improvements with late treatment.
(**A**) Quantitative analysis of αLNNdΔG2 basement membrane (BM) coverage in sciatic nerve cross-sections based on immunostaining (representative images shown in Figure S10B and Figure 8C). BM coverage was measured as the percentage of laminin-α4–positive endoneurial BM that also contained αLNNdΔG2 following either P1 injection with 1×10^{14} vg/kg and analysis at 8 weeks of age, or treatment at 3 weeks of age with 5×10^{13} vg/kg AAV8-Spc/CBh-DL and analysis at 12 weeks of age. (**B**) Representative immunofluorescence images of TA muscle cross-sections from 12-week-old heathy control, *dy*^{W}/*dy*^{W} or *dy*^{W}/*dy*^{W} mice injected at the age of 3 weeks with AAV8-Spc/CBh-DL (5×10^{13} vg/kg per construct). Cross-sections were stained for F4/80 (macrophage marker) and Periostin (fibrosis marker) (**C**) Quantification of Periostin-positive area. Data are presented as mean ± SEM. *$P < 0.05$ by one-way ANOVA with Dunnett's post hoc test, comparing each group to untreated *dy*^{W}/*dy*^{W} mice. Scale bar: 100 µm. n = 3-4 mice per group.

Video S1: Phenotype of 5-week-old *dy^W/dy^W* mice treated with AAV9-Spc-m.DL

Representative video of a 5-week-old healthy wild-type, untreated *dy^W/dy^W* mouse and *dy^W/dy^W* mouse intravenously injected at P1 with AAV9-Spc-m.αLNNd and AAV9-Spc-m.mag (together referred to as AAV9-Spc-m.DL) at a dose of 1.5×10^{14} vg/kg per construct.

Video S2: Hindlimb paralysis in 8-month-old *dy^W/dy^W* mouse treated with AAV9-Spc-DL

Representative video of an 8-month-old *dy^W/dy^W* mouse intravenously injected at P1 with AAV9-Spc-DL (1.5×10^{14} vg/kg per construct).

Video S3: No signs of peripheral neuropathy in 8-month-old *dy^W/dy^W* mouse transgenically expressing αLNNd under a ubiquitous promoter

Representative video of an 8-month-old *dy^W/dy^W* CAG-αLNNd transgenic mouse showing normal gait and no evidence of peripheral neuropathy.

Video S4: Peripheral neuropathy in 8-week-old *dy^W/dy^W* mouse transgenically expressing mag under a ubiquitous promoter

Representative video of 8-week-old *dy^W/dy^W* CAG-mag transgenic mouse, displaying signs of peripheral neuropathy.

Video S5: Close-to-normal phenotype in *dy^W/dy^W* mice treated with AAV9-Spc/CBh-DL

Representative video of 8-week-old wild-type, *dy^W/dy^W* and *dy^W/dy^W* mice injected intravenously at P1 with AAV9-Spc/CBh-DL (1×10^{14} vg/kg per construct).

Video S6: Improved neuromuscular phenotype by late treatment

Representative movie of 12-week-old untreated *dy^W/dy^W* mice and *dy^W/dy^W* mice injected intravenously at 3 weeks of age with AAV8-Spc/CBh-DL (5×10^{13} vg/kg per construct). Treated mice show improved motor performance.
