# Supplementary material for: Dual AAV gene therapy using laminin-linking proteins ameliorates muscle and nerve defects in LAMA2-related muscular dystrophy
Source: Mol Ther. 2026 Feb 2;34(5):2818–39. doi: 10.1016/j.ymthe.2026.01.041 (PMC13154268; doi:10.1016/j.ymthe.2026.01.041)
Supplement: Supplementary file 2 [file mmc8.pdf]

Molecular Therapy
Original Article

Dual AAV gene therapy using laminin-linking proteins ameliorates muscle and nerve defects in LAMA2-related muscular dystrophy

Judith R. Reinhard,[1] Shuo Lin,[1] Eleonora Maino,[1] Daniel J. Ham,[1] and Markus A. Rüegg[1]

[1]Biozentrum, University of Basel, 4056 Basel, Switzerland

Adeno-associated virus (AAV)-mediated gene replacement holds promise for treating genetic diseases but faces challenges due to the limited packaging capacity and potential immune responses to transgene products, especially in patients lacking endogenous protein. LAMA2-related muscular dystrophy (LAMA2 MD), a severe congenital disorder caused by loss of laminin-α2, presents both hurdles: the *LAMA2* gene exceeds AAV capacity, and severely affected patients do not produce the native protein. Here, we developed an AAV-based therapy using two engineered linker proteins derived from endogenously expressed components. These linker proteins restore laminin receptor binding and polymerization, enabling reassembly of a functional basement membrane. Dual AAV delivery of the linkers in a severe LAMA2 MD mouse model resulted in robust expression and significant improvements in muscle histology and function. Employing myotropic capsids enabled therapeutic efficacy at lower vector doses. However, muscle-specific targeting unmasked a LAMA2-related peripheral neuropathy. To address this, we expressed one linker under a muscle-specific promoter and the other under a ubiquitous promoter, delivered via AAV9 or AAV8. This approach achieved near-complete phenotypic restoration when administered neonatally and provided significant benefit when given at progressed disease stages. Our strategy offers a mutation-independent, size-compatible, and potentially immune-tolerable treatment for LAMA2 MD with broad clinical potential.

INTRODUCTION

Laminins are a family of extracellular matrix (ECM) glycoproteins essential for the structural and functional integrity of tissues. As key components of basement membranes, they provide mechanical support, facilitate cell adhesion, and contribute to tissue organization and stability.[1] Laminins are heterotrimeric proteins composed of one α, one β, and one γ chain, encoded by distinct genes. In mammals, five α (*LAMA1-5*), four β (*LAMB1–4*), and three γ (*LAMC1–3*) chains have been identified, which can assemble into at least 15 distinct isoforms in a tissue- and development-specific manner.[2] The laminin-α2 chain, encoded by *LAMA2*, typically assembles with either a β1 or β2 chain and a γ1 chain, forming heterotrimers collectively referred to here as laminin-2X1 (α2, β1/2, γ1). The laminin-α2-containing isoforms, originally known as merosin, were identified to be predominantly expressed in Schwann cells and striated muscle.[3]

Recessive loss-of-function mutations in *LAMA2*, which encodes the laminin-α2 chain, cause LAMA2-related muscular dystrophy (LAMA2 MD). The disease was first identified as congenital early-onset severe muscular dystrophy.[4] Today, it is clear that the disease exists in two forms. The severe, early-onset congenital form presents within the first 6 months of life and is typically caused by bi-allelic null mutations resulting in the complete absence of laminin-α2. The milder, late-onset form is often associated with hypomorphic mutations that allow some residual protein expression.[5,6] Clinically, LAMA2 MD manifests with progressive muscle wasting, motor impairments, respiratory insufficiency, and, in severe cases, early mortality.[7–9] Most early-onset patients do not achieve independent ambulation, while late-onset patients may attain and later lose the ability to walk.[6,7,10] Despite the clinical burden, no disease-modifying treatments are currently available.

Several mouse models recapitulate the human disease with varying severity. The dy^{2J}/dy^{2J} model mimics a late-onset phenotype and maintains normal survival, whereas dy^{W}/dy^{W} and dy^{3K}/dy^{3K} mice exhibit severe early-onset disease with severe muscle wasting and strongly reduced lifespan.[11] In addition to muscular dystrophy, all mouse models consistently present with pronounced peripheral neuropathy.[12] In human patients, peripheral neuropathy is less apparent. However, some studies have reported abnormalities in peripheral nerve conduction and morphology, supporting peripheral nerve involvement in the disease.[12]

Gene therapy offers a promising avenue for treating LAMA2 MD. However, the large size of the *LAMA2* coding sequence exceeds the packaging capacity of adeno-associated virus (AAV) vectors,

Received 8 August 2025; accepted 26 January 2026;
https://doi.org/10.1016/j.ymthe.2026.01.041.

Correspondence: Judith R. Reinhard, Biozentrum, University of Basel, 4056 Basel, Switzerland.
E-mail: judith.reinhard@unibas.ch
Correspondence: Markus A. Rüegg, Biozentrum, University of Basel, 4056 Basel, Switzerland.
E-mail: markus-a.ruegg@unibas.ch

Published by Elsevier Inc. on behalf of The American Society of Gene and Cell Therapy.
This is an open access article under the CC BY license (http://creativecommons.org/licenses/by/4.0/).

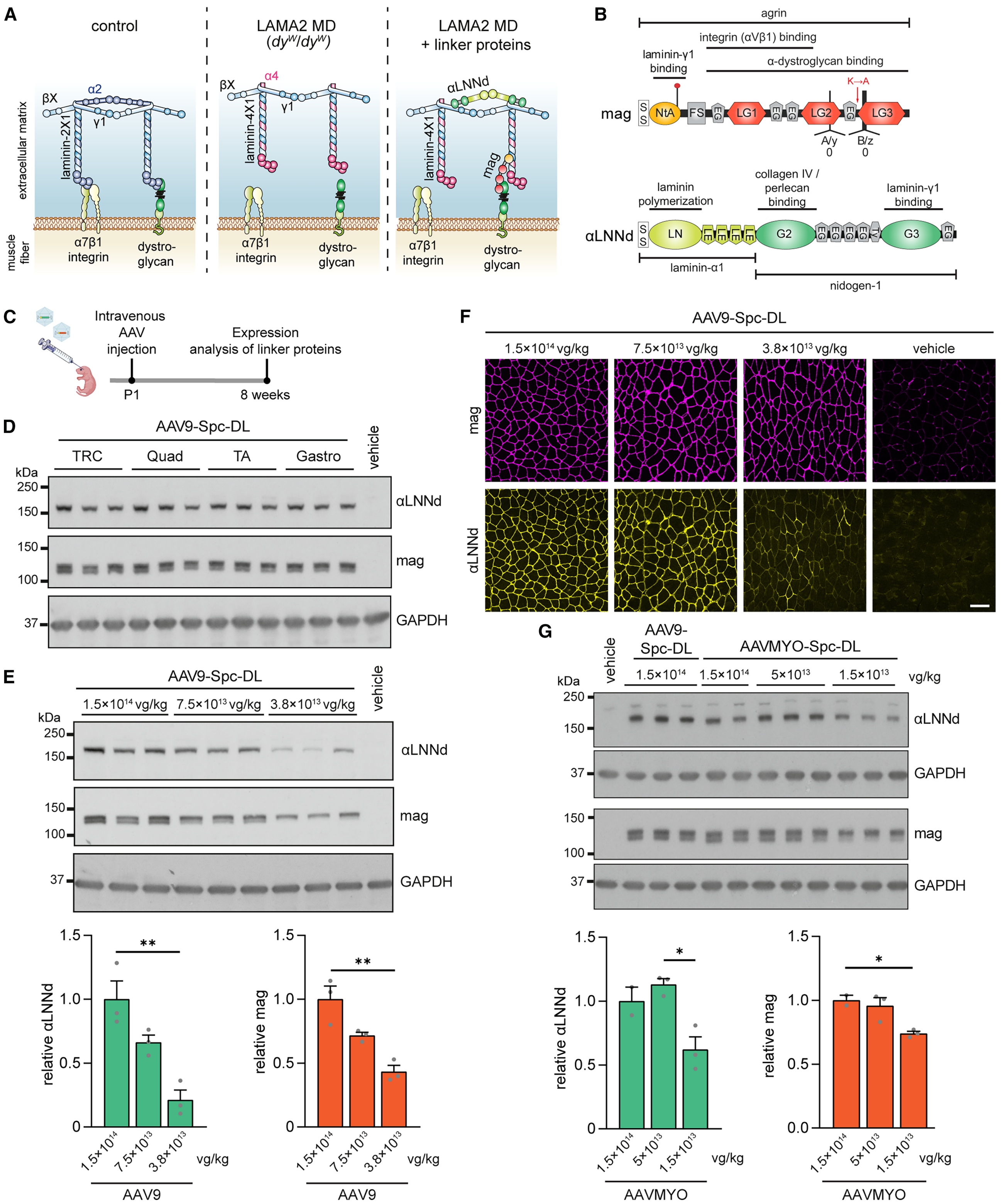

(legend on next page)

precluding direct gene replacement. Furthermore, immune responses to transgene products remain a significant challenge in neuromuscular gene therapy, especially in immunologically primed or cross-reactive immunologic material-negative patients.[13–16] In LAMA2 MD, severe early-onset cases present with extensive inflammatory infiltration,[17–19] which may exacerbate immune responses to exogenous proteins, particularly bacterial components, such as Cas9 or split-intein domains, used in gene editing or dual-vector strategies. Additionally, the intracellular assembly of laminin heterotrimers via coiled-coil domains complicates the design of functional mini-laminins or fusion constructs, as alterations at junction sites may interfere with proper trimerization and ECM incorporation.

To overcome these barriers, we developed a mutation-independent gene therapy strategy that leverages the compensatory upregulation of laminin-α4 observed in LAMA2 MD patients.[20,21] In the absence of laminin-α2, muscle tissue express laminin-4X1 (α4,β1/2,γ1), which is properly assembled and secreted but functionally inadequate. Laminin-4X1 cannot polymerize or bind α-dystroglycan and α7β1 integrin that normally anchor laminin-2X1 to the muscle fiber surface.[21] To restore basement membrane function, we engineered two linker proteins composed of endogenously expressed domains of other ECM components. These linkers enable receptor binding and polymerization of laminin-4X1, effectively reconstituting a functional basement membrane when co-delivered via dual AAV vectors.

In the severe dy^W/dy^W mouse model, expression of the linkers via systemic AAV9 or AAV8 application led to widespread incorporation into the basement membrane and robust improvements in muscle histology, function, and transcriptional profiles. Importantly, due to their secreted nature, the linkers achieve broad tissue distribution without requiring very high total AAV doses. Using myotropic AAV capsids enabled efficient transduction at lower doses. In addition to the muscular dystrophy, we also targeted the peripheral neuropathy by expressing one linker by a ubiquitous promoter. This dual-tissue targeting ameliorated both muscular dystrophy and peripheral neuropathy. Importantly, the therapy was effective when administered neonatally and retained therapeutic benefit when delivered at later disease stages. By overcoming vector size constraints and reducing immune risks, our approach offers a promising, mutation-independent strategy with translational potential for the treatment of LAMA2 MD.

RESULTS

AAV-mediated muscle-specific expression of linker proteins improves muscle histology, body weight, and muscle function in dy^W/dy^W mice

Since the coding sequence of laminin-α2 is too large for gene replacement via AAVs, we aimed to restore laminin-α2 function by leveraging laminin-4X1, which is present in the basement membrane of laminin-α2-deficient patients and mouse models.[20,21] To achieve this, we used mini-agrin (mag) to anchor laminin-4X1 to the sarcolemma via α-dystroglycan, and αLNNd to enable laminin-4X1 polymerization (Figure 1A). Mag is a miniaturized form of the ECM proteoglycan agrin. It includes the N-terminal agrin domain, which binds to the coiled-coil region of laminins,[22] and a C-terminal part that binds with high affinity to α-dystroglycan.[23] In addition to the strong α-dystroglycan-binding domains, the mag construct retains agrin domains that interact with some integrin isoforms.[24–26] Notably, the C-terminal domains lack the amino acid inserts A/y and B/z, which are required for acetylcholine receptor clustering,[27,28] and a single amino acid substitution was introduced to prevent the proteolytic cleavage of mag. The second linker, αLNNd, consists of the LN domain of laminin-α1 fused to the laminin-γ1-binding domain of nidogen-1.[29] Both engineered linkers contained their endogenous signal peptides to ensure proper secretion (Figure 1B).

To assess the feasibility of AAV-mediated delivery, we generated AAV9 vectors encoding codon-optimized versions of human mag (mag) and human αLNNd (αLNNd). Given the large size of αLNNd (4.1 kb), we used the synthetic, small muscle-specific promoter Spc5.12 (Spc)[30,31] for both constructs. To enable the detection of mag, we generated a polyclonal rabbit antiserum by immunizing rabbits with His-tagged mag, which was expressed in HEK293 cells and subsequently purified from conditioned medium (Figure S1A). The resulting serum recognized mag with high specificity and sensitivity (Figure S1B). αLNNd was detected using commercial antibodies raised against the N-terminal domain of human laminin-α1. As laminin-α1 is not expressed in mouse skeletal muscle,[32] laminin-α1 antibodies were used for immunohistochemistry and western blot analysis. To test for linker expression, we injected a mixture of

Figure 1. AAV-mediated expression of linker proteins to restore laminin receptor anchorage and polymerization

(A) Schematic representation of laminin heterotrimers containing laminin-α2 (laminin-2X1 [α2, β1/2, γ1]) or laminin-α4 (laminin-4X1 [α4, β1/2, γ1]), and the linker proteins mag and αLNNd in the muscle basement membrane. (B) Domain structure of the linker proteins: mag is a fusion protein comprising the N-terminal and C-terminal regions of agrin, which includes the domains mediating binding to laminins and α-dystroglycan. αLNNd is a fusion protein combining the LN domain and LE modules of laminin-α1 (conferring polymerization activity) with the C-terminal domains of nidogen-1 (providing laminin-γ1 and collagen IV binding sites). (C) Experimental design for (D)–(G): C57BL/6 mice were injected intravenously at postnatal day 1 (P1) with the indicated combination of AAV vectors. Muscle tissue was analyzed at 8 weeks of age. (D and E) Western blot analysis and quantifications (bottom) of αLNNd and mag in lysates from various muscles (D) or triceps muscle (E) of mice injected with AAV9-Spc-αLNNd and AAV9-Spc-mag (referred to as AAV9-Spc-DL). Each mouse received 1.5×10^{14} vg/kg per construct (D) or the indicated dose (E). Glyceraldehyde 3-phosphate dehydrogenase (GAPDH) was used as loading control. (F) Immunofluorescence images of triceps cross-sections stained for αLNNd and mag from mice injected with AAV9-Spc-DL at the indicated doses. (G) Western blot analysis to detect αLNNd and mag in lysates from triceps of mice injected with AAV9-Spc-DL or AAVMYO-Spc-DL at the indicated doses. Relative amounts of αLNNd and mag were normalized to the expression of their respective linker at the highest AAV9 dose. Data are presented as mean ± SEM. *$p < 0.05$ and **$p < 0.01$ by one-way ANOVA with Bonferroni post hoc test. Scale bar: 100 μm. n = 2–3 mice per group.

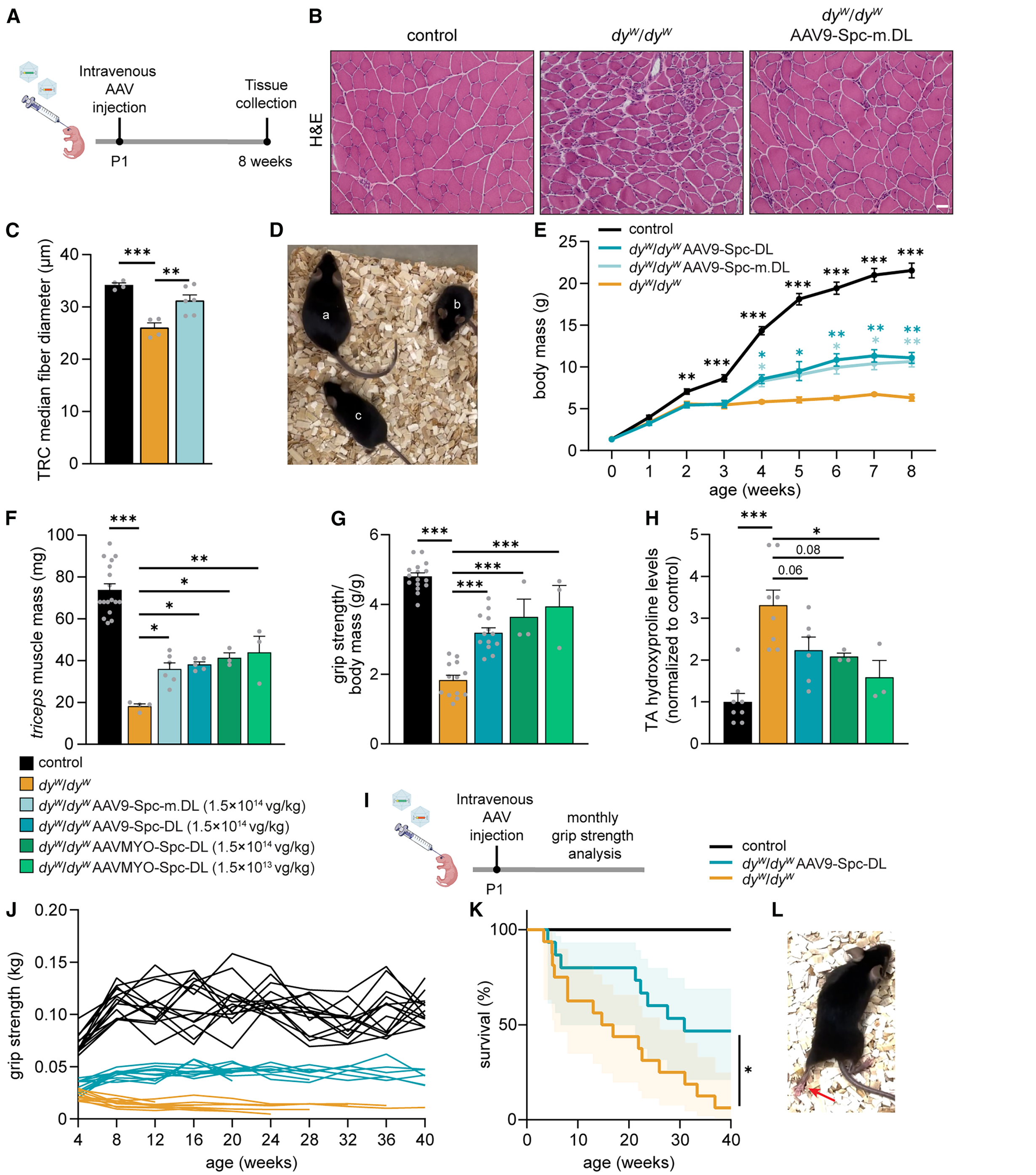

Figure 2. AAV-mediated, muscle-targeted expression of linker proteins for amelioration of muscular dystrophy in dy^W/dy^W mice

(A) Experimental design for (B)–(H): dy^W/dy^W mice were injected intravenously at P1 with the indicated combination of AAV vectors. Muscle tissue was analyzed at 8 weeks of age. (B and C) Histological assessment of triceps muscles from non-injected healthy control, non-treated dy^W/dy^W mice, or dy^W/dy^W mice treated with AAV9-Spc-m.αLNNd and AAV9-Spc-m.mag (referred to as AAV9-Spc-m.DL) at a dose of 1.5×10^{14} vg/kg per construct. (B) Representative images of H&E-stained triceps cross-sections. (C) Quantification of median fiber diameters in triceps cross-sections. (D) Representative picture of a 5-week-old healthy wild-type (a) untreated dy^W/dy^W mouse (b) and a treated dy^W/dy^W AAV9-m.Spc-DL mouse (c). (E) Body mass curves of non-injected healthy control and dy^W/dy^W mice, as well as dy^W/dy^W mice treated with AAV9-Spc-m.DL or the corresponding human linker constructs (AAV9-Spc-DL). (F) Triceps muscle mass at 8 weeks of age of the different groups. (G) Forelimb grip strength normalized to body mass of the different groups. (H) Quantification of muscle fibrosis based on hydroxyproline content measured by amino acid analysis in tibialis anterior (TA) lysates.

(legend continued on next page)

AAV9-Spc-αLNNd and AAV9-Spc-mag, hereafter referred to as AAV9-Spc-DL (DL for double linker), into the temporal vein of wild-type mice at postnatal day 1 (P1) and determined the protein amount in tissues at 8 weeks (experimental scheme in Figure 1C). Each mouse received a dose of 1.5×10^{14} vector genomes/kilogram (vg/kg) per construct. Analysis of multiple muscles and the liver showed high transduction efficiency, as indicated by the quantification of vg per mouse genome (Figure S1C) and of the amount of the linker proteins in various muscles (Figure 1D). To evaluate dose-dependent expression, we lowered the AAV dose up to 4-fold (Figures 1E and 1F). Reducing the dose by half only slightly decreased the linker protein amount and basement membrane localization remained homogenous, suggesting that injection of 7.5×10^{13} vg/kg is still sufficient to saturate muscles with linker proteins. Additional halving of the dose led to a clear decrease in the signal in western blots (Figure 1E) and to non-homogenous appearance in immunohistochemistry, particularly for αLNNd (Figure 1F).

Next, we assessed whether the myotropic AAV variant AAVMYO[33] would increase muscle delivery of the linker proteins. At a dose of 5×10^{13} vg/kg AAVMYO, the amount of mag and αLNNd was not significantly different from what was achieved with AAV9 at 1.5×10^{14} vg/kg (Figure 1G). At a dose of 1.5×10^{13} vg/kg, AAVMYO-delivered linker proteins were still at 74% (mag) and 62% (αLNNd) of the levels observed at maximal dose (Figure 1G). Interestingly, the maximal amounts detected with AAVMYO were not higher than those achieved with 1.5×10^{14} vg/kg AAV9 (Figure 1G), suggesting that we reach saturation at the highest doses irrespective of the delivery AAV capsid.

Since the therapeutic effects of mouse-derived linker proteins have previously been evaluated in LAMA2 MD mouse models through transgenic expression,[21,34] we aimed to assess the efficacy of AAV-mediated delivery of the corresponding mouse linker protein sequences. To this end, we injected dy^W/dy^W mice at P1 with a high, saturating dose of 1.5×10^{14} vg/kg AAV9 carrying the coding sequence for the mouse versions of the linker proteins (AAV9-Spc-m.DL). Treatment effects were assessed until 8 weeks of age (Figure 2A). At this age, dy^W/dy^W mice present with a very severe disease phenotype, but approximately 60% of the untreated mice still survive.[21] In this way, we wanted to ensure that we did not particularly select for long survivors in the non-treated cohort. As shown previously,[21] the absence of laminin-α2 in dy^W/dy^W mice leads to the deposition of laminin-α4, normally restricted to vascular basement membrane, into the muscle fiber basement membrane (Figure S2A). In dy^W/dy^W mice injected with AAV9-Spc-m.DL, both linkers colocalized with laminin-α4 (Figure S2B). Histological analysis (hematoxylin and eosin [H&E] stainings) of muscle cross-sections from untreated dy^W/dy^W mice revealed characteristic features of severe muscular dystrophy, including pronounced fiber size variation, extensive fibrosis, mononuclear cell infiltration, and presence of centralized myonuclei in both triceps (Figure 2B) and diaphragm muscle (Figure S3A). In contrast, muscle histology was markedly improved in AAV9-Spc-m.DL-treated dy^W/dy^W mice. Notably, the median triceps muscle fiber diameter was restored to control levels (Figure 2C), and diaphragm thickness was significantly higher than in untreated mice (Figure S3A). The tibialis anterior (TA) muscle also increased in size and exhibited histological improvement (Figure S3B), although its median fiber diameter did not change significantly (Figure S3B). In line with the histological improvements, treated mice displayed visibly enhanced body size and overall appearance (Figure 2D; Video S1). These findings demonstrate that AAV-mediated expression of mouse linker proteins achieves therapeutic efficacy comparable to that of transgenic expression.

The amino acid sequence of the linker proteins is highly conserved between mouse and human, reaching 84% similarity for mag and 87% similarity for αLNNd. To determine whether the human linker proteins are functionally equivalent to their mouse homologs, we conducted a direct comparison. Mice injected at P1 with AAV9 vectors encoding either the mouse (m.DL) or the human (DL) versions of the linker proteins showed comparable weight gain starting 3 weeks post-injection (Figure 2E), ultimately reaching approximately a 2-fold increase in body weight by 8 weeks of age compared with untreated dy^W/dy^W mice. Similarly, the mass of various skeletal muscles was comparably increased in both treated groups (Figure S3C). These results provide strong evidence that the human versions are as effective as their mouse homologs. Given the necessity of using human sequences for future clinical translation, all subsequent experiments were conducted using the human linker proteins.

To determine whether the efficiency achieved with the maximal dose of AAV9 (1.5×10^{14} vg/kg) could be further enhanced, we next evaluated the use of AAVMYO for linker protein delivery. To assess both its potential superiority and potency, we injected dy^W/dy^W mice with AAVMYO-Spc-DL at the maximal dose (1.5×10^{14} vg/kg) or a 10-fold lower dose. Consistent with protein expression levels detected by western blot analysis across the dose range, both triceps muscle mass and forelimb grip strength at 8 weeks of age showed significant improvement in treated dy^W/dy^W mice (Figures 2F and 2G). Fibrosis, assessed by hydroxyproline quantification, was likewise reduced in the TA muscle (Figure 2H). These improvements were comparable to those observed with high-dose AAV9 and both

(I) Experimental design for (J)–(L): dy^W/dy^W mice were injected intravenously at P1 with AAV9-Spc-DL (1.5×10^{14} vg/kg per construct) and evaluated monthly for grip strength. (J) Longitudinal assessment of individual forelimb grip strength. Discontinuous lines indicate that the mouse died during the course of the study. (K) Survival curves of 15 mice per group (8 males and 7 females). Survival was significantly improved in treated animals (*$p = 0.017$ by log rank Mantel-Cox test). Shaded areas indicate the 95% confidence interval. (L) Representative photograph of an 8-month-old dy^W/dy^W treated with AAV9-Spc-DL at P1. Note the hindlimb paralysis (red arrow). Data are presented as mean ± SEM. *$p < 0.05$, **$p < 0.01$, ***$p < 0.001$ by one-way ANOVA with Dunnett's post hoc test, comparing each group to untreated dy^W/dy^W mice. Scale bar: 50 μm. n = 3–17 mice per group.

high- and low-dose AAVMYO (Figures 2F–2H). These results indicate that similar to what was previously described,[33] the use of AAVMYO would allow a substantial dose reduction compared with AAV9, while maintaining sufficient expression to achieve maximal therapeutic effects in muscle.

To assess the long-term durability of the treatment, we performed monthly forelimb grip strength measurements (Figure 2I). Treated dy^W/dy^W mice exhibited increased strength beginning at 4 weeks of age, and the grip strength remained stable up to the age of 40 weeks (Figure 2J). Note that from the cohort of 15 mice, only 1 untreated dy^W/dy^W mouse survived until 40 weeks of age, while 7 of the treated mice survived past 40 weeks (Figure 2K). The sustained therapeutic effect of AAV-mediated linker protein expression was further supported by analysis of the triceps muscle from a treated dy^W/dy^W mouse that survived for 2 years. Even at this advanced age, histological improvements were evident, including increased myofiber size and a reduced infiltration of mononuclear cells (Figure S4A). Moreover, the basement membrane surrounding all muscle fibers remained positive for mag (Figure S4B). Similar to the pattern observed at the low dose (Figure 1F), αLNNd expression appeared more mosaic, potentially due to the larger size of the construct or the lower affinity of the detection antibody (Figure S4B).

αLNNd, but not mag, ameliorates peripheral neuropathy in dy^W/dy^W mice

While body weight, muscle histology, and function were significantly improved, the use of muscle-specific promoters to drive linker protein expression restricted the therapeutic effect to muscle and unmasked a progressive peripheral neuropathy that ultimately led to complete hindlimb paralysis (Figure 2L; Video S2), which subsequently may have limited further improvements in body weight, strength, and survival. In all laminin-α2-deficient mouse models, peripheral neuropathy predominantly affects the hindlimbs and is characterized by defects in axonal sorting and myelination.[12] Notably, skeletal muscle-specific expression of *Lama2* also fails to correct the peripheral neuropathy in dy^W/dy^W mice.[35] In contrast, ubiquitous transgenic expression of both linker proteins prevents hindlimb paralysis in dy^W/dy^W and dy^{3K}/dy^{3K} mice.[34] Hence, the linker proteins are also effective in improving peripheral nerve pathology when expressed ubiquitously.

To determine whether both linker proteins are required to prevent hindlimb paralysis, we analyzed dy^W/dy^W mice transgenically expressing only one linker protein under the control of the ubiquitous cytomegalovirus (CMV) enhancer/chicken β-actin promoter (CAG) promoter. Strikingly, dy^W/dy^W mice expressing solely the αLNNd linker protein (dy^W/dy^W CAG-αLNNd) did not develop hindlimb paralysis (Figure 3A; Video S3), whereas those expressing only mag (dy^W/dy^W CAG-mag) showed no improvement (Figure 3B; Video S4). Locomotor function analysis of 8-week-old mice confirmed that dy^W/dy^W CAG-αLNNd mice, but not dy^W/dy^W CAG-mag mice, achieved a near-normal gait speed compared with dy^W/dy^W mice (Figure 3C). Consistent with these functional improvements, sciatic nerve histology revealed normal axonal sorting and myelination in dy^W/dy^W CAG-αLNNd mice, whereas dy^W/dy^W mice showed large bundles or islands of "naked" axons containing many large-caliber axons (Figure S5A).

In prior experiments, we observed that transgenic muscle-specific expression of the two linker proteins could not fully restore hindlimb muscle weight in dy^W/dy^W mice.[21] Similarly, AAV-mediated muscle-specific expression reduced fibrosis in the TA (Figure 2H) but did not significantly increase median muscle fiber diameter (Figure S3B), in contrast to the triceps (Figure 2C). This discrepancy is likely due to secondary muscle atrophy caused by the peripheral neuropathy. To address this, we analyzed TA muscles from dy^W/dy^W CAG-αLNNd and dy^W/dy^W CAG-mag mice. While muscle mass was restored in dy^W/dy^W CAG-DL (double transgenic for both linkers) and dy^W/dy^W CAG-αLNNd mice, dy^W/dy^W CAG-mag mice retained the characteristic atrophy-linked muscle mass reduction (Figure 3D). Partial histological improvements could be observed in TA muscle with either linker protein alone (Figure 3E), consistent with prior muscle-specific transgenic studies.[21] Similarly, expression of both linker proteins was required to restore fiber size distribution comparable to control female mice (Figure 3F), while αLNNd expression alone reduced the proportion of small fibers but increased the number of exceptionally large fibers. Importantly, significant reduction in fiber size variability also required the presence of both linker proteins, as neither linker alone improved this parameter (Figure 3G). Assessment of fibrosis, determined by the collagen area in Sirius Red staining, showed a decrease with either linker protein, with the greatest reduction observed again when both linkers were combined (Figure 3H). Collectively, and in agreement with previous findings, these results demonstrate the additive benefit of the two linker proteins on muscle histology,[21] whereas αLNNd alone is sufficient to ameliorate the peripheral neuropathy.

To explore the mechanistic basis for this differential effect, we assessed laminin-α4 expression in peripheral nerves. In wild-type mice, laminin-α4 localizes primarily to the perineurium, whereas laminin-α2 is the predominant laminin α-chain in the endoneurium.[34,36] In dy^W/dy^W mice, laminin-α4 was also detected in the endoneurium (Figure S5B).[34] Strikingly, laminin-α4 staining was markedly increased in the endoneurium of dy^W/dy^W CAG-αLNNd mice but remained unchanged in dy^W/dy^W CAG-mag mice (Figure S5B). Western blot analysis corroborated this finding, confirming elevated laminin-α4 levels in dy^W/dy^W CAG-αLNNd mice (Figure S5C). These findings demonstrate that αLNNd sequesters laminin-4X1 within the endoneurial basement membrane. Given that Schwann cells, in contrast to muscle fibers, express the laminin-4X1 receptor integrin-α6β1,[37,38] the linkage of laminin-4X1 to α-dystroglycan via mag may not be essential in peripheral nerves. Instead, the ability of αLNNd to polymerize laminin-4X1 may enable a proper basement membrane formation and allow its engagement with integrin-α6β1, providing a mechanistic explanation for the functional restoration observed in dy^W/dy^W CAG-αLNNd mice.

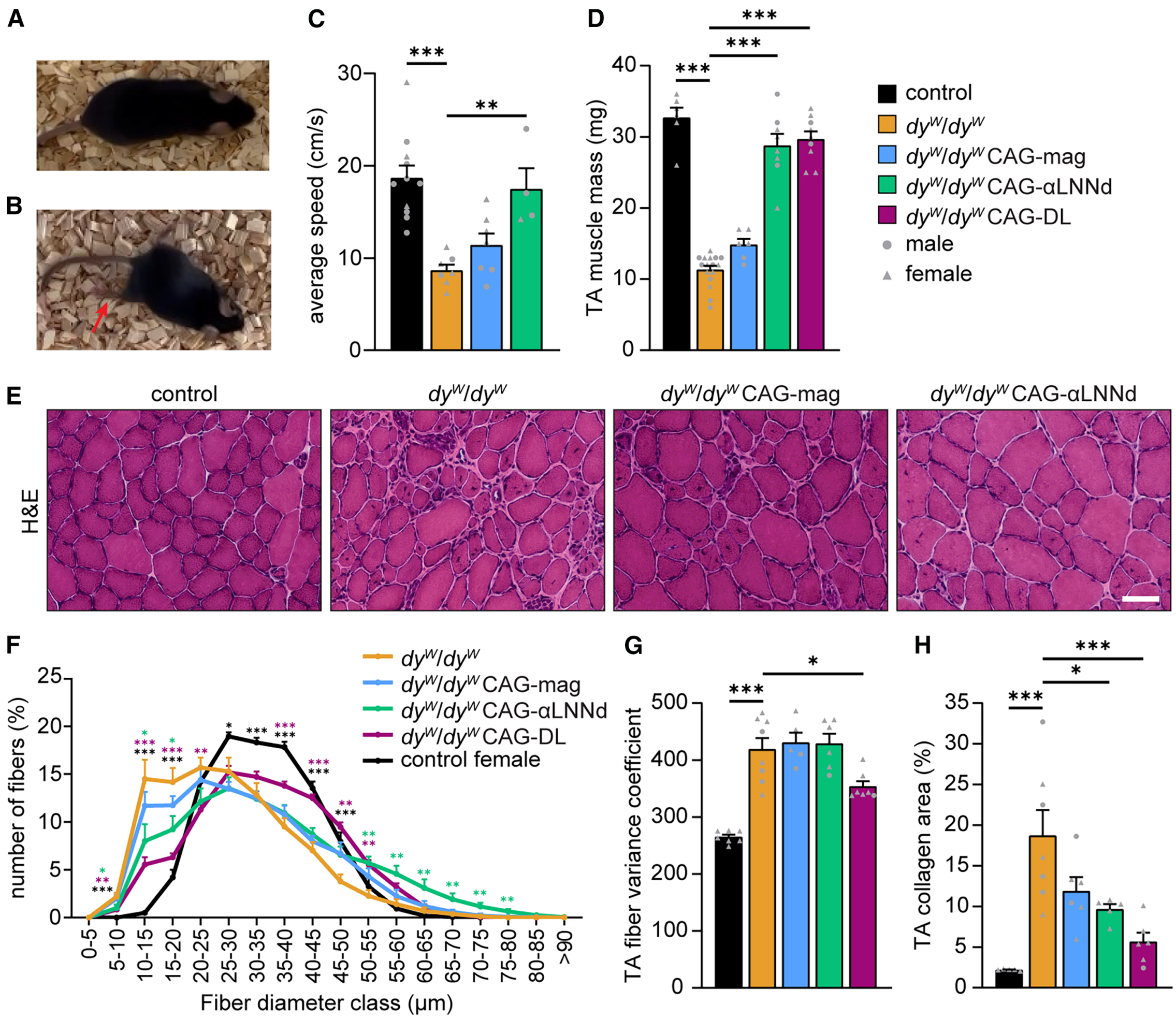

Figure 3. Effect of ubiquitous transgenic expression of αLNNd and mag on peripheral neuropathy and muscular dystrophy in dy^W/dy^W mice

(A) Representative photograph of an 8-month-old dy^W/dy^W CAG-αLNNd transgenic mouse. (B) Representative photograph of a 2-month-old dy^W/dy^W CAG-mag transgenic mouse showing signs of peripheral neuropathy (hindlimb dragging; red arrow). (C) Quantitative analysis of locomotor speed in 8-week-old mice using a gait analysis system. Groups include healthy controls, dy^W/dy^W mice, and dy^W/dy^W transgenic mice ubiquitously expressing mag or αLNNd under the control of the CAG promoter (CAG-mag or CAG-αLNNd). (D) TA muscle mass at 8 weeks of age in control mice, dy^W/dy^W mice, and dy^W/dy^W transgenic mice expressing single linker proteins or both in combination (CAG-DL). (E–H) Histological analysis of TA muscles from 8-week-old controls, dy^W/dy^W mice, and dy^W/dy^W transgenic mice as indicated. (E) Representative images of H&E-stained TA cross-sections. (F) Distribution of muscle fiber diameters in TA cross-sections. (G) Variance coefficient of TA fiber diameters. (H) Quantification of muscle fibrosis based on Sirius red$^+$ area in TA cross-sections. Data are presented as mean ± SEM. *$p < 0.05$, **$p < 0.01$, and ***$p < 0.001$ by one-way ANOVA with Dunnett's post hoc test, comparing each group to dy^W/dy^W mice. Scale bar: 100 μm. n = 4–11 mice per group.

Shortening of αLNNd for efficient AAV9-mediated expression in muscle and nerve

To enable AAV-mediated ubiquitous expression of αLNNd, we engineered a truncated variant, αLNNdΔG2, by removing the nidogen-G2 domain and two adjacent epidermal growth factor-like domains (Figure 4A). This truncation preserves laminin polymerization activity[39] while reducing the coding sequence to comply with AAV packaging limits when using a larger promoter. To dissect the influence of the promoter and the construct size to expression levels, we generated AAV constructs encoding full-length αLNNd or αLNNdΔG2, driven by either the muscle-specific Spc5.12 promoter or the ubiquitous CBh promoter.[40] Protein expression was evaluated 4 weeks post-injection (Figure 4B). Western blot analysis of TA muscle lysates showed higher αLNNdΔG2 levels compared with αLNNd when driven by the Spc5.12 promoter, with expression levels further increased by the CBh promoter (Figures 4C and 4D). To account for potential size-dependent transfer efficiency differences in western blot analysis, we performed liquid chromatography-mass spectrometry (LC-MS) using peptides shared by both constructs. LC-MS confirmed a similar expression pattern, albeit with attenuated absolute fold changes compared to western blot data (Figure 4E). The increase in protein amount also correlated with elevated transcript levels (Figure 4F). Most important, CBh-driven αLNNdΔG2, unlike the Spc5.12-driven construct, localized

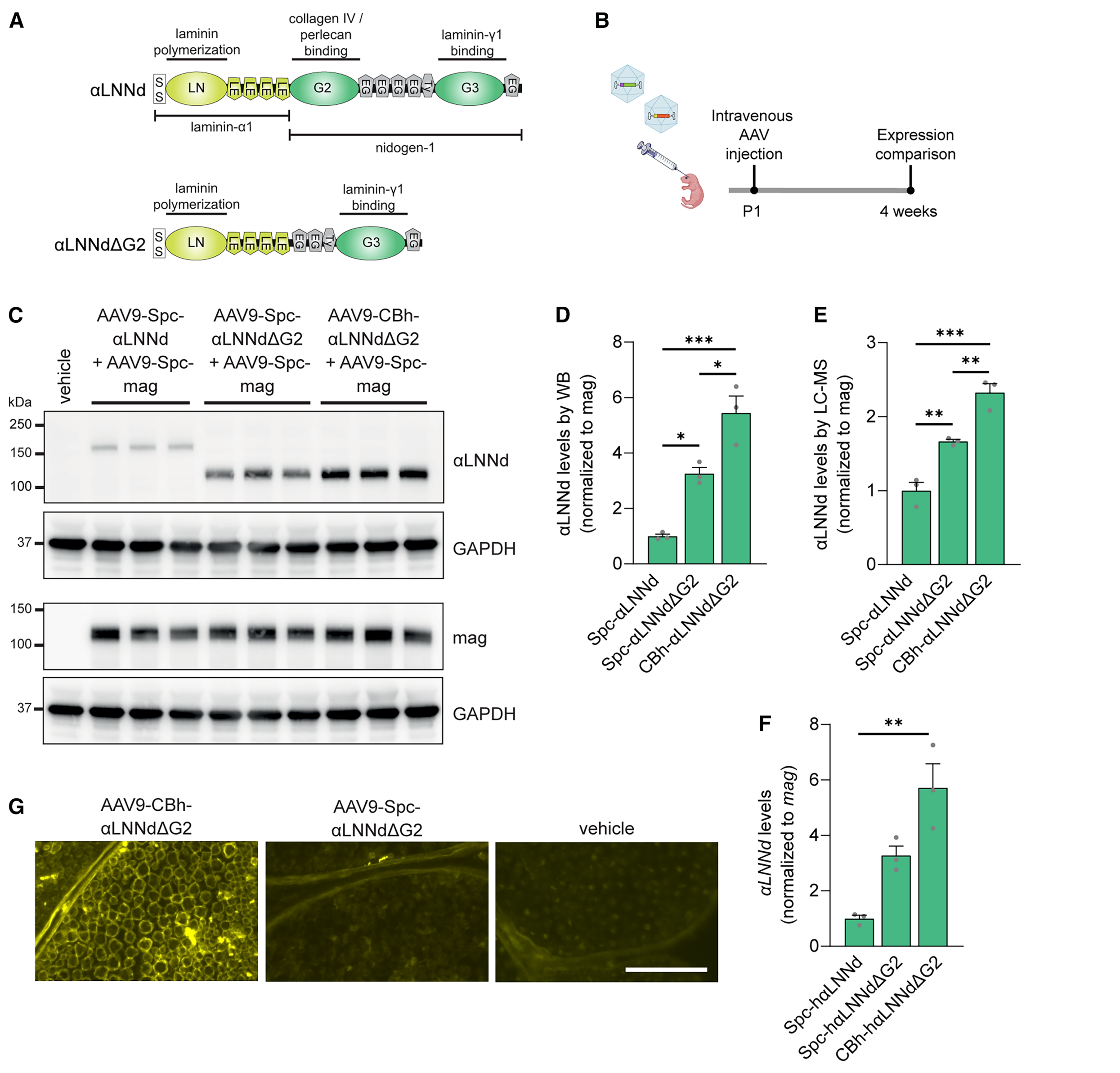

Figure 4. Shortening of αLNNd to allow CBh promoter-driven expression in peripheral nerve

(A) Schematic representation of the protein domains present in full-length αLNNd and its shortened version αLNNdΔG2. (B) Experimental design for (C)–(F): C57BL/6 mice were injected intravenously at P1 with PBS (vehicle) or with the indicated combination of AAV vectors (1.5×10^{14} vg/kg per construct). Tissue was analyzed at 4 weeks of age. (C–E) Analysis of αLNNd, αLNNdΔG2, and mag protein amounts in triceps muscle lysates. (C) Western blot detection of mag, αLNNd, or αLNNdΔG2. Antibodies against the LN domain of laminin-α1 were used to detect both αLNNd variants. GAPDH served as a loading control. (D) Quantification of protein levels from western blot analysis. (E) Quantification of protein abundance by LC-MS. (F) Quantification of transcript levels by qPCR. (G) Immunofluorescence images of sciatic nerve cross-sections stained for αLNNdΔG2 using antibodies against the LN domain of laminin-α1. Data are presented as mean ± SEM. *$p < 0.05$, **$p < 0.01$, and ***$p < 0.001$ by one-way ANOVA with Bonferroni's post hoc test. $n = 3$ mice per group.

to the basement membrane of the sciatic nerve, including both the peri- and endoneurium (Figure 4G).

Given that the myotropic AAVMYO achieves higher expression of the linker proteins in skeletal muscle than AAV9 at several-fold lower doses (Figure 1G), we evaluated its potential to target peripheral nerves. At the dose of 1.5×10^{13} vg/kg, αLNNdΔG2 could not be detected in the endoneurium of the peripheral nerve (Figure S6A). We next tested AAVMYO2, another myotropic capsid generated through shuffling and peptide display, that additionally de-targets the liver.[41] Even at a 10-fold higher dose (1.5×10^{14} vg/kg), AAVMYO2-delivered αLNNdΔG2 protein could not be detected

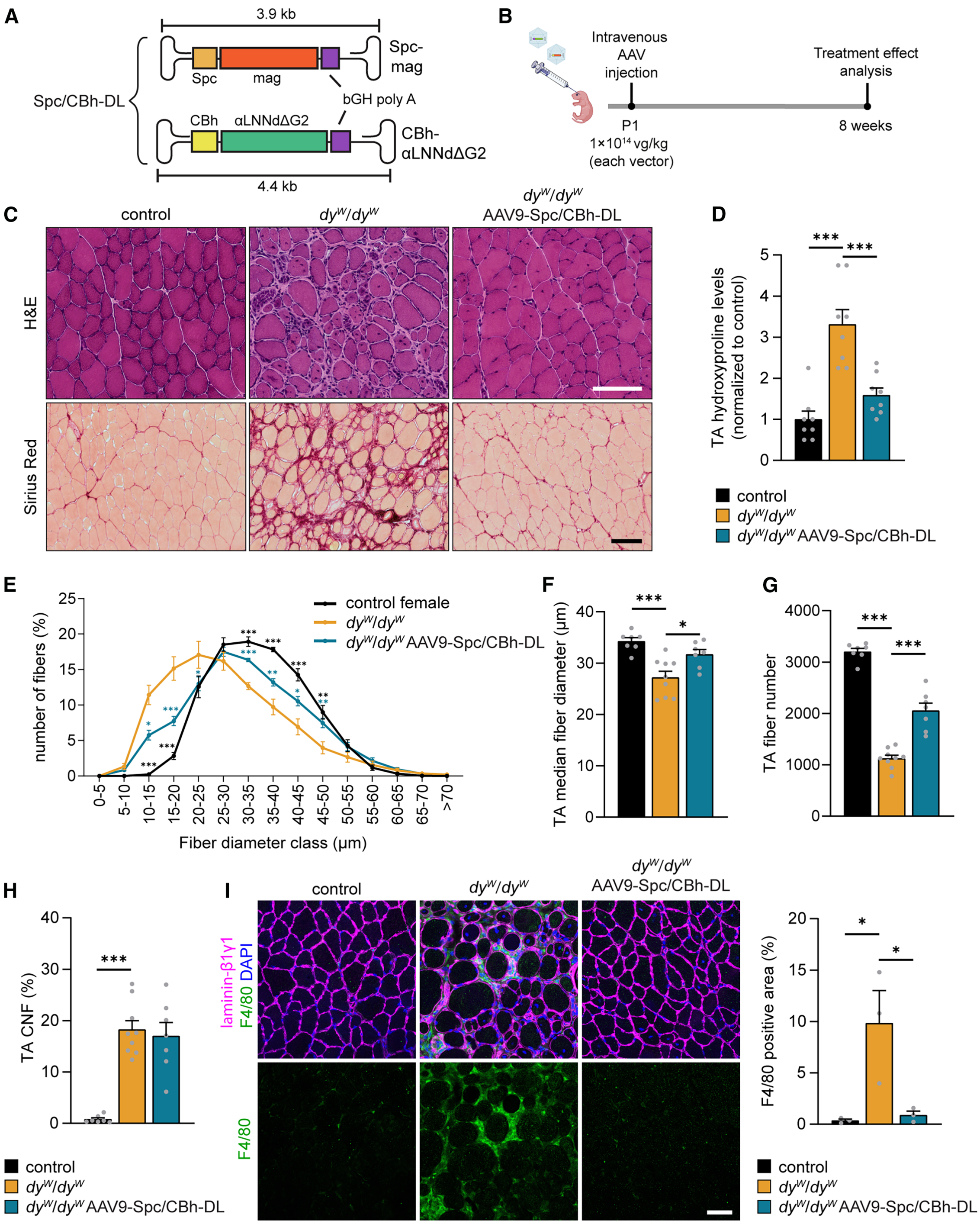

Figure 5. Hindlimb muscle histology upon AAV-mediated ubiquitous expression of αLNNdΔG2 combined with muscle-specific expression of mag in dy^W/dy^W mice

(A) Schematic illustration of vector constructs used in the Spc/CBh-DL treatment. Expression of mag is driven by the muscle-specific Spc promoter, while αLNNdΔG2 is expressed under the control of the ubiquitous CBh promoter. Polyadenylation signal is derived from bovine growth hormone (BGH). Size of the constructs are shown in kilobases (kb). (B) Experimental design for data shown in this figure and Figure 6. dy^W/dy^W mice were injected intravenously at P1 with a mixture of AAV9 vectors: one packaged with Spc-mag and the other packaged with CBh-αLNNdΔG2. This combination is referred to as AAV9-Spc/CBh-DL. Each construct was administered at a dose of 1×10^{14} vg/kg. Treatment effects were analyzed at 8 weeks of age. (C) Representative images of H&E- and Sirius red-stained TA muscle cross-sections from non-injected

(legend continued on next page)

in the peripheral nerve (Figure S6B). As expected, AAVMYO2 clearly outperformed AAV9 in skeletal muscle at lower doses (Figures S6C–S6E).

In summary, αLNNdΔG2 delivered by AAV9 at a dose of 1.5×10^{14} vg/kg using the CBh promoter localizes to both the muscle and peripheral nerve basement membrane, suggesting that it may ameliorate pathology in both tissues following AAV administration. Furthermore, this construct showed markedly improved expression in muscle compared with Spc-αLNNd, potentially improving durability and enabling therapeutic efficacy at lower doses. We also concluded that AAVMYO and AAVMYO2 are not suitable vectors to deliver αLNNdΔG2 to the peripheral nerve and therefore excluded them from further evaluation.

Early treatment of dy^W/dy^W mice with ubiquitously expressed αLNNdΔG2 and muscle-specific mag reduces all disease hallmarks

Having established that αLNNdΔG2 is efficiently expressed in peripheral nerves using AAV9 and prevents the peripheral neuropathy when expressed transgenically, we next tested its therapeutic efficiency when delivered to dy^W/dy^W mice via AAV9. To this end, we combined AAV9 vectors encoding mag under the control of the Spc5.12 promoter (Spc-mag; Figure 5A) with an equal amount of AAV9 encoding αLNNdΔG2 driven by the CBh promoter (CBh-αLNNdΔG2; Figure 5A). The AAV9 mixture (referred to as AAV9-Spc/CBh-DL) was then intravenously administered to dy^W/dy^W mice at P1 at a dose of 1×10^{14} vg/kg per construct, and tissues were analyzed at 8 weeks of age (Figure 5B). Histological analysis of TA muscle revealed a strong reduction in the dystrophic hallmarks (Figures 5C and S7A), including decreased fibrosis as demonstrated by Sirius red staining (Figure 5C). This improvement in fibrosis was further confirmed by highly significant reduction in collagen content, measured by hydroxyproline quantification (Figure 5D). Muscle fiber size distribution shifted markedly toward larger fibers (Figure 5E), accompanied by increases in median fiber diameter (Figure 5F) and fiber number (Figure 5G). Despite these improvements, a high proportion of fibers retained centralized nuclei (Figure 5H). TA muscle of treated dy^W/dy^W mice also showed little to no signs of inflammation as determined by staining using antibodies against the macrophage marker F4/80 (Figure 5I). Quantitative assessment of the disease amelioration by the linker proteins was very similar in triceps muscle (Figures S7B–S7E). In addition, histological improvements were observed in respiratory muscles, including the diaphragm and intercostal muscles, following AAV9-Spc/CBh-DL treatment (Figure S7F). Notably, αLNNdΔG2 displayed more homogeneous expression in the diaphragm of treated mice compared with αLNNd (Figure S7G), which may contribute to improved and more sustained respiratory function and, consequently, enhanced survival following AAV9-Spc/CBh-DL treatment relative to AAV9-Spc-DL treatment.

To assess the transcriptional effects of the treatment, we performed bulk RNA sequencing (RNA-seq) on TA muscle from 8-week-old mice. We included six wild-type controls, six untreated dy^W/dy^W mice, and six dy^W/dy^W mice treated with AAV9-Spc/CBh-DL mice (three males and three females per group). In addition, we included three healthy control mice injected with AAV9-Spc/CBh-DL to evaluate potential off-target effects of the treatment. Unbiased sample correlation analysis revealed a clear separation between untreated dy^W/dy^W mice and treated dy^W/dy^W mice, while wild-type and AAV9-Spc/CBh-DL-injected wild-type mice were intermixed (Figure S8A). This indicates a strong transcriptional effect of the linker protein treatment in dy^W/dy^W muscle. Importantly, AAV9-Spc/CBh-DL-injected healthy control mice showed only minimal transcriptional changes (Figure S8B). We next compared the transcriptome of untreated dy^W/dy^W mice with untreated wild-type mice using a threshold for differentially expressed genes of log2 ± 0.5 and an adjusted $p \leq 0.05$. Out of the 14,905 detected transcripts, 14% were significantly downregulated and 18% were significantly upregulated (Figure 6A). Among the most significantly upregulated genes were *Sln* and *Cilp*, encoding sarcolipin and cartilage intermediate layer protein, respectively, both known markers of dystrophic pathology.[42,43] Consistent with the strong ameliorative effect of the two linker proteins on the pathology in dy^W/dy^W mice, expression of the dysregulated genes was changed toward wild-type in dy^W/dy^W AAV9-Spc/CBh-DL mice (Figure 6B), with their transcriptome being closer to wild-type controls than untreated dy^W/dy^W mice (Figure S8A). This "normalization" of the gene expression profiles was clearly confirmed by the very strong correlation ($R^2 > 0.6$) between genes changed in dy^W/dy^W mice (log2 fold change (dy^W/dy^W vs. wild-type) and genes changed in dy^W/dy^W mice after treatment (log2 fold change (dy^W/dy^W AAV9-Spc/CBh-DL vs. dy^W/dy^W) (Figure S8C).

Gene set enrichment analysis[44] (MSigDB[38]) identified hallmark pathways changed in dy^W/dy^W muscle, including increases in transcripts involved in fibrosis (epithelial-mesenchymal transition, tumor necrosis factor-α signaling), inflammation, and apoptosis, alongside downregulated transcripts associated with oxidative phosphorylation, fatty acid metabolism, and adipogenesis. Notably, all hallmark pathways responded positively to treatment (Figure 6C). Fibrosis and inflammation markers (*Postn* and *Cd68*), previously shown to be highly upregulated in laminin-α2 deficiency,[45–47] were

healthy controls, untreated dy^W/dy^W mice, and dy^W/dy^W mice treated with AAV9-Spc/CBh-DL. (D) Quantification of muscle fibrosis based on hydroxyproline content measured by amino acid analysis in TA lysates. (E–H) Quantitative analysis of TA muscle histology in non-injected healthy controls, untreated dy^W/dy^W mice, and dy^W/dy^W mice treated with AAV9-Spc/CBh-DL. (E) Distribution of muscle fiber diameters. (F) Median muscle fiber diameters. (G) Total muscle fiber number. (H) Percentage of fibers with centralized nuclei. (I) Immunofluorescence images of TA cross-sections stained for laminins (β1γ1 chains) and F4/80 (macrophage marker), along with the quantification of the F4/80$^+$ area. Data are presented as mean ± SEM. *$p < 0.05$, **$p < 0.01$, and ***$p < 0.001$ by one-way ANOVA with Dunnett's post hoc test, comparing each group to untreated dy^W/dy^W mice. Scale bar: 100 μm. n = 3–9 mice per group.

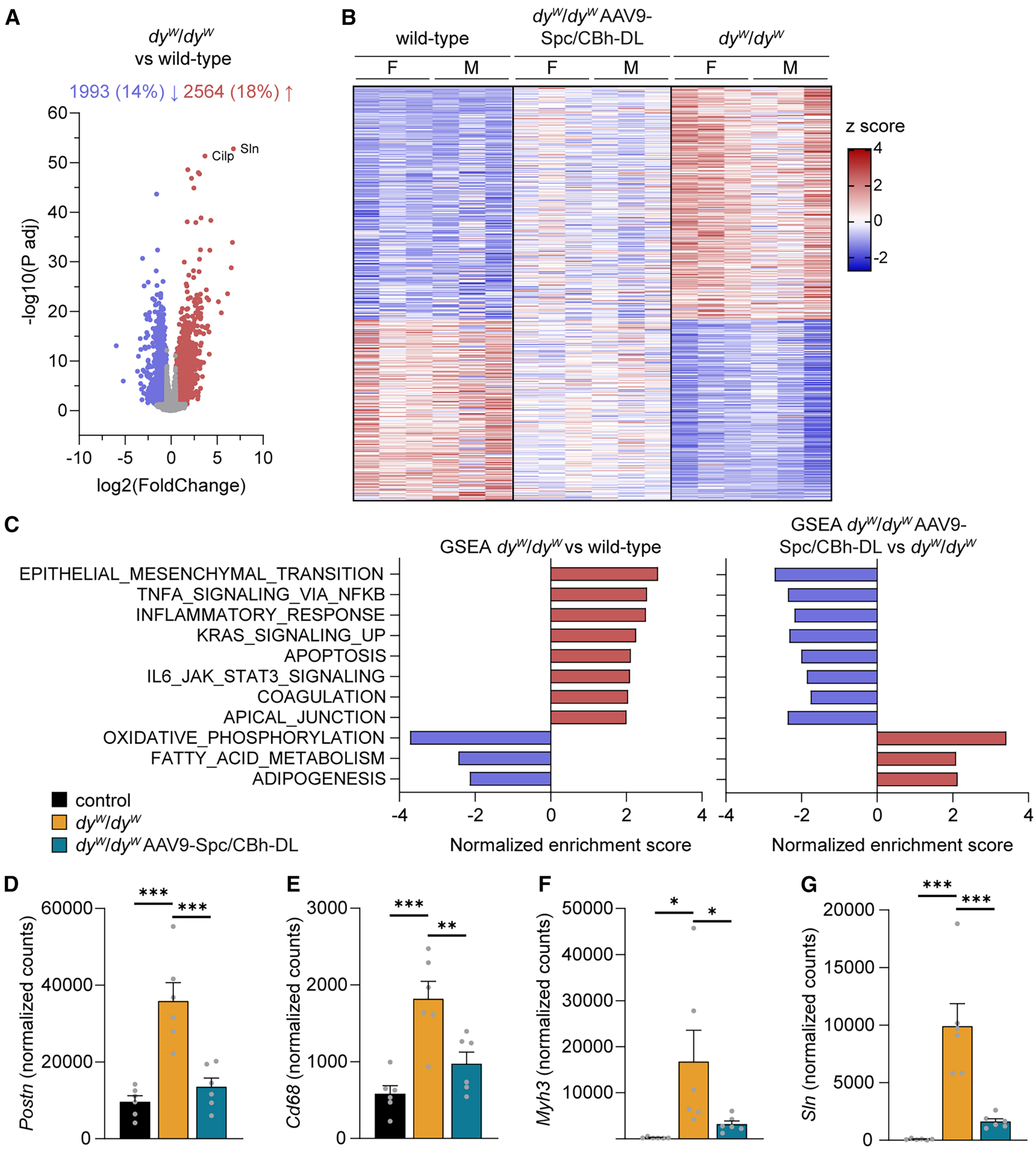

Figure 6. Normalization of dysregulated transcriptional hallmarks by ubiquitous expression of αLNNdΔG2 in combination with muscle-specific expression of mag

(A–G) RNA-seq analysis of TA muscle from 8-week-old wild-type, *dy*^{W}/*dy*^{W}, and *dy*^{W}/*dy*^{W} mice injected intravenously at P1 with AAV9-Spc/CBh-DL (1×10^{14} vg/kg per construct). (A) Differentially expressed transcripts in *dy*^{W}/*dy*^{W} versus wild-type mice presented as a volcano plot. Differentially expressed genes (DEGs) are shown in red (upregulated) or blue (downregulated), based on a threshold of log2 fold change > ±0.5 and adjusted $p < 0.05$. Numbers on top indicate the number and percentage of upregulated (red) or downregulated (blue) transcripts. (B) Heatmap of *Z* scores for the 4,557 DEGs in *dy*^{W}/*dy*^{W} versus wild-type mice, and corresponding *Z* scores in *dy*^{W}/*dy*^{W} AAV9-Spc/CBh-DL mice. (C) (Left) Gene set enrichment analysis (GSEA) of Hallmark Gene Set (MSigDB) comparing *dy*^{W}/*dy*^{W} and wild-type muscle. Gene sets with a normalized enrichment score (NES) > ±2 are shown. (Right) NES values for the same gene sets in *dy*^{W}/*dy*^{W} AAV9-Spc/CBh-DL versus untreated *dy*^{W}/*dy*^{W} mice. (D–G) Normalized transcript counts for selected genes: (D) *Postn* (marker of fibrosis), (E) *Cd68* (marker for inflammation), (F) *Myh3* (marker for fiber degeneration/regeneration), and (G) *Sln* (associated with calcium homeostasis). Data are presented as mean ± SEM. *$p < 0.05$, **$p < 0.01$, and ***$p < 0.001$ by one-way ANOVA with Dunnett's post hoc test, comparing each group to untreated *dy*^{W}/*dy*^{W} mice. $n = 6$ mice per group.

significantly reduced post-treatment (Figures 6D and 6E). *Myh3*, an indicator of active muscle regeneration, was also significantly downregulated, suggesting reduced degeneration/regeneration cycles despite persistent central nucleation (Figure 6F). *Sln*, the most strongly dysregulated gene in dy^W/dy^W mice, also responded robustly to treatment (Figure 6G). All in all, these results are further support for the effectiveness of the linker proteins to normalize the disease signature toward wild-type mice.

Functional measures reach near-normal values in dy^W/dy^W mice treated early with AAV9- or AAV8-delivering muscle-expressed mag and ubiquitously expressed αLNNdΔG2

During our studies, we observed that some dy^W/dy^W mice treated at P1 with AAV9-Spc/CBh-DL were nearly indistinguishable from their healthy littermates at 8 weeks of age (Figure 7A; Video S5). To evaluate this observation quantitatively, we subjected the mice to a range of functional assessments. As observed with muscle-specific expression of the linker proteins (Figure 2E), body weight in treated dy^W/dy^W mice remained comparable to that of untreated dy^W/dy^W mice until approximately 3 weeks of age. However, beyond this time point, AAV9-Spc/CBh-DL-injected dy^W/dy^W mice began to gain weight at a similar rate as their control littermates (Figure 7B). At 8 weeks of age, gait speed, a readout of peripheral nerve function, was restored to near-normal levels in treated dy^W/dy^W mice (Figure 7C). Furthermore, when normalized to body mass, muscle mass in AAV9-Spc/CBh-DL-treated dy^W/dy^W mice was very similar to that of healthy controls (Figure S9A).

Given the favorable safety and efficacy profile of AAV8 in recent clinical trials,[48,49] we also tested AAV8 as a delivery vehicle for the linker proteins. To compare targeting efficiency between AAV8 and AAV9, we used a CMV-GFP construct packaged into either serotype. Consistent with previous reports,[50] muscle transduction was comparable between AAV8 and AAV9 (Figure S9B). Importantly, AAV8 also efficiently transduced peripheral nerves (Figure S9C). Similar to AAV9-Spc/CBh-DL, injection of AAV8-Spc/CBh-DL at P1 resulted in homogenous basement membrane coverage in the TA muscle of dy^W/dy^W mice with both mag and αLNNdΔG2 (Figure S10A) and robust αLNNdΔG2 expression in sciatic nerve (Figure S10B). The therapeutic efficacy of AAV8-Spc/CBh-DL mirrored that observed with AAV9, as evidenced by reduction in inflammation (F4/80) and fibrosis (periostin) (Figure S10C) and the near-absence of embryonic myosin heavy-chain-positive ($eMHC^+$) regenerating fibers compared with untreated dy^W/dy^W mice (Figures S10D and S10E). Body mass (Figure 7D) and muscle mass (Figure 7E) at 8 weeks of age were indistinguishable from those achieved with AAV9-mediated delivery. Functional assessments revealed improved grip strength (Figure 7F) and *ex vivo* extensor digitorum longus (EDL) force generation (Figures 7G and 7H), both of which are severely impaired in untreated dy^W/dy^W mice. In line with these functional and weight improvements, muscle histology was similarly improved with AAV8 (Figure S11A), as with AAV9 treatment (Figure 5), reflected by reduced fibrosis (Figure S11B), increased muscle fiber number (Figure S11C), and normalization of fiber size distribution (Figures S11D and S11E). These results demonstrate that neonatal administration of Spc/CBh-DL via either AAV8 or AAV9 restores near-normal function and structure in the muscle and the peripheral nerve of dy^W/dy^W mice by 8 weeks of age.

Late treatment still improves defects in dy^W/dy^W mice

In patients with severe LAMA2 MD, symptoms are present at birth or become evident by 6 months of age.[6] Similarly, dy^W/dy^W mice exhibit an early-onset, severe phenotype that progressively worsens over time. To determine whether the linker treatment can improve the disease at more advanced disease stages, we assessed the treatment effect of the linkers when administered at 3 weeks of age. At this stage, dy^W/dy^W mice show significant muscle impairment, pronounced fibrosis, and clear signs of peripheral neuropathy. This time point also coincides with the onset of increased mortality in untreated dy^W/dy^W mice. To accommodate the higher body weight and slower growth rate of older mice, and to remain within the upper bounds of vector capacity while achieving therapeutic saturation, we intravenously injected 5×10^{13} vg/kg of each vector. This dose aligns with those safely administered in clinical trials for neuromuscular diseases.[48]

To quantitatively assess therapeutic efficacy, grip strength was measured at 8 weeks of age, followed by rotarod performance and tissue collection at 12 weeks (Figure 8A). AAV8-Spc/CBh-DL-treated dy^W/dy^W mice exhibited a markedly improved phenotype, with particularly notable effects on the progression of peripheral neuropathy. Hindlimb dragging, a common feature in untreated 12-week-old dy^W/dy^W mice, was noticeably reduced in treated animals (Figure 8B; Video S6). At 12 weeks of age, robust αLNNdΔG2 expression was detected in sciatic nerve (Figure 8C), and the basement membranes of all muscle fibers stained positive for both mag and αLNNdΔG2 (Figure 8D). Quantification of the percentage of the endoneurial basement membrane that was positive for laminin-α4 and αLNNdΔG2 revealed approximately 50% coverage when AAVs were injected at P1 with 1×10^{14} vg/kg or at 3 weeks with 5×10^{13} vg/kg (Figure S12A). In grip strength analysis, untreated dy^W/dy^W mice that reached 12 weeks of age showed a further decline in muscle function compared with those at 8 weeks; in contrast, treated mice were significantly improved (Figure 8E). Additionally, the severe peripheral neuropathy in untreated mice limited their ability to stay on a rotating rod, with latency to fall of only a few seconds. Treated mice, however, exhibited a significantly increased latency to fall, reflecting enhanced motor coordination and strength (Figure 8F). Histological analysis of the TA muscle of 12-week-old mice revealed clear histological improvement in treated dy^W/dy^W mice (Figure 8G), accompanied by a reduction in inflammatory infiltration (F4/80 staining) (Figure S12B). Notably, fibrosis was strikingly reduced, as evidenced by the significant decrease in collagen-positive area in Sirius red-stained sections (Figure 8H) and diminished $periostin^+$ area (Figures S12B and S12C). Furthermore, the median muscle fiber diameter was increased (Figure 8I),

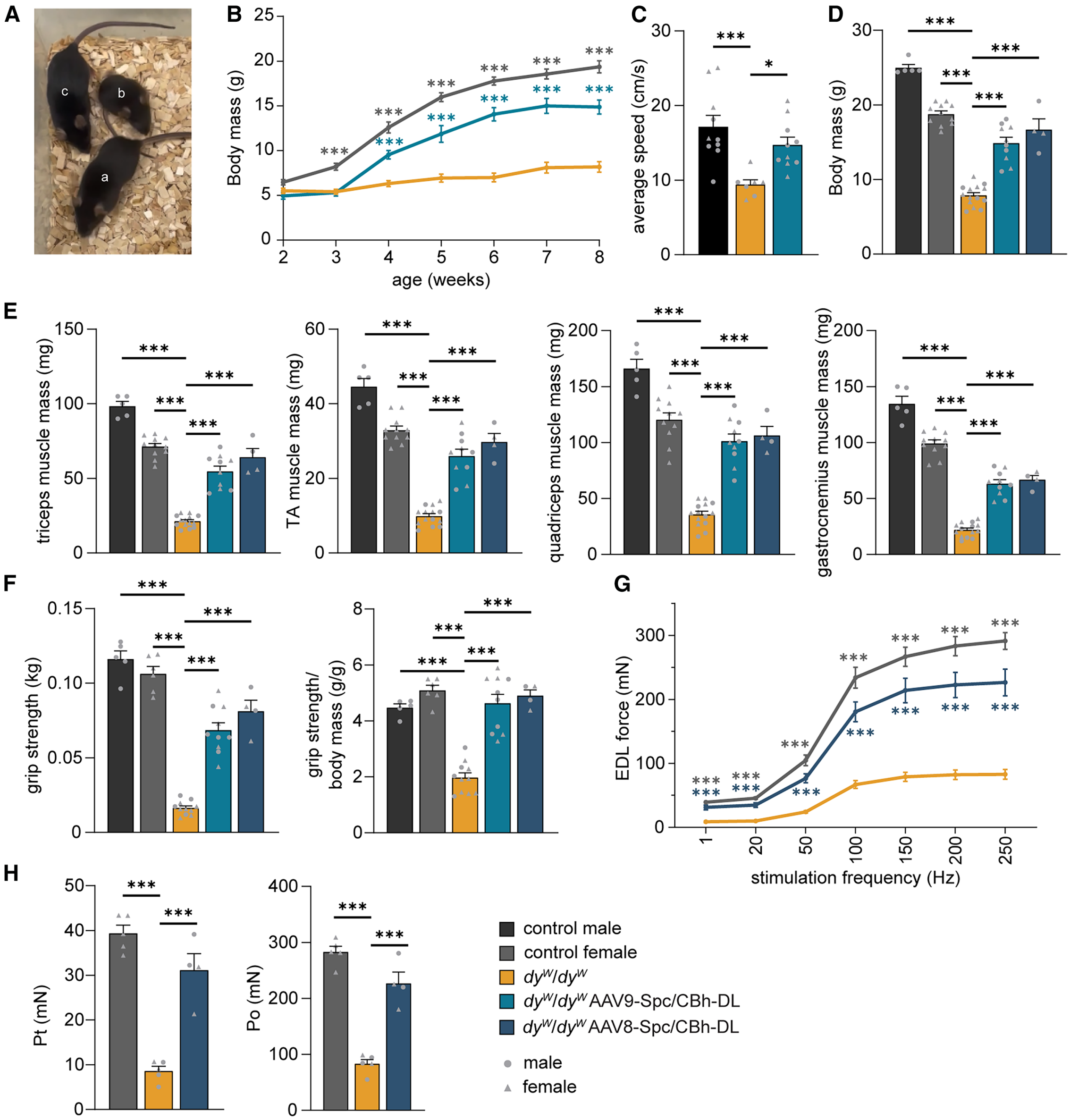

Figure 7. Functional improvement of AAV9- or AAV8-mediated dual expression of mag in muscle and αLNNdΔG2 in muscle and nerve in dy^W/dy^W mice

(A) Photograph of representative 8-week-old healthy control (a), untreated dy^W/dy^W (b), and dy^W/dy^W mice injected at P1 with AAV9-Spc/CBh-DL (1×10^{14} vg/kg per construct) (c). (B) Body mass curves from 2 to 8 weeks of age. (C) Quantitative analysis of locomotor speed at 8 weeks of age using a gait analysis system. (D–H) Analysis in 8-week-old healthy control, untreated dy^W/dy^W, and dy^W/dy^W mice injected at P1 with AAV9-Spc/CBh-DL or AAV8-Spc/CBh-DL (1×10^{14} vg/kg per construct). (D) Comparison of body mass at 8 weeks of age. (E) Comparison of the mass of fore- and hindlimb muscles from 8-week-old mice. (F) Absolute and body mass-normalized forelimb grip strength of 8-week-old mice. (G) Force frequency curves of isolated extensor digitorum longus (EDL) muscles. (H) Absolute twitch (Pt) and tetanic force (Po) measurements of isolated EDL muscles. Data are presented as mean ± SEM. *$p < 0.05$, **$p < 0.01$, and ***$p < 0.001$ by one-way ANOVA with Dunnett's post hoc test, comparing each group to untreated dy^W/dy^W mice. n = 5–14 mice per group.

and the fiber size distribution shifted toward larger fibers (Figure 8J). In summary, these results demonstrate that AAV8-Spc/CBh-DL treatment not only ameliorates the disease phenotype when administered neonatally but also provides substantial therapeutic benefit when delivered at a later stage, when both muscular dystrophy and peripheral neuropathy are advanced.

DISCUSSION

Gene replacement therapy using AAV vectors is particularly challenging for LAMA2 MD, primarily due to the large size of the *LAMA2* coding sequence, by far exceeding the packaging capacity of AAVs. Additionally, immune responses against transgene products pose a significant concern, not only threatening long-term

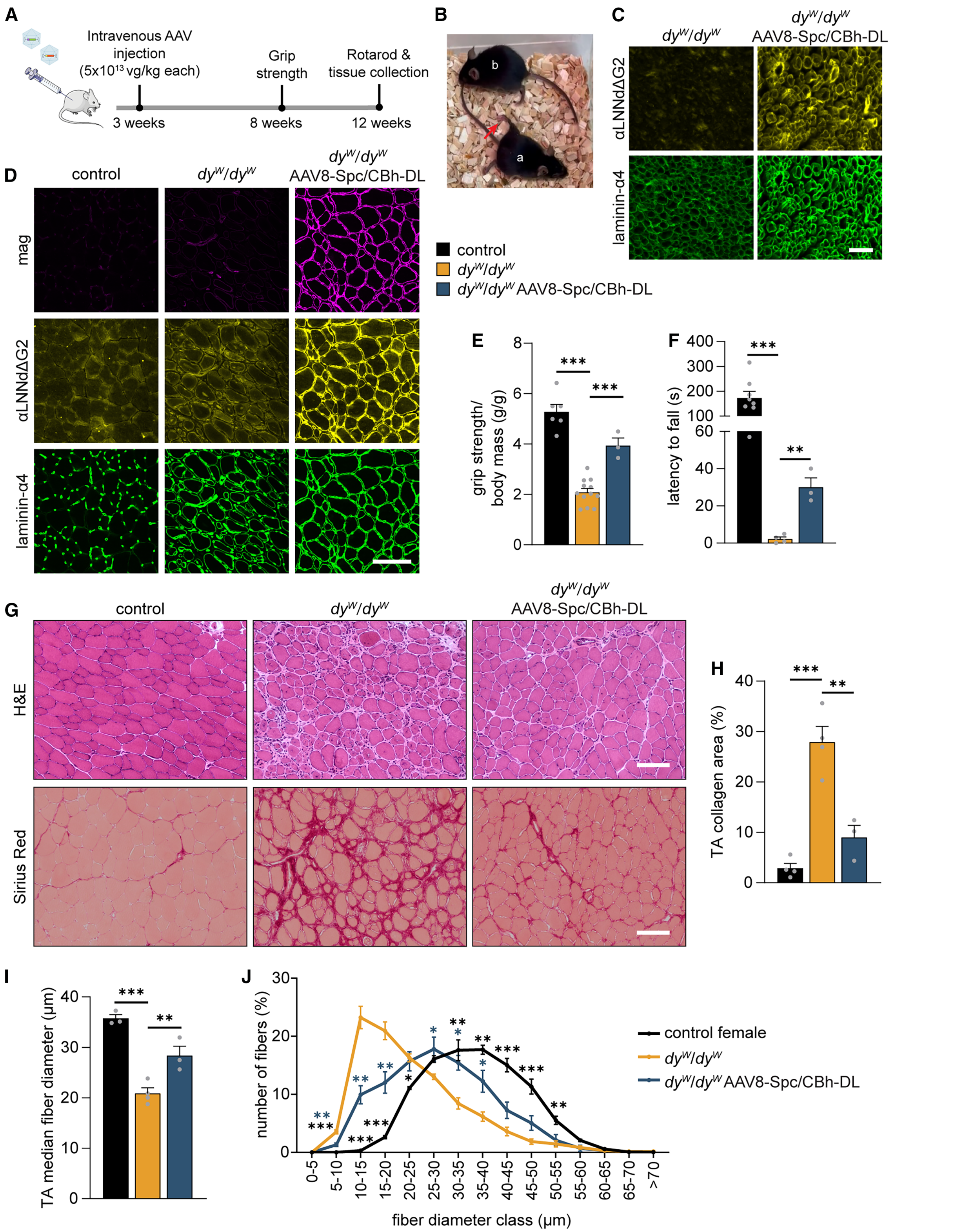

(legend on next page)

expression but also raising serious safety issues, as recently demonstrated in gene therapy trials for Duchenne muscular dystrophy.[14] Beyond the anti-transgene immune response, anti-capsid immunity represents a second major safety challenge in human applications.[51] Reports from clinical studies have described cases of thrombotic microangiopathy resulting from complement activation, particularly following administration of AAV9 or novel capsids.[52,53] To address this concern, we evaluated AAV8, for which no such adverse events have been reported to date. Our goal was to evaluate an AAV capsid with the most favorable safety profile for future human use.

In this study, we demonstrate therapeutic efficacy on both muscle and nerve pathology in the severe LAMA2 MD mouse model by using two engineered linker proteins derived from ECM components naturally found in patients. This strategy leverages the presence of laminin-4X1, an alternative laminin isoform that is consistently upregulated in LAMA2 MD but lacks sufficient receptor binding and polymerization capacity to fully compensate for the loss of laminin-2X1.[21] We have previously shown that disease progression can be mitigated by transgenic expression of mag[54–56] and further improved through the addition of a second linker protein that restores polymerization.[21] Here, we optimized these linker proteins for effective AAV-mediated delivery, with a view toward clinical translation, also employing human linker protein sequences.

Importantly, we show that muscle-restricted expression of the linker proteins improves muscular dystrophy with long-term benefits. By employing myotropic AAVs, we achieved therapeutic efficacy at lower vector doses. However, this muscle-targeted strategy was insufficient to prevent progressive neuropathy. Although peripheral neuropathy is well documented in LAMA2 MD mouse models, its clinical relevance in human patients remains less clearly defined.[12] Nerve conduction abnormalities have been reported in several patients,[57] and severe peripheral neuropathy has been described particularly in those with late-onset LAMA2 MD.[58–60] Our findings in mice highlight that while neuropathy appears mild at early stages, it progresses significantly over time, particularly when survival is extended by treating muscle pathology. This suggests that neuropathy may progress similarly in human patients unless therapeutic approaches address both muscle and nerve deficiencies.

We previously demonstrated that combined transgenic expression of both linker proteins improves nerve pathology in *Lama2*-null mice.[34] To reduce reliance on ubiquitous promoters, we now limit their use to settings where direct nerve targeting is essential. Because the mechanisms underlying nerve pathology are less well understood than those affecting muscle,[12] we generated mouse models with ubiquitous expression of each linker protein individually to dissect their respective contributions. Our data show that αLNNd alone is sufficient to ameliorate nerve pathology. In contrast, mice expressing mag in peripheral nerves still developed hindlimb paralysis, consistent with earlier findings,[61] indicating that laminin polymerization plays a particularly important role in preventing nerve pathology. Supporting this, previous studies have shown that peripheral neuropathy in laminin-α2-null mice can be rescued by transgenic expression of polymerizing laminin-α1.[62] Additionally, in models expressing truncated laminin-α2, restoration of laminin polymerization, either through a linker protein[39] or via dCas9-mediated upregulation of *Lama1*,[63] has also been shown to improve nerve pathology.

Based on these insights, we proceeded with a combination therapy consisting of muscle-specific mag and ubiquitously expressed αLNNd. To enable efficient expression, αLNNd was shortened and placed under the control of the CBh promoter. When this dual therapy was delivered neonatally via AAV8 or AAV9 at clinically relevant doses, treated dy^W/dy^W mice achieved near-normal body weight, histology, and functional outcomes.

To evaluate the global effect of treatment, we performed unbiased transcriptomic analysis, revealing normalization of gene expression across major disease-related pathways. Key dysregulated processes in LAMA2 MD muscle, including fibrosis,[64] inflammation,[45] apoptosis,[65] and reduced oxidative phosphorylation,[66] were largely corrected by the linker protein treatment. Many of these pathways are commonly implicated in other forms of muscular dystrophies, and their targeting may yield functional benefits. However, unlike interventions targeting these secondary effects, the linker protein-based therapy addresses the root cause of the disease, leading to more pronounced correction. Other mutation-independent strategies that target the primary defect and have been evaluated in mouse models include replacement of laminin-α2 with laminin-α1,[67] either through dCas9-mediated upregulation,[63] delivery of laminin-111 protein,[68] or a micro-laminin construct designed to restore some laminin-α2 features.[69] Moreover, the transcriptomic data may serve as a valuable resource for identifying biomarkers to monitor therapeutic efficacy in future clinical trials.

Figure 8. Therapeutic effects of late AAV8-mediated expression of mag in muscle and αLNNdΔG2 in muscle and nerve on muscle histology and neuromuscular function in dy^W/dy^W mice

(A) Experimental design for (B)–(J): dy^W/dy^W mice were injected intravenously at 3 weeks of age with AAV8-Spc/CBh-DL (5×10^{13} vg/kg per construct). Forelimb grip strength was analyzed at 8 weeks, rotarod performance was analyzed at 12 weeks, and tissues were collected at 12 weeks. (B) Representative photograph of a 12-week-old untreated dy^W/dy^W mouse (a) and dy^W/dy^W mouse intravenously injected at 3 weeks with AAV8-Spc/CBh-DL (b). Note the pronounced hindlimb paralysis (red arrow). (C and D) Immunofluorescence images of sciatic nerve (C) and TA (D) cross-sections stained for linker proteins and laminin-α4. (E) Forelimb grip strength normalized to body mass. (F) Rotarod-based assessment of motor function and coordination. (G) Representative images of H&E- and Sirius red-stained TA muscle cross-sections. (H) Quantification of fibrosis based on Sirius red$^+$ area in TA muscle cross-sections. (I) Quantification of median fiber diameters in TA cross-sections. (J) Distribution of fiber diameters in TA cross-sections. Data are presented as mean ± SEM. $^*p < 0.05$, $^{**}p < 0.01$, and $^{***}p < 0.001$ by one-way ANOVA with Dunnett's post hoc test or Student's *t* test (F), comparing each group to untreated dy^W/dy^W mice. Scale bars: 20 μm (C) and 100 μm (D and G). n = 3–12 mice per group.

The post-mitotic nature of muscle fibers is favorable for the long-term maintenance of AAV-delivered episomes. However, in the context of muscular dystrophy, increased muscle turnover and regeneration may dilute expression over time. In this regard, the secreted nature of the linker proteins offers a key advantage in that newly formed fibers may still benefit from linkers secreted by neighboring transduced cells. While dual-AAV approaches present challenges for cost-effective manufacturing, each individual linker confers therapeutic benefit. Due to their extracellular mode of action, both linkers need not be co-expressed in the same cell, unlike recombination, or intein-based strategies that require co-localization within single fibers.

Although laminin-2X1 surrounds muscle fibers, recent single-cell RNA-seq studies indicate that muscle fibers may not be the primary source of laminin-2X1.[70–72] This complicates gene editing strategies, which rely on targeting the correct cell type. In contrast, our linker proteins are captured by laminin-4X1 at the muscle fiber basement membrane and do not need to be expressed by the laminin-producing cells themselves.

While neonatal treatment restored near-normal muscle and nerve function, treatment at later stages was less effective, although it still conferred substantial therapeutic benefit. This reduced efficacy may be attributed to extensive fibrotic tissue accumulation, impaired muscle regeneration in advanced stages of LAMA2 MD,[73] or disruptions in integrin signaling,[74] aspects that are not yet fully explored in the context of linker protein therapy.

Consistent with previous reports, we observed no reduction in the proportion of muscle fibers with centralized nuclei in treated animals.[21,34] Interestingly, our RNA-seq data showed a marked downregulation of gene expression markers associated with active regeneration, accompanied by a decreased number of eMHC$^+$ fibers. This discrepancy suggests that centralized myonuclei in treated mice may reflect earlier degeneration-regeneration events, rather than an ongoing pathology, similar to observations following acute muscle injury.[75,76] Recent findings in other dystrophies and myopathies also reported the persistence of centralized nuclei despite clear functional improvements after treatment.[77,78] This may be explained by the initiation of therapy at a stage when substantial pathology is already present.

Although our approach is designed to promote immune tolerance, using only protein domains derived from other endogenous components of the ECM, the fusion generates novel junctions that may represent neo-epitopes that could elicit a T cell-mediated anti-transgene response. Interestingly, novel junctions are also present in all micro-dystrophins used in ongoing gene therapy approaches. However, T cell-mediated responses and immune-mediated myositis observed in a small number of treated patients were directed only against epitopes present in the endogenous protein, specifically in exons deleted in those patients, and not against the novel junctions in the micro-dystrophin construct.[14,79] Future studies will therefore be required to determine whether the junctions in the linker proteins could elicit immune responses in patients, and to assess whether their expression might cause any toxicologic effects.

In summary, this study addresses key limitations of AAV-based gene therapy for LAMA2 MD by engineering linker proteins derived from domains of other ECM components to replace the function of the missing laminin-α2 chain. These proteins can be efficiently delivered via AAV and act extracellularly, making them compatible with the constraints of AAV vector size and distribution. This strategy not only provides a practical alternative to full gene replacement but also offers an example for treating other diseases caused by mutations in large genes that exceed AAV packaging limits.

MATERIALS AND METHODS

Mice

As a mouse model for LAMA2 MD, we used dy^W/dy^W mice (B6.129S1[Cg]-Lama2tm1Eeng/J; available from The Jackson Laboratory, stock no. 013786).[35,80] Genotyping was performed as previously described.[35]

Transgenic knockin mag mice (C57BL/6J-Gt[ROSA]26Sortm1[CAG-mag]Rueg) and αLNNd mice (C57BL/6J-Gt[ROSA]26-Sortm2[CAG-aLNNd]Rueg) were described previously.[34] To achieve ubiquitous expression of the transgenes, these lines were crossed with CAG-Cre-ER mice (B6.Cg-Tg[CAG-cre/Esr1*]5Amc/J,[81] available from The Jackson Laboratory, stock no. 004682). As reported previously,[34] recombination and transgene expression occurred in muscle and peripheral nerve tissue, even in the absence of tamoxifen, and thus no tamoxifen was administered in this study.

All experimental animals were obtained from breedings of mice heterozygous for the *Lama2* knockout allele, enabling the generation of all genotypes within a single litter and the use of littermate controls. Control animals were either wild-type or heterozygous for the *Lama2* locus. For the transgenic linker protein-expressing lines, controls were always Cre$^-$ and either heterozygous or homozygous for the LSL-αLNNd and LSL-mag alleles. Both male and female mice were used in this study. As the dy^W/dy^W phenotype does not differ between sexes, dy^W/dy^W mice were randomly assigned to treatment groups after genotyping. For measurements in which healthy control values differed between males and females (e.g., body weight, muscle weight, muscle force), data were analyzed separately by sex or the sex of individual animals was indicated. Data and tissue samples from untreated healthy controls and dy^W/dy^W mice were reused across several figures. To support the dystrophic mice, all cages were equipped with long-necked water bottles and provided with wet food from weaning onward.

All animal experiments were conducted in accordance with federal guidelines and approved by the Veterinary Authority of the Canton of Basel-Stadt.

AAV vector cloning, production, purification, and titration

Mag and αLNNd constructs were subcloned into AAV plasmids containing either the Spc5.12 (Spc)[30,31] or the CBh promoter.[40] Expression cassettes were flanked by AAV2 inverted terminal repeats (ITRs) and included either a bovine growth hormone (BGH) poly(A) for mag and αLNNdΔG2, or a synthetic small poly(A)[82] for full-length αLNNd.

AAV vectors were produced by triple transfection of adherent HEK293 cells and purified by iodixanol gradient ultracentrifugation. AAVs were obtained from commercial providers (Vigene Biosciences or Charles River Laboratories) or produced in-house as previously described.[83] The following capsid and helper plasmids were used: AAV8 and AAV9 (gifts from J.M. Wilson; Addgene plasmids, catalog nos. 112864 and 112867), AAVMYO[33] (gift from D. Grimm), AAVMYO[41] (gift from D. Grimm), and pAdDeltaF6 (gift from J.M. Wilson; Addgene plasmid, catalog no. 112867). Purity and particle concentration of AAV preparations were assessed by SDS-PAGE, followed by Coomassie blue staining.

For AAV titration, unencapsidated DNA was digested with DNase I (New England Biolabs [NEB], catalog no. M0303). Next, samples were serially diluted in dilution buffer (10 mM Tris-HCl, pH 8.0, 0.1 mM EDTA, 100 μg/mL poly(A), and 0.01% Pluronic F-68). Capsids were lysed by heating to 95°C for 10 min, and viral DNA was linearized using MspI (NEB, catalog no. R0106). TaqMan-based digital PCR was performed on QIAcuity Nanoplates (26,000 24-well, Qiagen) using the QIAcuity One 5plex system (Qiagen). Primer/probe sets targeted the ITR (dPCR CGT Assay ITR2/5, FAM; catalog no. 250230), BGH poly(A) (HEX; catalog no. 250234), and either mag (forward: 5′-GGC AGA ACC TTC GTG GAG TA-3′; reverse: 5′-TCT CAG GCT CAG CTC GAA GT-3′; probe: 5′-ACG CCG TGA CCG AGA GCG AG-3′, Cy5-labeled) or αLNNd (forward: 5′-CAC CAC CAT CAT CAG ACA GG-3′; reverse: 5′-GCT GTC GGT CCA GAA GAT GT-3′; probe: 5′-CGT GGA CCA CCT GGG CAG AA-3′, Cy5-labeled).

AAV administration

Intravenous injections into neonatal mice were performed as described previously,[84] with the modification that pups were manually restrained rather than anesthetized by hypothermia. In brief, a single intravenous injection was performed on postnatal day 1 via the temporal facial vein using a 30G insulin syringe. AAV vectors were diluted in phosphate-buffered saline (PBS) and administered at a volume of 50 μL per pup. After injection, pups were placed on a heating pad and monitored until recovered before being returned to the dam.

For injections at 3 weeks of age, mice received an intravenous injection into the lateral tail vein. A total dose of 1×10^{14} vg/kg was administered at a volume of 20 μL/g of body weight.

AAV genome copy-number quantification

Tissues were pulverized in liquid nitrogen, and genomic DNA was extracted using the DNeasy Blood & Tissue Kit (Qiagen) according to the manufacturer's instructions. Viral genome copy numbers per diploid mouse genome were determined by quantitative PCR (qPCR) using a QuantStudio 5 Real-Time PCR System (Applied Biosystems) and PowerUp SYBR Green Master Mix (Applied Biosystems). Specific primers were used to amplify mag (mag_f: 5′-TGT ACA ACG GCC AGA AGA CC-3′; mag_r: 5′-TCT CTG CTT CTG ATC ACG GC-3′), αLNNd (αLNNd_f: 5′-ACA GAA TCG AGG TGG CCA AG-3′; αLNNd_r: 5′-GGT CCA GTA CAG GTT GCC TC-3′), and the mouse *Rosa26* locus (Rosa26_f: 5′-GTG GAG CCG TTC TGT GAG AC; Rosa26_r: 5′-CTT TTC CGC TCC CTT CTC CC). Copy numbers were calculated using standard curves generated from serial dilutions of the corresponding transfer plasmids and the Ai9 plasmid (a gift from H. Zeng; Addgene plasmid, catalog no. 22799) as a reference for the *Rosa26* locus.

Antibodies

The following antibodies were used for immunostaining and western blot analyses: agrin (for detection of m.mag; produced in-house[85]; αLNNd [for detection of m.αLNNd by immunostainings, previously described[86]]; 1:100); eMHC (1 μg/mL; developed by H. Blau, obtained from Developmental Studies Hybridoma Bank, catalog no. F1.652); F4/80 (Bio-Rad, catalog no. MCA497G, clone CI: A3-1; 1:50); GFP (Molecular Probes, catalog no. A10262; 1:400); laminin-α1 (for detection of αLNNd; R&D Systems, catalog no. AF4187; 1:1,000 for western blot, 1:200 for immunostaining); laminin-α2 (Sigma-Aldrich, catalog no. L0663, clone 4H8-2; 1:500); laminin-α4 (as previously described[86]; 1:1,000 for western blot, 1:200 for immunostaining) or laminin-α4 (R&D Systems, catalog no. AF3837, 1:200 for immunostaining); laminin-β1γ1 (Sigma-Aldrich, catalog no. L9393; 1:100); periostin (Abcam, catalog no. ab-14041, 1:200); and glyceraldehyde 3-phosphate dehydrogenase (Cell Signaling Technology, catalog no. 2118; 1:1,000).

Antibodies against the human version of mag were generated in-house. The mag sequence was cloned into the pCEP expression vector containing a puromycin resistance cassette and a 6× His tag. HEK293 cells were transfected using Lipofectamine 2000 (Invitrogen) according to the manufacturer's instructions and cultured in DMEM/F12 medium (Invitrogen) supplemented with 10% fetal calf serum. Two days post-transfection, medium was replaced with selection medium containing 2 μg/mL puromycin (Sigma-Aldrich), and stable clones were expanded. Upon reaching 80%–90% confluence, cells were switched to serum-free DMEM/F12. Conditioned medium was collected after 2–3 days, and protein production was validated by SDS-PAGE followed by Coomassie blue staining.

For purification, 10× supernatant buffer (500 mM NaH_2PO_4, 1.5 M NaCl, 100 mM imidazole, pH 8.0) was added to the conditioned medium, which was then incubated overnight at 4°C with rotation using pre-washed Ni-nitrilotriacetic acid agarose (Qiagen). The resin was washed four times with wash buffer (50 mM NaH_2PO_4, 300 mM NaCl, 20 mM imidazole, pH 8.0), and protein was eluted with elution buffer (50 mM NaH_2PO_4, 300 mM NaCl, 250 mM imidazole, pH

8.0). The eluate was dialyzed against PBS and purified mag-6×His was validated by SDS-PAGE and Coomassie blue staining. Protein concentration was determined using a bicinchoninic acid (BCA) assay.

Rabbit polyclonal antibodies were generated by immunizing two rabbits with purified mag-6× His (Davids Biotechnologie GmbH). Pre-immune serum from five rabbits was screened by western blot analysis and immunostaining to identify cross-reactivity. Two rabbits were selected and received five immunizations (days 1, 14, 28, 42, and 56). Test serum was collected on day 35 and the final serum on day 63. Non-purified antiserum was used for western blot analysis and immunostaining at a dilution of 1:20,000.

Immunostainings

Muscle and sciatic nerve tissues were embedded in Tissue-Tek O.C.T. compound (Sakura) and rapidly frozen in 2-methylbutane pre-cooled in liquid nitrogen (−150°C). For GFP analysis, tissues were fixed immediately after dissection in ice-cold 4% paraformaldehyde (PFA) for 2 h at 4°C, followed by dehydration in 30% sucrose overnight at 4°C, embedded in O.C.T. compound, and frozen. Frozen tissues were cryosectioned at a thickness of 10 μm and either fixed in 4% PFA in PBS or processed unfixed.

Sections were blocked for 1 h at room temperature in PBS containing 5% donkey serum and 0.3% Triton X-100, then incubated with primary antibodies diluted in PBS containing 5% donkey serum overnight at 4°C. For stainings involving mouse or rat primary antibodies, endogenous mouse immunoglobulin G was blocked using M.O.M. Blocking Reagent (Vector Laboratories, catalog no. MKB-2213; 1:40) added to the blocking buffer. Following three PBS washes, sections were incubated with appropriate secondary antibodies for 1 h at room temperature, washed again, and mounted using ProLong Gold Antifade Mountant (Invitrogen). Imaging was performed using an Olympus iX81 microscope, a Zeiss Axio Scan.Z1 slide scanner, or a Zeiss LSM700 confocal laser scanning microscope.

Histology staining and histological/immunohistological quantifications

Muscles were embedded in Tissue-Tek O.C.T. compound (Sakura) and rapidly frozen in 2-methylbutane cooled in liquid nitrogen (−150°C). Transverse sections (10 μm thickness) were cut on a cryostat. For general histological analysis, sections were fixed in 4% PFA and stained with H&E (Merck) or Picro Sirius Red (Direct Red 80; Sigma) in picric acid solution. Images were acquired using an Olympus iX81 microscope equipped with cellSens software (Olympus).

Muscle fiber diameter was quantified using the minimal Feret's diameter (the shortest distance between parallel tangents at opposing borders of the fiber), as previously described.[87] For analysis of fiber number, fiber size, and centrally nucleated fibers, complete mid-belly cross-sections were evaluated using an in-house customized, Fiji-based version of Myosoft.[34,88] The positively stained area in immunostainings (F4/80, eMHC, periostin) and Sirius red staining were quantified on the complete mid-belly section of TA muscles using Fiji. Quantitative analysis of basement membrane coverage in the sciatic nerve by αLNNdΔG2 was performed by measuring the percentage of laminin-α4$^+$ basement membrane that also contained αLNNdΔG2. Background staining from non-αLNNdΔG2-expressing wild-type mice was subtracted from the specific signal. The basement membrane area was masked using the laminin-α4 channel, and the percentage area with αLNNdΔG2 positivity was quantified in Fiji. Muscle fibrosis was quantified by measuring hydroxyproline content via MS-based amino acid analysis. Frozen muscles were pulverized in liquid nitrogen and dried using a SpeedVac. Quantification was performed as described previously.[34] For histological analysis of sciatic nerves, nerves were dissected and fixed in 2.5% glutaraldehyde in 0.12 M phosphate buffer (pH 7.4), post-fixed with 1% osmium tetroxide, embedded in Epon, and processed as previously described.[34]

Protein extraction and western blot analysis

For total protein extracts, frozen muscle or sciatic nerve tissue was pulverized in liquid nitrogen and lysed in modified radioimmunoprecipitation assay buffer (50 mM Tris-HCl, pH 8.0, 150 mM NaCl, 1% NP-40, 0.5% sodium deoxycholate, 0.1% SDS, and 20 mM EDTA, supplemented with protease inhibitors). Lysates were sonicated and incubated for 2 h at 4°C with end-over-end rotation. Insoluble material was removed by centrifugation at 16,000 × g for 30 min at 4°C. Protein concentrations were determined using the BCA assay, and samples were adjusted accordingly. Laemmli buffer was added, and samples were denatured by heating for 5 min at 95°C before subjecting them to standard SDS-PAGE and western blot analysis.

LC-MS analysis

Frozen muscle tissues were pulverized in liquid nitrogen and resuspended in lysis buffer (5% SDS, 10 mM Tris(2-carboxyethyl)phosphine, 0.1 M triethylammonium bicarbonate), followed by sonication using a PIXUL Multi-Sample Sonicator (Active Motif). Lysates were incubated at 95°C for 10 min and subsequently alkylated by 20 mM iodoacetamide at 25°C for 30 min. For each sample, 50 μg protein was digested using S-Trap micro spin columns (Protifi) according to the manufacturer's instructions, employing sequencing-grade modified trypsin (1/25, w/w; Promega). After elution, peptides were dried under vacuum. Dried peptides were resuspended in 0.1% aqueous formic acid and subjected to LC-MS/MS analysis using an Orbitrap Eclipse Tribrid Mass Spectrometer fitted with Ultimate 3000 nano system (Thermo Fisher Scientific) and a custom-made column heater set to 60°C. Peptides were separated by reverse-phase liquid chromatography (LC) using an in-house-packed C18 column (75 μm × 30 cm; ReproSil-Pur C18-AQ, 1.9 μm resin, Dr. Maisch GmbH) operated at a flow rate of 0.3 μL/min. Peptide elution was achieved using a multi-step gradient consisting of an increase from 2% to 12% buffer B over 5 min, followed by a rise to 35% B over 45 min and to 50% B over 10 min. The gradient was then ramped to 95% B within 2 min and maintained for 18 min before returning

to 2% B over 2 min and equilibrating for an additional 18 min. Buffer A contained 0.1% formic acid in water, while buffer B consisted of 80% acetonitrile with 0.1% formic acid in water.

MS data were acquired in data-independent acquisition (DIA) mode with a total cycle time of 3 s. Full MS scans (MS1) were collected in the Orbitrap at a resolution of 120,000 across a mass range of 390–920 *m/z*, using an automatic gain control (AGC) target of 1×10^6 and a maximum injection time of 45 ms. DIA acquisition comprised 46 variable windows with an isolation width of 11 *m/z* and a 1-*m/z* overlap. Fragment ion spectra were recorded over a precursor range of 400–900 *m/z* with an AGC target of 5×10^5 and a maximum injection time of 22 ms.

The acquired files were searched using the Spectronaut (Biognosys version 17.4.230317.55965) directDIA workflow against the *Mus musculus* protein database (UniProt, download February 22, 2022), with the addition of human proteins agrin, nidogen-1, and laminin-α1. Quantitative fragment ion data (F.Area) was exported from Spectronaut and analyzed using the MSstats R package version 4.8.0. (https://doi.org/10.1093/bioinformatics/btu305). Data were normalized using the default normalization option equalizedMedians, imputed using the AFT model-based imputation.

RNA-seq analysis

TA muscles were pulverized on a metal block cooled with liquid nitrogen. Total RNA was extracted using the RNeasy Fibrous Tissue Mini Kit (Qiagen) following the manufacturer's instructions. RNA concentration was quantified using the Qubit RNA HS assay (Thermo Fisher Scientific), and RNA integrity was assessed on an Agilent TapeStation. Libraries were prepared using the TruSeq Stranded mRNA Library Prep Kit version 2 (Illumina) starting from 200 ng RNA. RNA-seq was performed using the Illumina NovaSeq 6000 system (PE100), producing 150-bp paired-end reads. Sequencing resulted in 50–90 million reads per sample.

Raw transcript reads were aligned to the *Mus musculus* reference genome (GRCm38/mm10) using Salmon (version 1.1.0) with the flags validateMappings, seqBias, and gcBias. Normalization and differential expression analysis were completed using the R package DESeq2 (Bioconductor). Transcript level information was summarized to gene level. Initial filtering was applied by retaining only genes with a raw count >10 in at least three samples per group. Genes were kept only if this criterion was met in all groups.

Unsupervised sample clustering was performed using correlation distance as the distance metric and complete linkage for hierarchical clustering. Pairwise sample-to-sample correlations were visualized as a heatmap using the pheatmap R package, with annotated sample conditions.

Grip strength measurement

Forelimb grip strength was assessed in accordance to SOP MDC1A_M.2.2.001 (TREAT-NMD) using a grip strength meter (Columbus Instruments) equipped with a trapeze bar. Mice were lifted by the tail and guided toward the bar, allowing them to grasp it with both forepaws. Once a secure grip was established, the mouse was gently pulled away at a constant speed until it released the bar. The peak force was recorded, and the final force was calculated as the mean of the three highest values from six consecutive trials.

Gait analysis

Gait analysis was performed using the CatWalk XT system (Noldus) in accordance with the manufacturer's instructions. Mice were placed in an enclosed, illuminated glass walkway and allowed to move freely in both directions. Paw placement was recorded by a high-speed video camera positioned beneath the walkway. Runs were considered compliant if the mouse traversed a defined 40-cm segment within 10 s and maintained a maximum speed variation of $\leq$60%. For each mouse, three compliant runs were recorded and averaged for statistical analysis.

Assessment of motor coordination (rotarod assay)

Motor coordination was evaluated using a rotarod device (Ugo Basile). Mice were trained over 2 consecutive days with three 1-min sessions each day at a constant speed of 5 rpm. On the third day, performance was assessed on an accelerating rotarod, increasing from 5 to 40 rpm over 5 min, with a maximum trial duration of 400 s. Each mouse underwent three trials with a minimum 10-min rest between trials. The latency to fall was recorded for each trial, and the average of the three trials was used for analysis.

In vitro muscle force measurement

In vitro muscle force measurements were performed on isolated EDL using the 1200A Isolated Muscle System (Aurora Scientific). Muscles were maintained in an organ bath at 30°C containing oxygenated Ringer's solution (137 mM NaCl, 24 mM $NaHCO_3$, 11 mM glucose, 5 mM KCl, 2 mM $CaCl_2$, 1 mM $MgSO_4$, and 1 mM NaH_2PO_4) continuously bubbled with 95% O_2/5% CO_2. After adjustment to the optimal muscle length (L_0), muscles were stimulated with 15-V electrical pulses to record peak twitch force (Pt). Peak tetanic force (Po) was determined as the maximal force during a 500-ms stimulation at frequencies ranging from 10 to 250 Hz. Specific Pts and Pos were calculated by normalizing force to the muscle cross-sectional area (CSA), using the formula CSA (mm^2) = muscle wet weight (mg)/(fiber length [L_f, mm] $\times$ 1.06 mg/mm^3), where fiber length was estimated as L_f = $L_0 \times 0.44$ for EDL as previously described.[89]

Study design and statistical analysis

Mice were randomly assigned to experimental groups. Assessments of immunohistochemistry, muscle histology, muscle function, and behavioral assays were conducted by investigators blinded to group allocation. For statistical analysis, comparisons between two groups were performed using unpaired, two-tailed Student's *t* tests. For comparisons involving more than two groups, one-way ANOVA followed by either Bonferroni's or Dunnett's post hoc test was applied. Normal distribution of variables was assumed for all analyses. Statistical tests were conducted using GraphPad Prism version 9.

DATA AND CODE AVAILABILITY

RNA-seq data were deposited on NCBI Gene Expression Omnibus (GEO), accession number: GSE312724. All MS proteomics data were deposited to the ProteomicsXchange consortium via MassIVE (https://massive.ucsd.edu), accession number: MSV000098631; PRIDE: PXD066528.

ACKNOWLEDGMENTS

We thank the Imaging Core Facility, the Proteomics Core Facility, and the Animal facility of the Biozentrum. Sequencing was performed at the Genomics Facility Basel, operated by the University of Basel and the Department of Biosystems Science and Engineering (D-BSSE), ETH Zurich. We are grateful to Filippo Oliveri and Lena Jörin for technical assistance, Dr. Alexander Ham and Dr. Marco Thürkauf for their support with AAV production, Dr. Alexander Schmidt and Dr. Thomas Bock for MS analyses, and Dr. Dirk Grimm for providing the AAVMYO plasmids. Some schematics were created with BioRender.com. This work was supported by the Cantons of Basel-Stadt and Basel-Landschaft, the Swiss Foundation for Research on Muscle Disease (FSRMM), Innosuisse (grant no. 34870.1 IP-LS), Santhera Pharmaceuticals, and the Muscular Dystrophy Association (grant no. 959470; https://doi.org/10.55762/pc.gr.157038).

AUTHOR CONTRIBUTIONS

J.R.R. and M.A.R. conceived the project and designed the experiments. J.R.R. performed the majority of the experiments and analyzed the data. S.L. conducted the histological quantifications and the *in vitro* muscle force measurements. E.M. performed the RNA-seq data analysis. D.J.H. contributed to the *in vitro* muscle force measurements. J.R.R. and M.A.R. wrote the manuscript, and all authors provided feedback and approved the final version.

DECLARATION OF INTERESTS

J.R.R. and M.A.R. are co-founders and shareholders of SEAL Therapeutics AG and are inventors on a patent application filed by the University of Basel related to this work.

DECLARATION OF GENERATIVE AI AND AI-ASSISTED TECHNOLOGIES IN THE WRITING PROCESS

During the preparation of this work, the authors used ChatGPT to check grammar. After using this tool, the authors reviewed and edited the content as needed and take full responsibility for the content of the publication.

SUPPLEMENTAL INFORMATION

Supplemental information can be found online at https://doi.org/10.1016/j.ymthe.2026.01.041.

REFERENCES

1. Domogatskaya, A., Rodin, S., and Tryggvason, K. (2012). Functional diversity of laminins. Annu. Rev. Cell Dev. Biol. *28*, 523–553. https://doi.org/10.1146/annurev-cellbio-101011-155750.
2. Yurchenco, P.D. (2011). Basement membranes: cell scaffoldings and signaling platforms. Cold Spring Harb. Perspect. Biol. *3*, a004911. https://doi.org/10.1101/cshperspect.a004911.
3. Leivo, I., and Engvall, E. (1988). Merosin, a protein specific for basement membranes of Schwann cells, striated muscle, and trophoblast, is expressed late in nerve and muscle development. Proc. Natl. Acad. Sci. USA *85*, 1544–1548. https://doi.org/10.1073/pnas.85.5.1544.
4. Tome, F.M., Evangelista, T., Leclerc, A., Sunada, Y., Manole, E., Estournet, B., Barois, A., Campbell, K.P., and Fardeau, M. (1994). Congenital muscular dystrophy with merosin deficiency. C R. Acad. Sci. *317*, 351–357.
5. Geranmayeh, F., Clement, E., Feng, L.H., Sewry, C., Pagan, J., Mein, R., Abbs, S., Brueton, L., Childs, A.M., Jungbluth, H., et al. (2010). Genotype-phenotype correlation in a large population of muscular dystrophy patients with LAMA2 mutations. Neuromuscul. Disord. *20*, 241–250. https://doi.org/10.1016/j.nmd.2010.02.001.
6. Hinkley, L., Orbach, R., Park, J., Alvarez, R., Dziewczapolski, G., Bönnemann, C.G., and Foley, A.R. (2024). An international retrospective early natural history study of LAMA2-related dystrophies. J. Neuromuscul. Dis. *11*, 1238–1246. https://doi.org/10.3233/JND-240048.
7. Sarkozy, A., Foley, A.R., Zambon, A.A., Bönnemann, C.G., and Muntoni, F. (2020). LAMA2-Related Dystrophies: Clinical Phenotypes, Disease Biomarkers, and Clinical Trial Readiness. Front. Mol. Neurosci. *13*, 123. https://doi.org/10.3389/fnmol.2020.00123.
8. Zambon, A.A., Ridout, D., Main, M., Mein, R., Phadke, R., Muntoni, F., and Sarkozy, A. (2020). LAMA2-related muscular dystrophy: Natural history of a large pediatric cohort. Ann. Clin. Transl. Neurol. *7*, 1870–1882. https://doi.org/10.1002/acn3.51172.
9. Tan, D., Ge, L., Fan, Y., Chang, X., Wang, S., Wei, C., Ding, J., Liu, A., Wang, S., Li, X., et al. (2021). Natural history and genetic study of LAMA2-related muscular dystrophy in a large Chinese cohort. Orphanet J. Rare Dis. *16*, 319. https://doi.org/10.1186/s13023-021-01950-x.
10. Bonnemann, C.G., Wang, C.H., Quijano-Roy, S., Deconinck, N., Bertini, E., Ferreiro, A., Muntoni, F., Sewry, C., Beroud, C., Mathews, K.D., et al. (2014). Diagnostic approach to the congenital muscular dystrophies. Neuromuscul. Disord. *24*, 289–311. https://doi.org/10.1016/j.nmd.2013.12.011.
11. Gawlik, K.I., and Durbeej, M. (2020). A Family of Laminin alpha2 Chain-Deficient Mouse Mutants: Advancing the Research on LAMA2-CMD. Front. Mol. Neurosci. *13*, 59. https://doi.org/10.3389/fnmol.2020.00059.
12. Previtali, S.C., and Zambon, A.A. (2020). LAMA2 Neuropathies: Human Findings and Pathomechanisms From Mouse Models. Front. Mol. Neurosci. *13*, 60. https://doi.org/10.3389/fnmol.2020.00060.
13. Fields, P.A., Arruda, V.R., Armstrong, E., Chu, K., Mingozzi, F., Hagstrom, J.N., Herzog, R.W., and High, K.A. (2001). Risk and prevention of anti-factor IX formation in AAV-mediated gene transfer in the context of a large deletion of F9. Mol. Ther. *4*, 201–210. https://doi.org/10.1006/mthe.2001.0441.
14. Bonnemann, C.G., Belluscio, B.A., Braun, S., Morris, C., Singh, T., and Muntoni, F. (2023). Dystrophin Immunity after Gene Therapy for Duchenne's Muscular Dystrophy. New Engl. J. Med. *388*, 2294–2296. https://doi.org/10.1056/NEJMc2212912.
15. Cao, O., Hoffman, B.E., Moghimi, B., Nayak, S., Cooper, M., Zhou, S., Ertl, H.C.J., High, K.A., and Herzog, R.W. (2009). Impact of the underlying mutation and the route of vector administration on immune responses to factor IX in gene therapy for hemophilia B. Mol. Ther. *17*, 1733–1742. https://doi.org/10.1038/mt.2009.159.
16. Mendell, J.R., Campbell, K., Rodino-Klapac, L., Sahenk, Z., Shilling, C., Lewis, S., Bowles, D., Gray, S., Li, C., Galloway, G., et al. (2010). Dystrophin immunity in Duchenne's muscular dystrophy. N. Engl. J. Med. *363*, 1429–1437. https://doi.org/10.1056/NEJMoa1000228.
17. Pegoraro, E., Mancias, P., Swerdlow, S.H., Raikow, R.B., Garcia, C., Marks, H., Crawford, T., Carver, V., Di Cianno, B., and Hoffman, E.P. (1996). Congenital muscular dystrophy with primary laminin alpha2 (merosin) deficiency presenting as inflammatory myopathy. Ann. Neurol. *40*, 782–791. https://doi.org/10.1002/ana.410400515.
18. Hayashi, Y.K., Tezak, Z., Momoi, T., Nonaka, I., Garcia, C.A., Hoffman, E.P., and Arahata, K. (2001). Massive muscle cell degeneration in the early stage of merosin-deficient congenital muscular dystrophy. Neuromuscul. Disord. *11*, 350–359. https://doi.org/10.1016/s0960-8966(00)00203-0.
19. Durbeej, M. (2015). Laminin-alpha2 Chain-Deficient Congenital Muscular Dystrophy: Pathophysiology and Development of Treatment. Curr. Top. Membr. *76*, 31–60. https://doi.org/10.1016/bs.ctm.2015.05.002.
20. Patton, B.L., Connoll, A.M., Martin, P.T., Cunningham, J.M., Mehta, S., Pestronk, A., Miner, J.H., and Sanes, J.R. (1999). Distribution of ten laminin chains in dystrophic and regenerating muscles. Neuromuscul. Disord. *9*, 423–433. https://doi.org/10.1016/s0960-8966(99)00033-4.
21. Reinhard, J.R., Lin, S., McKee, K.K., Meinen, S., Crosson, S.C., Sury, M., Hobbs, S., Maier, G., Yurchenco, P.D., and Rüegg, M.A. (2017). Linker proteins restore basement membrane and correct LAMA2-related muscular dystrophy in mice. Sci. Transl. Med. *9*, eaal4649. https://doi.org/10.1126/scitranslmed.aal4649.
22. Denzer, A.J., Schulthess, T., Fauser, C., Schumacher, B., Kammerer, R.A., Engel, J., and Ruegg, M.A. (1998). Electron microscopic structure of agrin and mapping of its binding site in laminin-1. EMBO J. *17*, 335–343. https://doi.org/10.1093/emboj/17.2.335.
23. Gesemann, M., Cavalli, V., Denzer, A.J., Brancaccio, A., Schumacher, B., and Ruegg, M.A. (1996). Alternative splicing of agrin alters its binding to heparin, dystroglycan, and the putative agrin receptor. Neuron *16*, 755–767. https://doi.org/10.1016/s0896-6273(00)80096-3.

24. Burgess, R.W., Dickman, D.K., Nunez, L., Glass, D.J., and Sanes, J.R. (2002). Mapping sites responsible for interactions of agrin with neurons. J. Neurochem. *83*, 271–284. https://doi.org/10.1046/j.1471-4159.2002.01102.x.

25. Martin, P.T., and Sanes, J.R. (1997). Integrins mediate adhesion to agrin and modulate agrin signaling. Development *124*, 3909–3917. https://doi.org/10.1242/dev.124.19.3909.

26. Njah, K., Chakraborty, S., Qiu, B., Arumugam, S., Raju, A., Pobbati, A.V., Lakshmanan, M., Tergaonkar, V., Thibault, G., Wang, X., and Hong, W. (2019). A Role of Agrin in Maintaining the Stability of Vascular Endothelial Growth Factor Receptor-2 during Tumor Angiogenesis. Cell Rep. *28*, 949–965.e7. https://doi.org/10.1016/j.celrep.2019.06.036.

27. Ruegg, M.A., Tsim, K.W., Horton, S.E., Kröger, S., Escher, G., Gensch, E.M., and McMahan, U.J. (1992). The agrin gene codes for a family of basal lamina proteins that differ in function and distribution. Neuron *8*, 691–699. https://doi.org/10.1016/0896-6273(92)90090-z.

28. Ferns, M., Hoch, W., Campanelli, J.T., Rupp, F., Hall, Z.W., and Scheller, R.H. (1992). RNA splicing regulates agrin-mediated acetylcholine receptor clustering activity on cultured myotubes. Neuron *8*, 1079–1086. https://doi.org/10.1016/0896-6273(92)90129-2.

29. McKee, K.K., Capizzi, S., and Yurchenco, P.D. (2009). Scaffold-forming and Adhesive Contributions of Synthetic Laminin-binding Proteins to Basement Membrane Assembly. J. Biol. Chem. *284*, 8984–8994. https://doi.org/10.1074/jbc.M809719200.

30. Li, X., Eastman, E.M., Schwartz, R.J., and Draghia-Akli, R. (1999). Synthetic muscle promoters: activities exceeding naturally occurring regulatory sequences. Nat. Biotechnol. *17*, 241–245. https://doi.org/10.1038/6981.

31. Le Guiner, C., Servais, L., Montus, M., Larcher, T., Fraysse, B., Moullec, S., Allais, M., François, V., Dutilleul, M., Malerba, A., et al. (2017). Long-term microdystrophin gene therapy is effective in a canine model of Duchenne muscular dystrophy. Nat. Commun. *8*, 16105. https://doi.org/10.1038/ncomms16105.

32. Patton, B.L., Miner, J.H., Chiu, A.Y., and Sanes, J.R. (1997). Distribution and function of laminins in the neuromuscular system of developing, adult, and mutant mice. J. Cell Biol. *139*, 1507–1521.

33. Weinmann, J., Weis, S., Sippel, J., Tulalamba, W., Remes, A., El Andari, J., Herrmann, A.K., Pham, Q.H., Borowski, C., Hille, S., et al. (2020). Identification of a myotropic AAV by massively parallel *in vivo* evaluation of barcoded capsid variants. Nat. Commun. *11*, 5432. https://doi.org/10.1038/s41467-020-19230-w.

34. Reinhard, J.R., Porrello, E., Lin, S., Pelczar, P., Previtali, S.C., and Rüegg, M.A. (2023). Nerve pathology is prevented by linker proteins in mouse models for LAMA2-related muscular dystrophy. PNAS Nexus *2*, pgad083. https://doi.org/10.1093/pnasnexus/pgad083.

35. Kuang, W., Xu, H., Vachon, P.H., Liu, L., Loechel, F., Wewer, U.M., and Engvall, E. (1998). Merosin-deficient congenital muscular dystrophy. Partial genetic correction in two mouse models. J. Clin. Invest. *102*, 844–852. https://doi.org/10.1172/JCI3705.

36. Yang, D., Bierman, J., Tarumi, Y.S., Zhong, Y.P., Rangwala, R., Proctor, T.M., Miyagoe-Suzuki, Y., Takeda, S., Miner, J.H., Sherman, L.S., et al. (2005). Coordinate control of axon defasciculation and myelination by laminin-2 and -8. J. Cell Biol. *168*, 655–666. https://doi.org/10.1083/jcb.200411158.

37. Gerber, D., Pereira, J.A., Gerber, J., Tan, G., Dimitrieva, S., Yángüez, E., and Suter, U. (2021). Transcriptional profiling of mouse peripheral nerves to the single-cell level to build a sciatic nerve ATlas (SNAT). Elife *10*, e58591. https://doi.org/10.7554/eLife.58591.

38. Liberzon, A., Birger, C., Thorvaldsdóttir, H., Ghandi, M., Mesirov, J.P., and Tamayo, P. (2015). The Molecular Signatures Database (MSigDB) hallmark gene set collection. Cell Syst. *1*, 417–425. https://doi.org/10.1016/j.cels.2015.12.004.

39. McKee, K.K., and Yurchenco, P.D. (2022). Amelioration of muscle and nerve pathology of Lama2-related dystrophy by AAV9-laminin-alphaLN linker protein. JCI Insight *7*, e158397. https://doi.org/10.1172/jci.insight.158397.

40. Gray, S.J., Foti, S.B., Schwartz, J.W., Bachaboina, L., Taylor-Blake, B., Coleman, J., Ehlers, M.D., Zylka, M.J., McCown, T.J., and Samulski, R.J. (2011). Optimizing promoters for recombinant adeno-associated virus-mediated gene expression in the peripheral and central nervous system using self-complementary vectors. Hum. Gene Ther. *22*, 1143–1153. https://doi.org/10.1089/hum.2010.245.

41. El Andari, J., Renaud-Gabardos, E., Tulalamba, W., Weinmann, J., Mangin, L., Pham, Q.H., Hille, S., Bennett, A., Attebi, E., Bourges, E., et al. (2022). Semirational bioengineering of AAV vectors with increased potency and specificity for systemic gene therapy of muscle disorders. Sci. Adv. *8*, eabn4704. https://doi.org/10.1126/sciadv.abn4704.

42. Voit, A., Patel, V., Pachon, R., Shah, V., Bakhutma, M., Kohlbrenner, E., McArdle, J.J., Dell'Italia, L.J., Mendell, J.R., Xie, L.H., et al. (2017). Reducing sarcolipin expression mitigates Duchenne muscular dystrophy and associated cardiomyopathy in mice. Nat. Commun. *8*, 1068. https://doi.org/10.1038/s41467-017-01146-7.

43. Guadagnin, E., Mohassel, P., Johnson, K.R., Yang, L., Santi, M., Uapinyoying, P., Dastgir, J., Hu, Y., Dillmann, A., Cookson, M.R., et al. (2021). Transcriptome analysis of collagen VI-related muscular dystrophy muscle biopsies. Ann. Clin. Transl. Neurol. *8*, 2184–2198. https://doi.org/10.1002/acn3.51450.

44. Subramanian, A., Tamayo, P., Mootha, V.K., Mukherjee, S., Ebert, B.L., Gillette, M.A., Paulovich, A., Pomeroy, S.L., Golub, T.R., Lander, E.S., and Mesirov, J.P. (2005). Gene set enrichment analysis: a knowledge-based approach for interpreting genome-wide expression profiles. Proc. Natl. Acad. Sci. USA *102*, 15545–15550. https://doi.org/10.1073/pnas.0506580102.

45. Mehuron, T., Kumar, A., Duarte, L., Yamauchi, J., Accorsi, A., and Girgenrath, M. (2014). Dysregulation of matricellular proteins is an early signature of pathology in laminin-deficient muscular dystrophy. Skelet. Muscle *4*, 14. https://doi.org/10.1186/2044-5040-4-14.

46. Hager, M., Bigotti, M.G., Meszaros, R., Carmignac, V., Holmberg, J., Allamand, V., Akerlund, M., Kalamajski, S., Brancaccio, A., Mayer, U., and Durbeej, M. (2008). Cib2 binds integrin alpha7Bbeta1D and is reduced in laminin alpha2 chain-deficient muscular dystrophy. J. Biol. Chem. *283*, 24760–24769. https://doi.org/10.1074/jbc.M801166200.

47. Gawlik, K.I., Holmberg, J., Svensson, M., Einerborg, M., Oliveira, B.M.S., Deierborg, T., and Durbeej, M. (2017). Potent pro-inflammatory and pro-fibrotic molecules, osteopontin and galectin-3, are not major disease modulators of laminin alpha2 chain-deficient muscular dystrophy. Sci. Rep. *7*, 44059. https://doi.org/10.1038/srep44059.

48. Dastgir, J., Veerapandiyan, A., Rao, V., Tesi-Rocha, C., Harper, A., Iannacone, S., Falabella, P., Pakola, S., Philips, D., Wilson, C., et al. (2024). RGX-202, an investigational gene therapy for the treatment of Duchenne muscular dystrophy: interim clinical data. Neuromuscul. Disord. *43*, 104441.278. https://doi.org/10.1016/j.nmd.2024.07.287.

49. Laugel, V., De Lucia, S., Davion, J., Daniele, N., Cao, F., Sanz, M., Buscara, L., Blaie, S., Thibaut, L., Sagot, M., et al. (2024). GNT0004, Genethon's AAV8 vector-delivered microdystrophin gene therapy of Duchenne muscular dystrophy, first data of the phase I/II part of the GNT-016-MDYF all-in-one clinical trial in ambulant boys. Neuromuscul. Disord. *53*, 105545. https://doi.org/10.1016/j.nmd.2025.105545.

50. Zincarelli, C., Soltys, S., Rengo, G., and Rabinowitz, J.E. (2008). Analysis of AAV serotypes 1-9 mediated gene expression and tropism in mice after systemic injection. Mol. Ther. *16*, 1073–1080. https://doi.org/10.1038/mt.2008.76.

51. Keeler, A.M., Zhan, W., Ram, S., Fitzgerald, K.A., and Gao, G. (2025). The curious case of AAV immunology. Mol. Ther. *33*, 1946–1965. https://doi.org/10.1016/j.ymthe.2025.03.037.

52. Byrne, B.J., Butterfield, R.J., Shieh, P.B., Smith, E.C., Licht, C., Binks, M., Casinghino, S., Delnomdedieu, M., Ravindra, K.C., McDonnell, T., et al. (2025). Complement activation in a phase Ib study of fordadistrogene movaparvovec for Duchenne muscular dystrophy. Mol. Ther. *33*, 4226–4238. https://doi.org/10.1016/j.ymthe.2025.06.032.

53. Chand, D.H., Zaidman, C., Arya, K., Millner, R., Farrar, M.A., Mackie, F.E., Goedeker, N.L., Dharnidharka, V.R., Dandamudi, R., and Reyna, S.P. (2021). Thrombotic Microangiopathy Following Onasemnogene Abeparvovec for Spinal Muscular Atrophy: A Case Series. J. Pediatr. *231*, 265–268. https://doi.org/10.1016/j.jpeds.2020.11.054.

54. Moll, J., Barzaghi, P., Lin, S., Bezakova, G., Lochmüller, H., Engvall, E., Müller, U., and Ruegg, M.A. (2001). An agrin minigene rescues dystrophic symptoms in a mouse model for congenital muscular dystrophy. Nature *413*, 302–307. https://doi.org/10.1038/35095054.

55. Meinen, S., Barzaghi, P., Lin, S., Lochmüller, H., and Ruegg, M.A. (2007). Linker molecules between laminins and dystroglycan ameliorate laminin-alpha2-deficient muscular dystrophy at all disease stages. J. Cell Biol. *176*, 979–993. https://doi.org/10.1083/jcb.200611152.

56. Bentzinger, C.F., Barzaghi, P., Lin, S., and Ruegg, M.A. (2005). Overexpression of mini-agrin in skeletal muscle increases muscle integrity and regenerative capacity in laminin-alpha2-deficient mice. FASEB J. *19*, 934–942. https://doi.org/10.1096/fj.04-3376com.

57. Quijano-Roy, S., Renault, F., Romero, N., Guicheney, P., Fardeau, M., and Estournet, B. (2004). EMG and nerve conduction studies in children with congenital muscular dystrophy. Muscle Nerve *29*, 292–299. https://doi.org/10.1002/mus.10544.

58. Di Muzio, A., De Angelis, M.V., Di Fulvio, P., Ratti, A., Pizzuti, A., Stuppia, L., Gambi, D., and Uncini, A. (2003). Dysmyelinating sensory-motor neuropathy in merosin-deficient congenital muscular dystrophy. Muscle Nerve *27*, 500–506. https://doi.org/10.1002/mus.10326.

59. Chan, S.H.S., Foley, A.R., Phadke, R., Mathew, A.A., Pitt, M., Sewry, C., and Muntoni, F. (2014). Limb girdle muscular dystrophy due to LAMA2 mutations: diagnostic difficulties due to associated peripheral neuropathy. Neuromuscul. Disord. *24*, 677–683. https://doi.org/10.1016/j.nmd.2014.05.008.

60. Shorer, Z., Philpot, J., Muntoni, F., Sewry, C., and Dubowitz, V. (1995). Demyelinating peripheral neuropathy in merosin-deficient congenital muscular dystrophy. J. Child Neurol. *10*, 472–475. https://doi.org/10.1177/088307389501000610.

61. Qiao, C., Dai, Y., Nikolova, V.D., Jin, Q., Li, J., Xiao, B., Li, J., Moy, S.S., and Xiao, X. (2018). Amelioration of Muscle and Nerve Pathology in LAMA2 Muscular Dystrophy by AAV9-Mini-Agrin. Mol. Ther. Methods Clin. Dev. *9*, 47–56. https://doi.org/10.1016/j.omtm.2018.01.005.

62. Gawlik, K.I., Li, J.Y., Petersén, A., and Durbeej, M. (2006). Laminin alpha1 chain improves laminin alpha2 chain deficient peripheral neuropathy. Hum. Mol. Genet. *15*, 2690–2700. https://doi.org/10.1093/hmg/ddl201.

63. Kemaladewi, D.U., Bassi, P.S., Erwood, S., Al-Basha, D., Gawlik, K.I., Lindsay, K., Hyatt, E., Kember, R., Place, K.M., Marks, R.M., et al. (2019). A mutation-independent approach for muscular dystrophy via upregulation of a modifier gene. Nature *572*, 125–130. https://doi.org/10.1038/s41586-019-1430-x.

64. Accorsi, A., Cramer, M.L., and Girgenrath, M. (2020). Fibrogenesis in LAMA2-Related Muscular Dystrophy Is a Central Tenet of Disease Etiology. Front. Mol. Neurosci. *13*, 3. https://doi.org/10.3389/fnmol.2020.00003.

65. Girgenrath, M., Dominov, J.A., Kostek, C.A., and Miller, J.B. (2004). Inhibition of apoptosis improves outcome in a model of congenital muscular dystrophy. J. Clin. Invest. *114*, 1635–1639. https://doi.org/10.1172/JCI22928.

66. de Oliveira, B.M., Matsumura, C.Y., Fontes-Oliveira, C.C., Gawlik, K.I., Acosta, H., Wernhoff, P., and Durbeej, M. (2014). Quantitative proteomic analysis reveals metabolic alterations, calcium dysregulation, and increased expression of extracellular matrix proteins in laminin alpha2 chain-deficient muscle. Mol. Cell. Proteomics *13*, 3001–3013. https://doi.org/10.1074/mcp.M113.032276.

67. Gawlik, K., Miyagoe-Suzuki, Y., Ekblom, P., Takeda, S., and Durbeej, M. (2004). Laminin alpha1 chain reduces muscular dystrophy in laminin alpha2 chain deficient mice. Hum. Mol. Genet. *13*, 1775–1784. https://doi.org/10.1093/hmg/ddh190.

68. Rooney, J.E., Knapp, J.R., Hodges, B.L., Wuebbles, R.D., and Burkin, D.J. (2012). Laminin-111 protein therapy reduces muscle pathology and improves viability of a mouse model of merosin-deficient congenital muscular dystrophy. Am. J. Pathol. *180*, 1593–1602. https://doi.org/10.1016/j.ajpath.2011.12.019.

69. Packer, D., and Martin, P.T. (2021). Micro-laminin gene therapy can function as an inhibitor of muscle disease in the dy(W) mouse model of MDC1A. Mol. Ther. Methods Clin. Dev. *21*, 274–287. https://doi.org/10.1016/j.omtm.2021.02.004.

70. Petrany, M.J., Swoboda, C.O., Sun, C., Chetal, K., Chen, X., Weirauch, M.T., Salomonis, N., and Millay, D.P. (2020). Single-nucleus RNA-seq identifies transcriptional heterogeneity in multinucleated skeletal myofibers. Nat. Commun. *11*, 6374. https://doi.org/10.1038/s41467-020-20063-w.

71. Ham, A.S., Lin, S., Tse, A., Thürkauf, M., McGowan, T.J., Jörin, L., Oliveri, F., and Rüegg, M.A. (2025). Single-nuclei sequencing of skeletal muscle reveals subsynaptic-specific transcripts involved in neuromuscular junction maintenance. Nat. Commun. *16*, 2220. https://doi.org/10.1038/s41467-025-57487-1.

72. Eraslan, G., Drokhlyansky, E., Anand, S., Fiskin, E., Subramanian, A., Slyper, M., Wang, J., Van Wittenberghe, N., Rouhana, J.M., Waldman, J., et al. (2022). Single-nucleus cross-tissue molecular reference maps toward understanding disease gene function. Science *376*, eabl4290. https://doi.org/10.1126/science.abl4290.

73. McGowan, T.J., Reinhard, J.R., Lewerenz, N., Białobrzeska, M., Lin, S., Stępniewski, J., Szade, K., Dulak, J., and Rüegg, M.A. (2025). Loss of cell-autonomously secreted laminin-alpha2 drives muscle stem cell dysfunction in LAMA2-related muscular dystrophy. Nat. Commun. *16*, 10674. https://doi.org/10.1038/s41467-025-65703-1.

74. Barraza-Flores, P., Bates, C.R., Oliveira-Santos, A., and Burkin, D.J. (2020). Laminin and Integrin in LAMA2-Related Congenital Muscular Dystrophy: From Disease to Therapeutics. Front. Mol. Neurosci. *13*, 1. https://doi.org/10.3389/fnmol.2020.00001.

75. Meyer, G.A. (2018). Evidence of induced muscle regeneration persists for years in the mouse. Muscle Nerve *58*, 858–862. https://doi.org/10.1002/mus.26329.

76. Collins, B.C., Shapiro, J.B., Scheib, M.M., Musci, R.V., Verma, M., and Kardon, G. (2024). Three-dimensional imaging studies in mice identify cellular dynamics of skeletal muscle regeneration. Dev. Cell *59*, 1457–1474.e5. https://doi.org/10.1016/j.devcel.2024.03.017.

77. Gushchina, L.V., Bradley, A.J., Vetter, T.A., Lay, J.W., Rohan, N.L., Frair, E.C., Wein, N., and Flanigan, K.M. (2023). Persistence of exon 2 skipping and dystrophin expression at 18 months after U7snRNA-mediated therapy in the Dup2 mouse model. Mol. Ther. Methods Clin. Dev. *31*, 101144. https://doi.org/10.1016/j.omtm.2023.101144.

78. Shieh, P.B., Kuntz, N.L., Dowling, J.J., Müller-Felber, W., Bönnemann, C.G., Seferian, A.M., Servais, L., Smith, B.K., Muntoni, F., Blaschek, A., et al. (2023). Safety and efficacy of gene replacement therapy for X-linked myotubular myopathy (ASPIRO): a multinational, open-label, dose-escalation trial. Lancet Neurol. *22*, 1125–1139. https://doi.org/10.1016/S1474-4422(23)00313-7.

79. Khan, S., Haegel, H., Hollenstein, A., Wandel, C., Wagner, K., Asher, D., Griffin, D., Potter, R., Moeller, I., Singh, T., and Rodino-Klapac, L. (2023). T-cell response to SRP-9001 dystrophin transgene in a patient treated with Delandistrogene Moxeparvovec: a case of immune-mediated myositis. Neuromuscul. Disord. *33*, S72.

80. Kuang, W., Xu, H., Vilquin, J.T., and Engvall, E. (1999). Activation of the lama2 gene in muscle regeneration: abortive regeneration in laminin alpha2-deficiency. Lab. Invest. *79*, 1601–1613.

81. Hayashi, S., and McMahon, A.P. (2002). Efficient recombination in diverse tissues by a tamoxifen-inducible form of Cre: a tool for temporally regulated gene activation/inactivation in the mouse. Dev. Biol. *244*, 305–318. https://doi.org/10.1006/dbio.2002.0597.

82. Senis, E., Fatouros, C., Grosse, S., Wiedtke, E., Niopek, D., Mueller, A.K., Borner, K., and Grimm, D. (2014). CRISPR/Cas9-mediated genome engineering: an adeno-associated viral (AAV) vector toolbox. Biotechnol. J. *9*, 1402–1412. https://doi.org/10.1002/biot.201400046.

83. Thurkauf, M., Lin, S., Oliveri, F., Grimm, D., Platt, R.J., and Ruegg, M.A. (2023). Fast, multiplexable and efficient somatic gene deletions in adult mouse skeletal muscle fibers using AAV-CRISPR/Cas9. Nat. Commun. *14*, 6116. https://doi.org/10.1038/s41467-023-41769-7.

84. Gombash Lampe, S.E., Kaspar, B.K., and Foust, K.D. (2014). Intravenous injections in neonatal mice. J. Vis. Exp. *93*, e52037. https://doi.org/10.3791/52037.

85. Eusebio, A., Oliveri, F., Barzaghi, P., and Ruegg, M.A. (2003). Expression of mouse agrin in normal, denervated and dystrophic muscle. Neuromuscul. Disord. *13*, 408–415. https://doi.org/10.1016/s0960-8966(03)00036-1.

86. McKee, K.K., Crosson, S.C., Meinen, S., Reinhard, J.R., Rüegg, M.A., and Yurchenco, P.D. (2017). Chimeric protein repair of laminin polymerization ameliorates muscular dystrophy phenotype. J. Clin. Invest. *127*, 1075–1089. https://doi.org/10.1172/JCI90854.

87. Briguet, A., Courdier-Fruh, I., Foster, M., Meier, T., and Magyar, J.P. (2004). Histological parameters for the quantitative assessment of muscular dystrophy in the mdx-mouse. Neuromuscul. Disord. *14*, 675–682. https://doi.org/10.1016/j.nmd.2004.06.008.

88. Encarnacion-Rivera, L., Foltz, S., Hartzell, H.C., and Choo, H. (2020). Myosoft: An automated muscle histology analysis tool using machine learning algorithm utilizing FIJI/ImageJ software. PLoS One *15*, e0229041. https://doi.org/10.1371/journal.pone.0229041.

89. Brooks, S.V., and Faulkner, J.A. (1988). Contractile properties of skeletal muscles from young, adult and aged mice. J. Physiol. *404*, 71–82. https://doi.org/10.1113/jphysiol.1988.sp017279.

YMTHE, Volume *34*

Supplemental Information

Dual AAV gene therapy using laminin-linking proteins ameliorates muscle and nerve defects in LAMA2-related muscular dystrophy

Judith R. Reinhard, Shuo Lin, Eleonora Maino, Daniel J. Ham, and Markus A. Rüegg

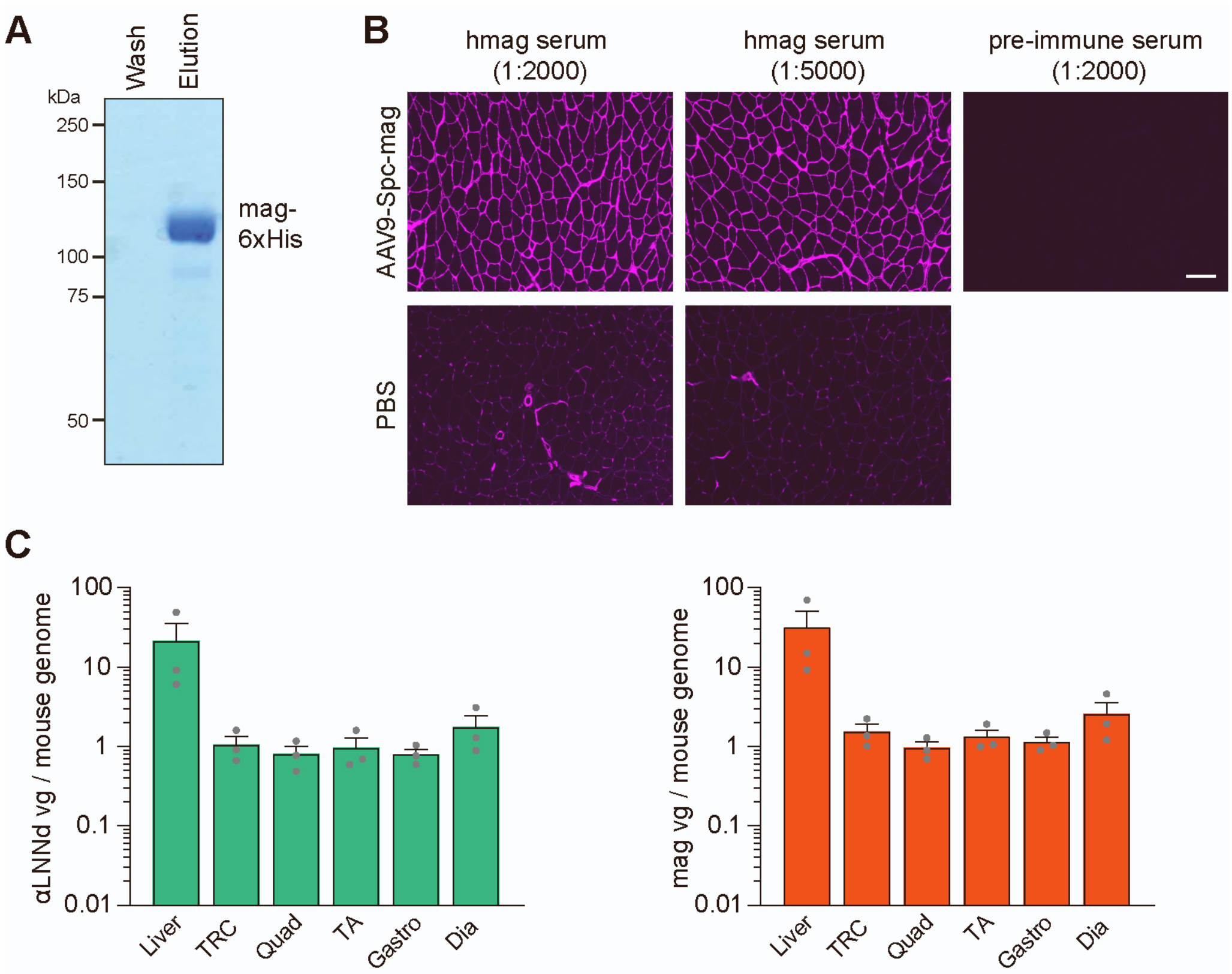

Figure S1. Generation of anti-human mag antisera and assessment of AAV9-Spc-DL systemic transduction efficacy. (**A**) Coomassie Blue-stained SDS-PAGE of wash and eluate fractions from Ni-NTA columns loaded with supernatant from transiently transfected HEK293 cells expressing mag-6×His. (**B**) Immunofluorescence images of triceps muscle cross-sections from 8-week-old C57BL/6 mice injected intravenously at P1 with AAV9-Spc-mag (1.5×10^{14} vg/kg) or vehicle (PBS). Sections were stained using serum (various dilutions) from a rabbit immunized with mag or with pre-immune serum from the same rabbit. (**C**) Quantification of vector genomes (vg) normalized to mouse genomic DNA by qPCR from different tissues of 8-week-old C57BL/6 mice injected at P1 with AAV9-Spc-αLNNd and AAV9-Spc-mag (1.5×10^{14} vg/kg per construct). Abbreviations are: Triceps brachii (TRC), quadriceps (Quad), tibialis anterior (TA), gastrocnemius (Gastro) and diaphragm (Dia). Data are presented as mean ± SEM. Scale bar: 100 µm. *n* = 3 mice per group.

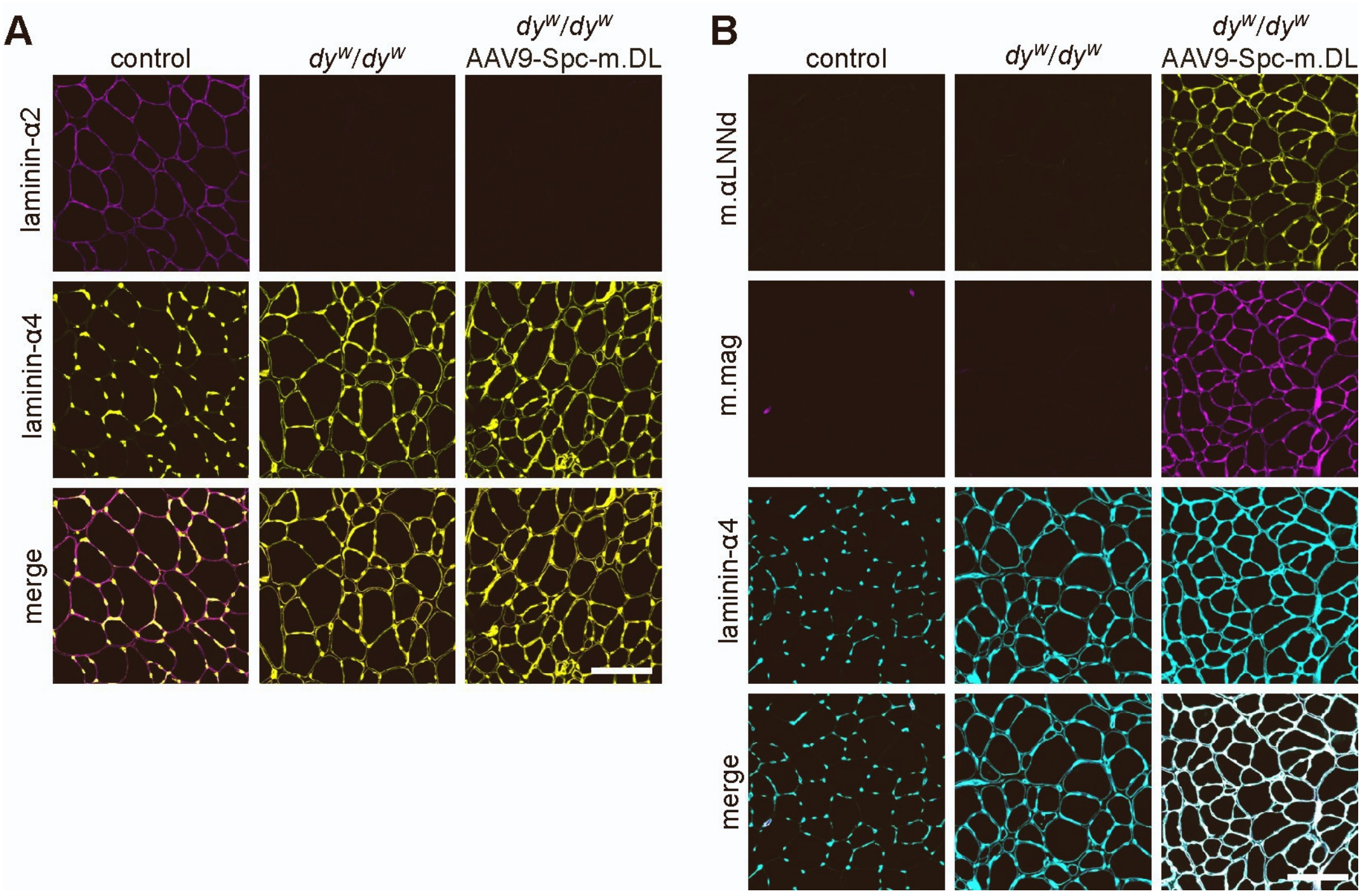

Figure S2. Laminin and linker protein localization in *dy^W/dy^W* mice and *dy^W/dy^W* AAV9-Spc-m.DL mice.
(**A-B**) Representative immunofluorescence images of cross-sections form TA muscle of 8-week-old non-injected healthy controls, untreated dy^W/dy^W mice, and dy^W/dy^W mice injected intravenously at P1 with AAV9-Spc-m.DL (1.5×10^{14} vg/kg per construct). Cross-sections were stained for laminin-α2 and laminin-α4 (**A**), or for m.αLNNd, m.mag and laminin-α4 (**B**). Note that laminin-α4 localizes to blood vessels in healthy controls but appears in the muscle fiber ECM in dy^W/dy^W mice and in dy^W/dy^W mice injected with AAV9-Spc-m.DL. Scale bars: 100 µm

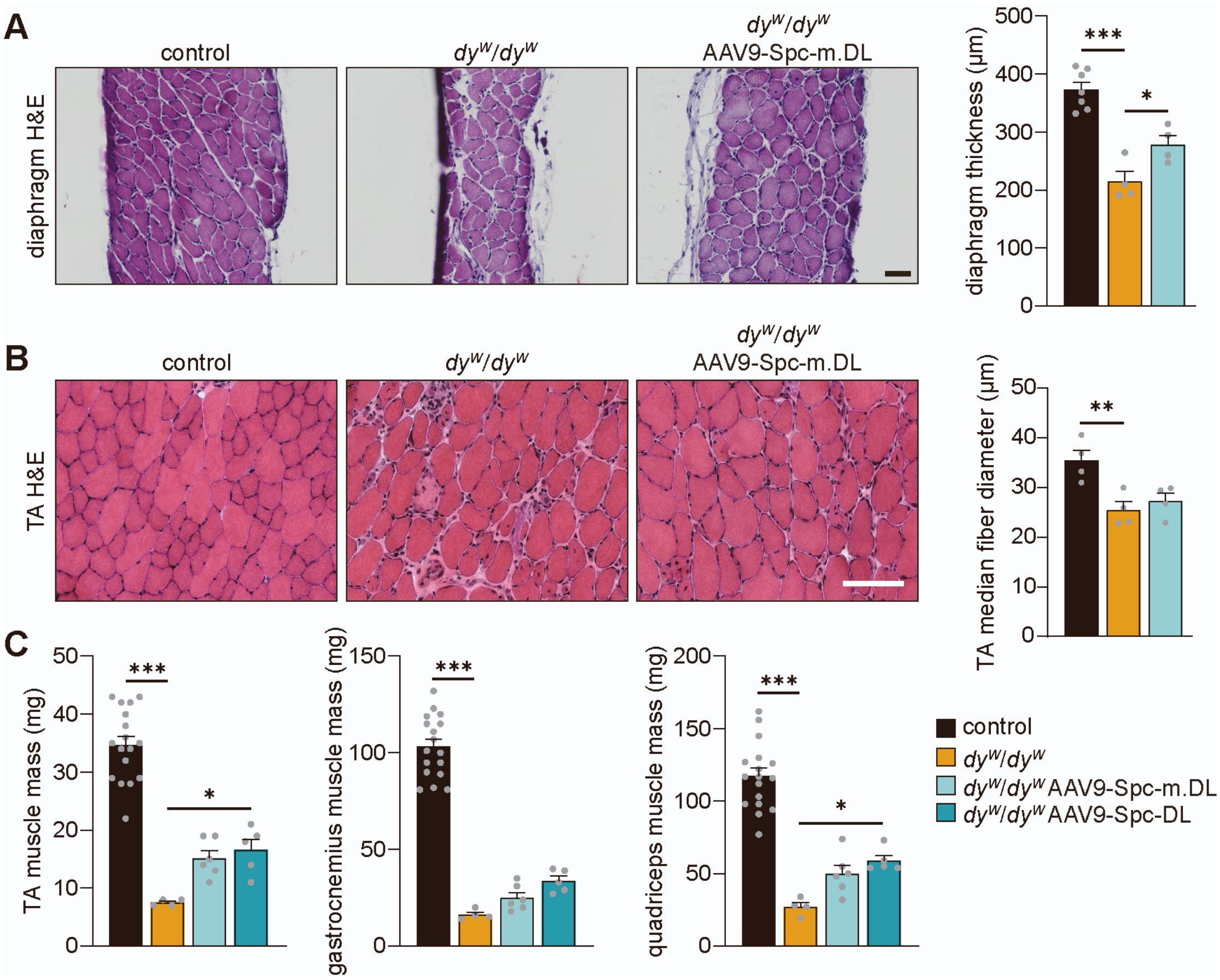

Figure S3. Therapeutic benefit of AAV-mediated, muscle-targeted expression of linker proteins in *dy^W/dy^W* mice. (**A-C**) Histology and muscle mass of non-injected healthy control, *dy^W/dy^W* mice, and *dy^W/dy^W* mice injected intravenously at P1 with AAV9-Spc-m.DL or the corresponding human linker constructs (AAV9-Spc-DL) (1.5×10^{14} vg/kg per construct). (**A-B**) Representative H&E-stained cross-sections of diaphragm (**A**) or TA (**B**) from 8-week-old mice, with quantification of diaphragm thickness (**A**) and TA median fiber diameter (**B**). (**C**) Muscle mass in 8-week-old mice. Data are presented as mean ± SEM. **P* < 0.05, ***P* < 0.01, ****P* < 0.001 by one-way ANOVA with Dunnett's post hoc test, comparing each group to untreated *dy^W/dy^W* mice. Scale bars: 50 μm (A), 100 μm (B). *n* = 4-17 mice per group.

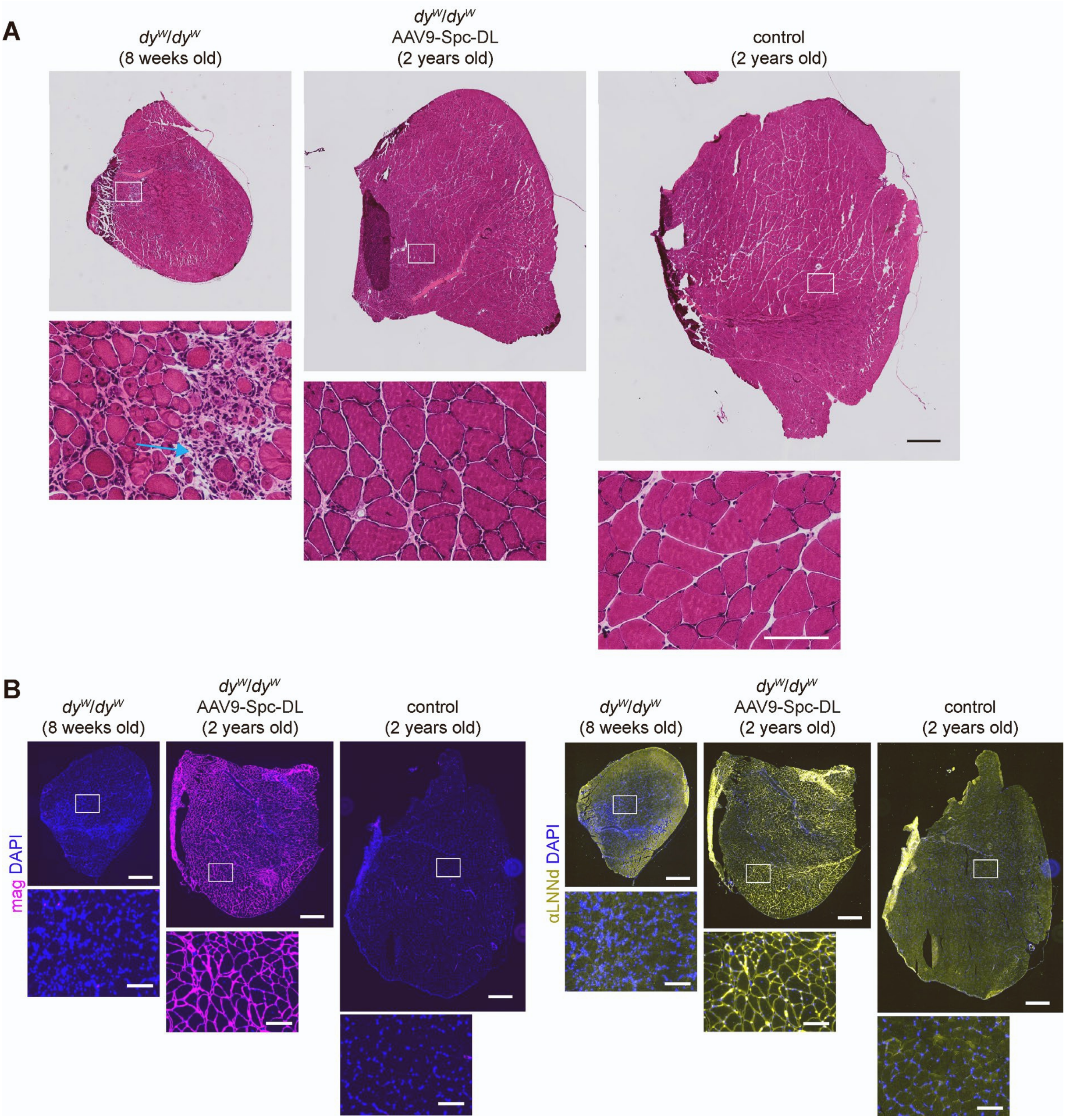

Figure S4. Long-term expression and sustained therapeutic benefit of AAV-mediated, muscle-targeted expression of linker proteins in *dy*^{W}/*dy*^{W} mice.

(**A-B**) Histology and immunofluorescence of non-injected healthy control, *dy*^{W}/*dy*^{W} mice, and *dy*^{W}/*dy*^{W} mice injected intravenously at P1 with AAV9-Spc-DL (1.5×10^{14} vg/kg per construct). (**A**) Representative H&E staining of triceps muscle cross-sections from an 8-week-old *dy*^{W}/*dy*^{W} mouse, a 2-year-old *dy*^{W}/*dy*^{W} mouse injected with AAV9-Spc-DL and a 2-year-old healthy control. Notably, muscle from untreated mice *dy*^{W}/*dy*^{W} showed mononuclear cell infiltrates (arrow), which were absent in the treated *dy*^{W}/*dy*^{W} mouse. (**B**) Immunofluorescence images stained for mag and αLNNd. Scale bars: 500 µm (A and B, top) and 100 µm (A and B, bottom).

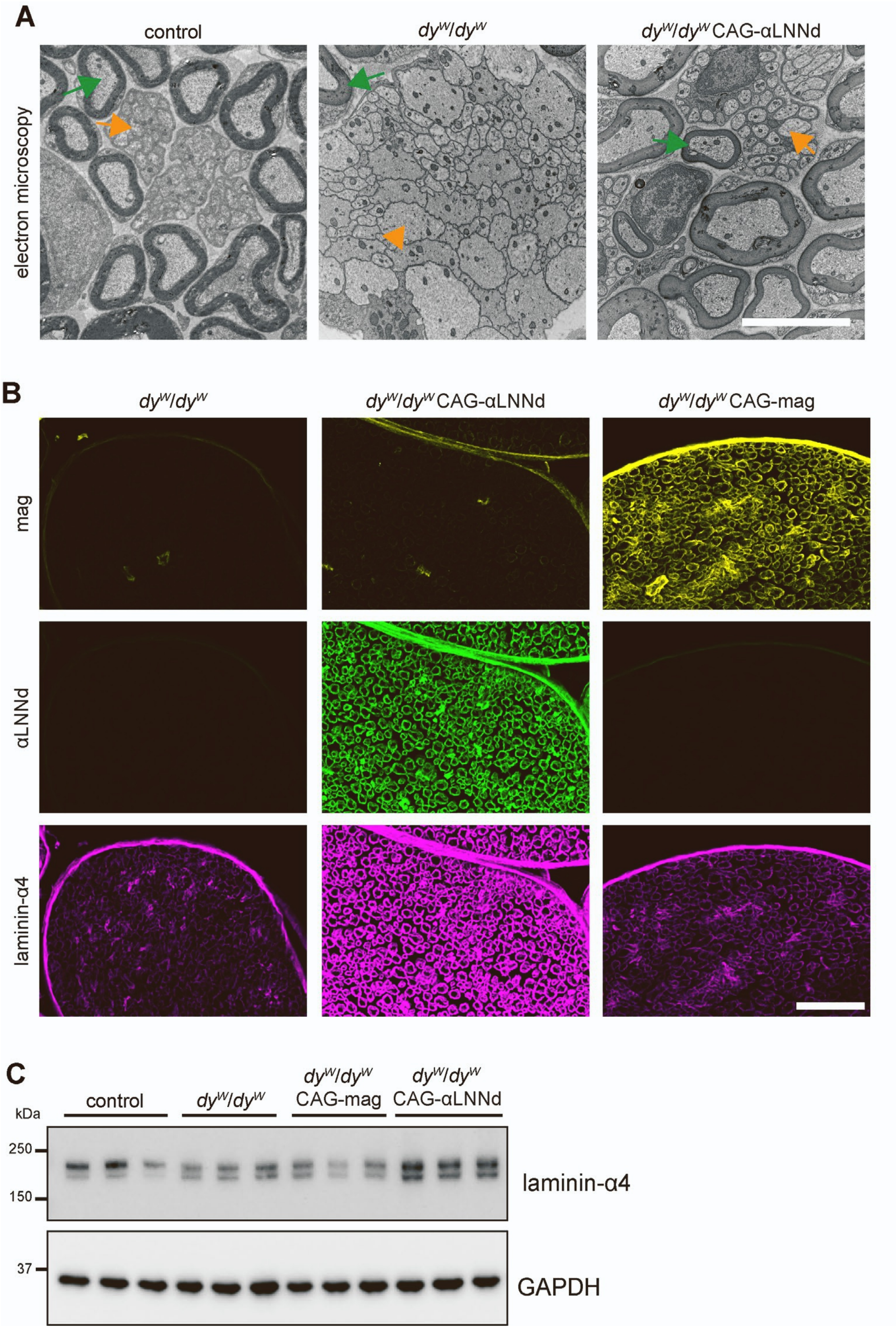

Figure S5. Restoration of axonal sorting and increase in laminin-α4 in sciatic nerve following ubiquitous transgenic expression of αLNNd.
(**A-C**) Analysis of sciatic nerve pathology and laminin-α4 levels in 8-week-old healthy control mice, *dy*^{W}/*dy*^{W} mice, and *dy*^{W}/*dy*^{W} transgenic mice that ubiquitously express mag or αLNNd under the control of the CAG promoter (CAG-mag or CAG-αLNNd). (**A**) Representative images of transmission electron microscopy of sciatic nerve from mice with the indicated genotypes. Note that in healthy controls, large-caliber motor axons are typically myelinated (green arrows), whereas small-diameter sensory axons are unmyelinated, ensheathed by non-myelinating Schwann cells, and clustered in Remak bundles (orange arrows). In contrast, *dy*^{W}/*dy*^{W} mice display large regions of unsorted, non-myelinated axons, including large-caliber axons (orange arrowhead) not ensheathed by myelinating Schwann cells. In *dy*^{W}/*dy*^{W} mice expressing CAG-αLNNd, axonal sorting appears similar to that observed in healthy controls with myelinated large axons (green arrow) and Remak bundles (orange arrow). (**B**) Immunofluorescence images of sciatic nerve cross-sections stained for mag, αLNNd and laminin-α4. Please note the strong increase in laminin-α4 in *dy*^{W}/*dy*^{W} CAG-αLNNd mice compared to the other two genotypes. (**C**) Western blot analysis of laminin-α4 in sciatic nerve lysates. GAPDH was used as loading control. Scale bars: 5 µm (A), 50 µm (B).

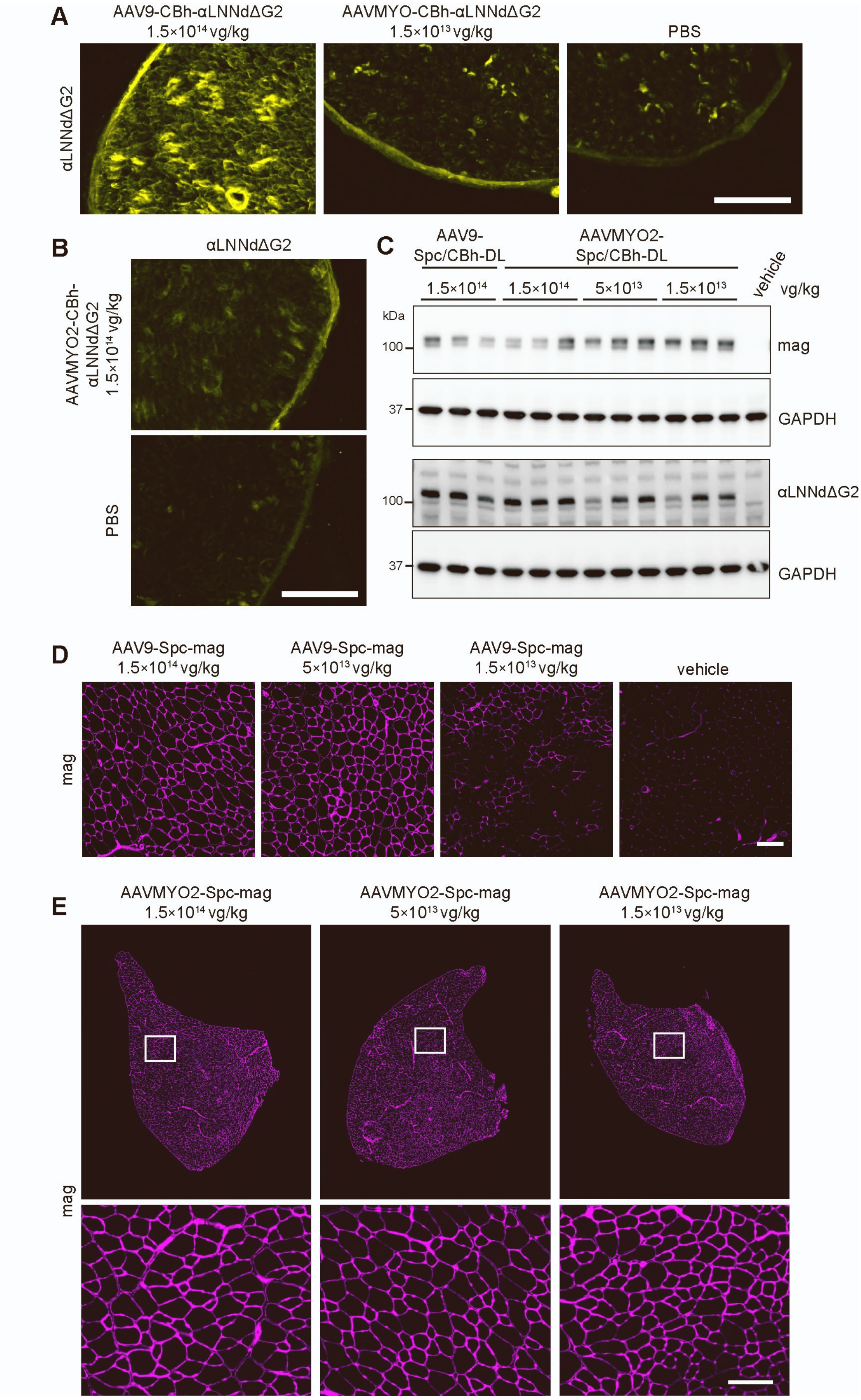

Figure S6. Comparison of AAVMYO, AAVMYO2, and AAV9-mediated expression in sciatic nerve and skeletal muscle. (**A-D**) Analysis of linker protein amount in 8-week-old C57BL/6 mice injected intravenously at P1 with the indicated construct and doses. (**A-B**) Immunofluorescence images of sciatic nerve cross-sections stained for αLNNdΔG2. Notably, only AAV9-mediated transduction resulted in αLNNdΔG2-positive basement membranes within the endoneurium. Some background staining was also observed in axons of PBS-injected mice. (**C**) Western blot analysis of triceps muscle lysate to detect αLNNdΔG2 or mag. GAPDH was used as loading control. (**D-E**) Immunofluorescence staining for mag of TA muscle cross-sections. Scale bars: 50 µm (A, B), 100 µm (D, E).

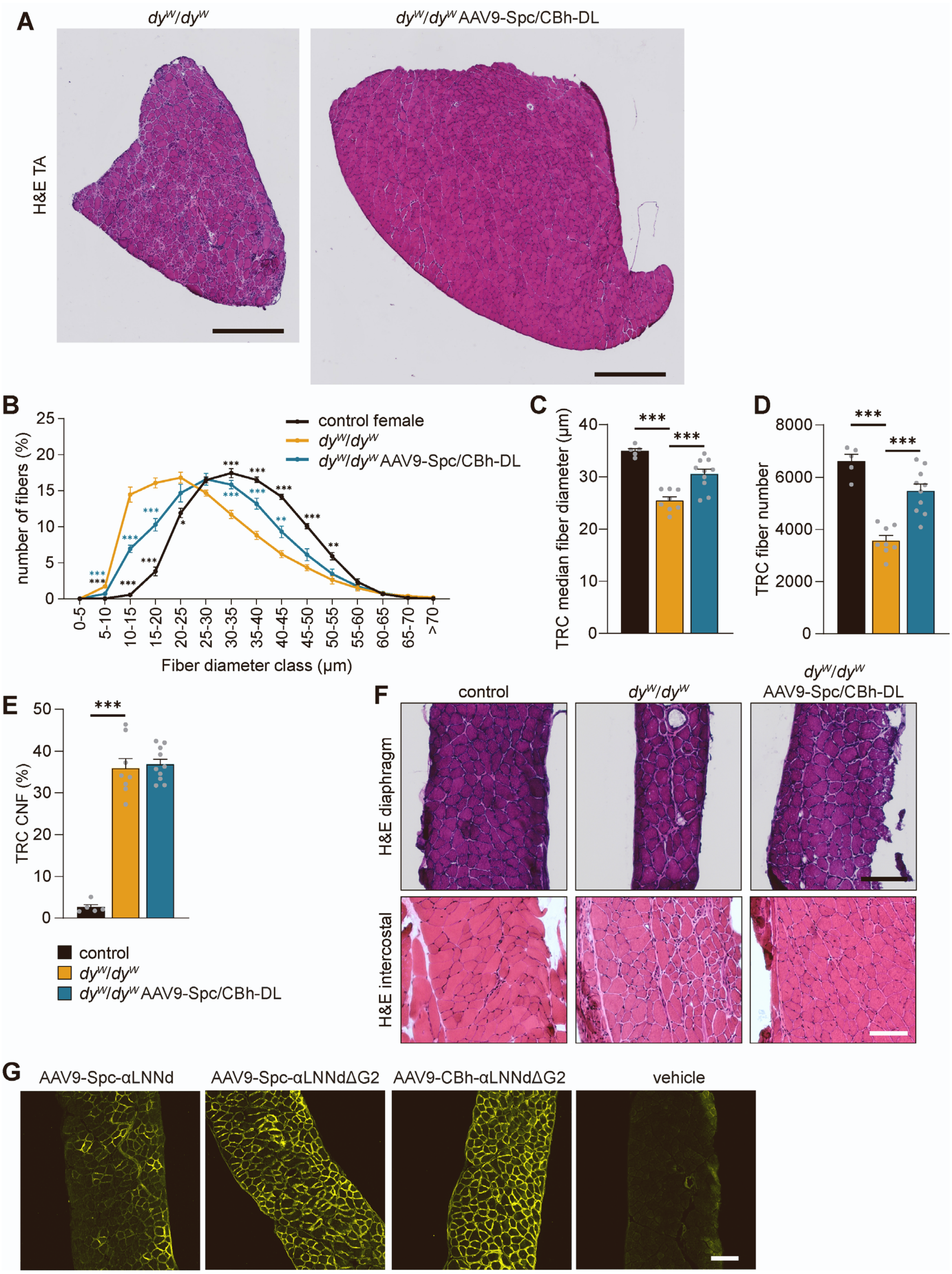

Figure S7. AAV9-mediated improvement of histology in TA, triceps and respiratory muscles in *dy^{W}/dy^{W}* AAV9-Spc/CBh-DL mice.

(**A-F**) Histological analysis of skeletal muscle from 8-week-old healthy control, *dy^{W}/dy^{W}* and *dy^{W}/dy^{W}* mice injected intravenously at P1 with AAV9-Spc/CBh-DL (1×10^{14} vg/kg per construct). (**A**) Representative H&E-stained cross-sections of TA muscle. (**B-E**) Quantification of triceps muscle histology. (**B**) Distribution of muscle fiber diameters. (**C**) Median muscle fiber diameter. (**D**) Total number of muscle fibers. (**E**) Percentage of fibers with centralized nuclei. (**F**) Representative H&E-stained cross-sections of diaphragm and intercostal muscles. (**G**) Immunofluorescence images of diaphragm cross-sections from 8-week-old mice stained for αLNNd or αLNNdΔG2 using antibodies against the LN domain of laminin-α1. Mice were injected intravenously at P1 with the indicated constructs (1.5×10^{14} vg/kg per construct). Data are presented as mean ± SEM. *$P < 0.05$, **$P < 0.01$, ***$P < 0.001$ by one-way ANOVA with Dunnett's post hoc test, comparing each group to untreated *dy^{W}/dy^{W}* mice. Scale bars: 500 µm (A), 100 µm (F, G). *n* = 3-10 mice per group.

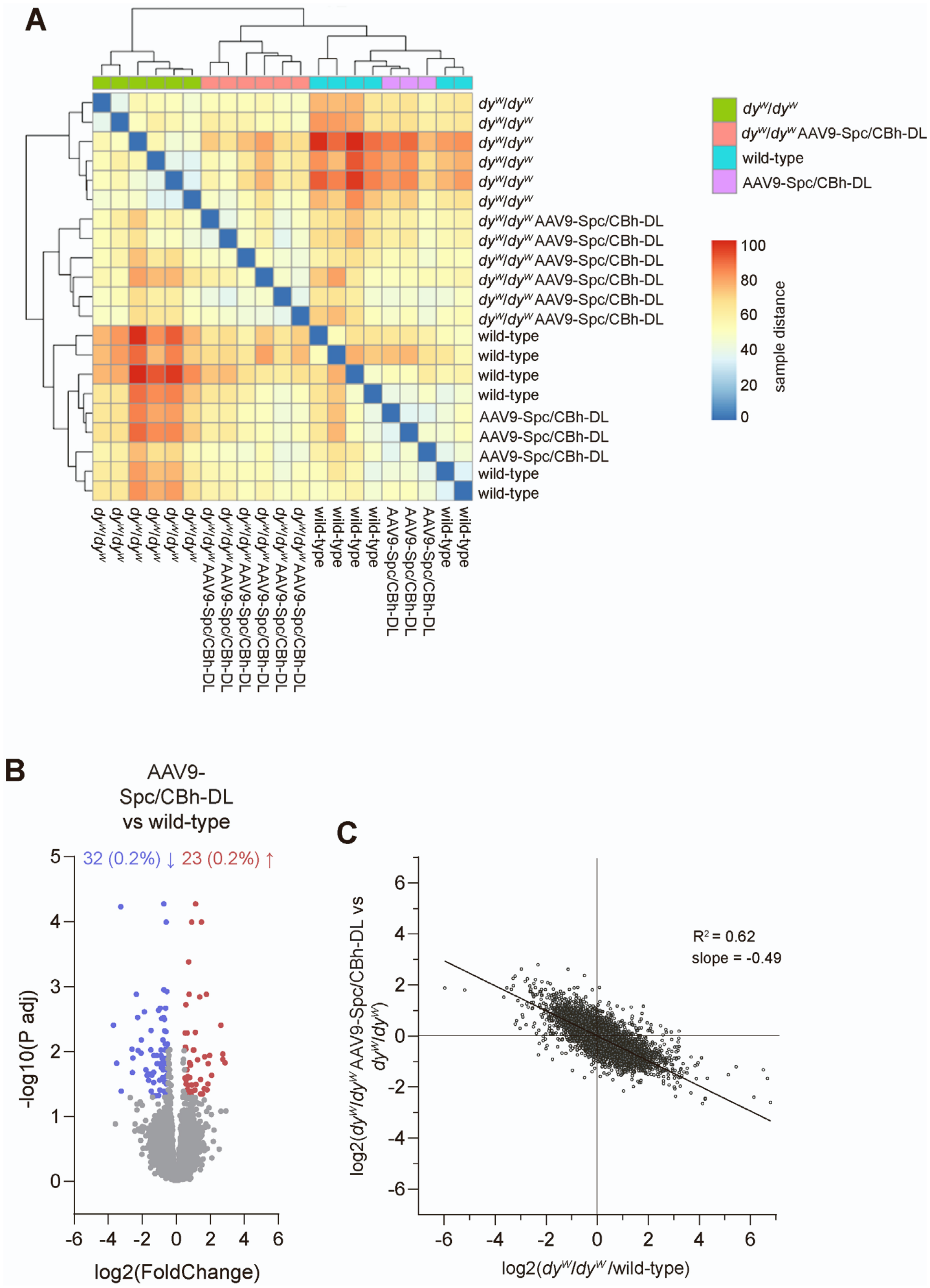

Figure S8. RNA-seq analysis of transcriptional changes by AAV9-mediated expression of mag and αLNNdΔG2 in *dy*^{W}/*dy*^{W} mice and wild-type controls.

(**A-E**) RNA-seq analysis of TA muscle from 8-week-old mice: non-injected wild-type and *dy*^{W}/*dy*^{W} mice, and wild-type and *dy*^{W}/*dy*^{W} mice injected intravenously at P1 with AAV9-Spc/CBh-DL (1×10^{14} vg/kg per construct). (**A**) Sample correlation blot of RNA-seq data. (**B**) Volcano plot showing the 14,905 transcripts detected in wild-type mice injected with AAV9-Spc/CBh-DL versus non-injected wild-type mice. Differentially expressed genes ($\log_2$ fold change > ±0.5; adjusted $p < 0.05$) are shown in red (upregulated) or blue (downregulated). Numbers above indicate the total number and percentage of upregulated (red) or downregulated (blue) transcripts. (**C**) Pairwise comparison of gene expression changes: Fold change in *dy*^{W}/*dy*^{W} versus wild-type mice plotted against fold change in *dy*^{W}/*dy*^{W} AAV9-Spc/CBh-DL versus untreated *dy*^{W}/*dy*^{W} mice. *n* = 3-6 mice per group.

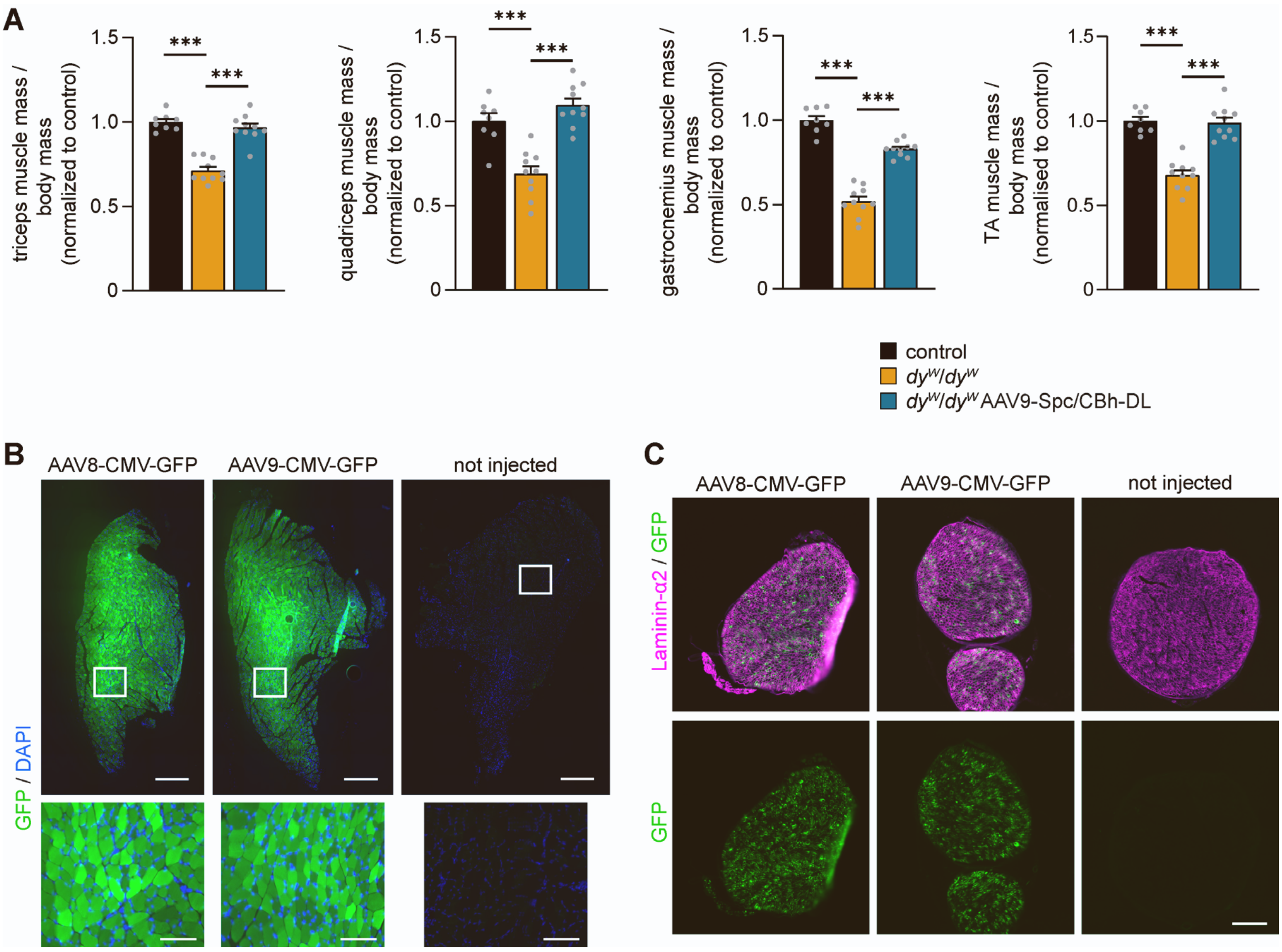

Figure S9. Comparison of AAV9 and AAV8 transduction efficiency in muscle and peripheral nerve.
(**A**) Muscle mass normalized to body mass of 8-week-old wild-type, *dy*^{W}/*dy*^{W} or *dy*^{W}/*dy*^{W} mice intravenously injected at postnatal day 1 (P1) with AAV9-Spc/CBh-DL (1×10^{14} vg/kg per construct). (**B-C**) C57BL/6 mice were injected intravenously at P1 with CMV-GFP packaged into AAV9 or AAV8 (1×10^{14} vg/kg). Tissue was analyzed at 4 weeks of age. (**B**) Immunofluorescence images of TA muscle cross-sections stained for GFP. (**C**) Immunofluorescence images of sciatic nerve cross-sections stained for laminin-α2 and GFP. Data are presented as mean ± SEM. ****P* < 0.001 by one-way ANOVA with Dunnett's post hoc test, comparing each group to untreated *dy*^{W}/*dy*^{W} mice. Scale bars: 500 µm (B, top) and 100 µm (B, bottom and C).

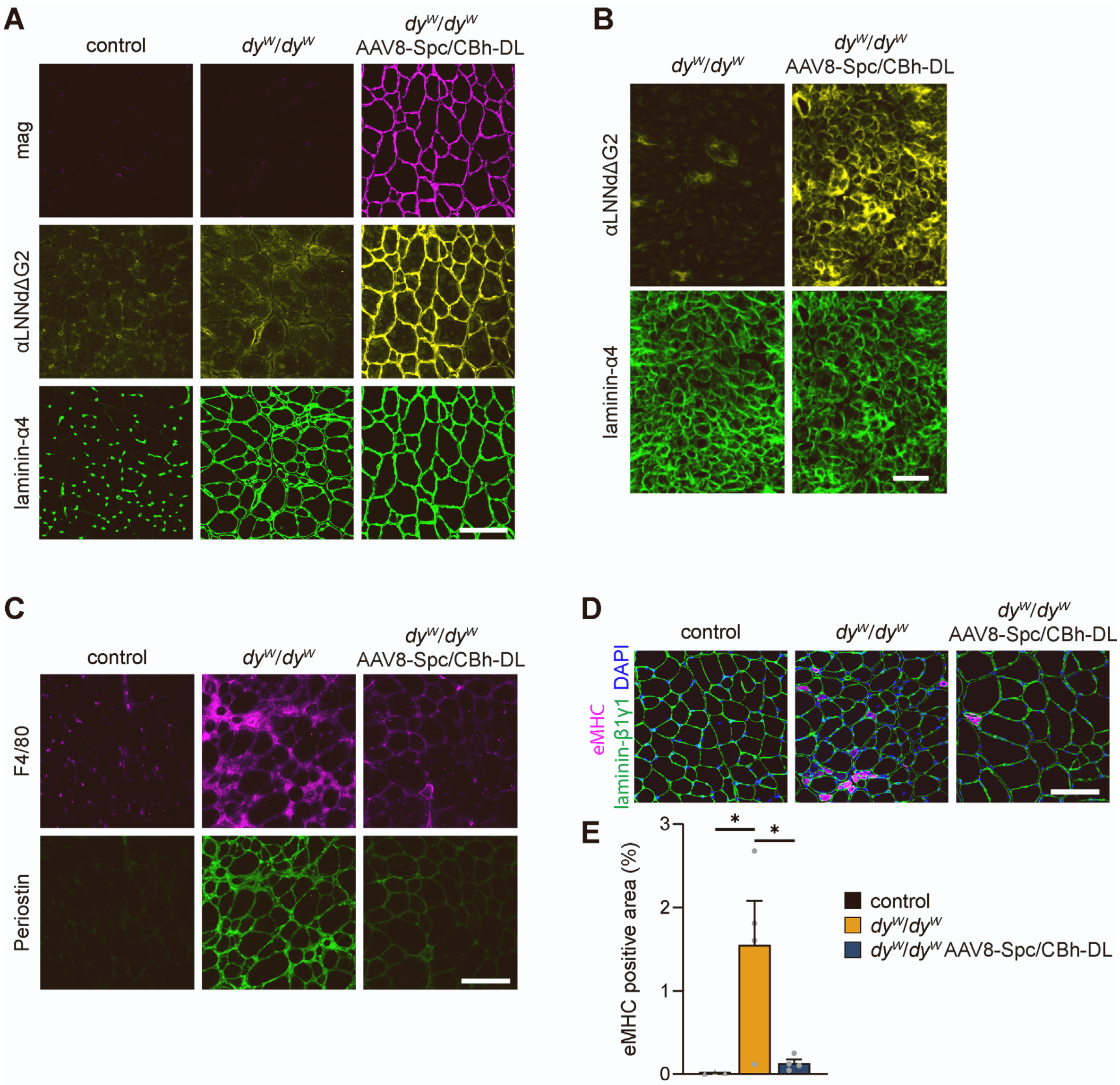

Figure S10. Improvements in degeneration/regeneration, inflammation and fibrosis by AAV8-mediated co-expression of mag in muscle and αLNNdΔG2 in muscle and nerve.
(**A-D**) Representative immunofluorescence images of TA cross-sections (A, C and D) or sciatic nerve (B) from 8-week-old heathy control, *dy*^{W}/*dy*^{W} or *dy*^{W}/*dy*^{W} mice injected at P1 with AAV8-Spc/CBh-DL (1×10^{14} vg/kg per construct). Cross-sections were stained for mag, αLNNdΔG2 and laminin-α4 (**A-B**); F4/80 (macrophage marker), Periostin (fibrosis marker) (**C**); eMHC and laminins (β1γ1-chains) (**D**). (**E**) Quantification of eMHC-positive area. Data are presented as mean ± SEM. *$P < 0.05$ by one-way ANOVA with Dunnett's post hoc test, comparing each group to untreated *dy*^{W}/*dy*^{W} mice. Scale bars: 100 μm (A, C, D) and 20 μm (B). *n* = 3-4 mice per group.

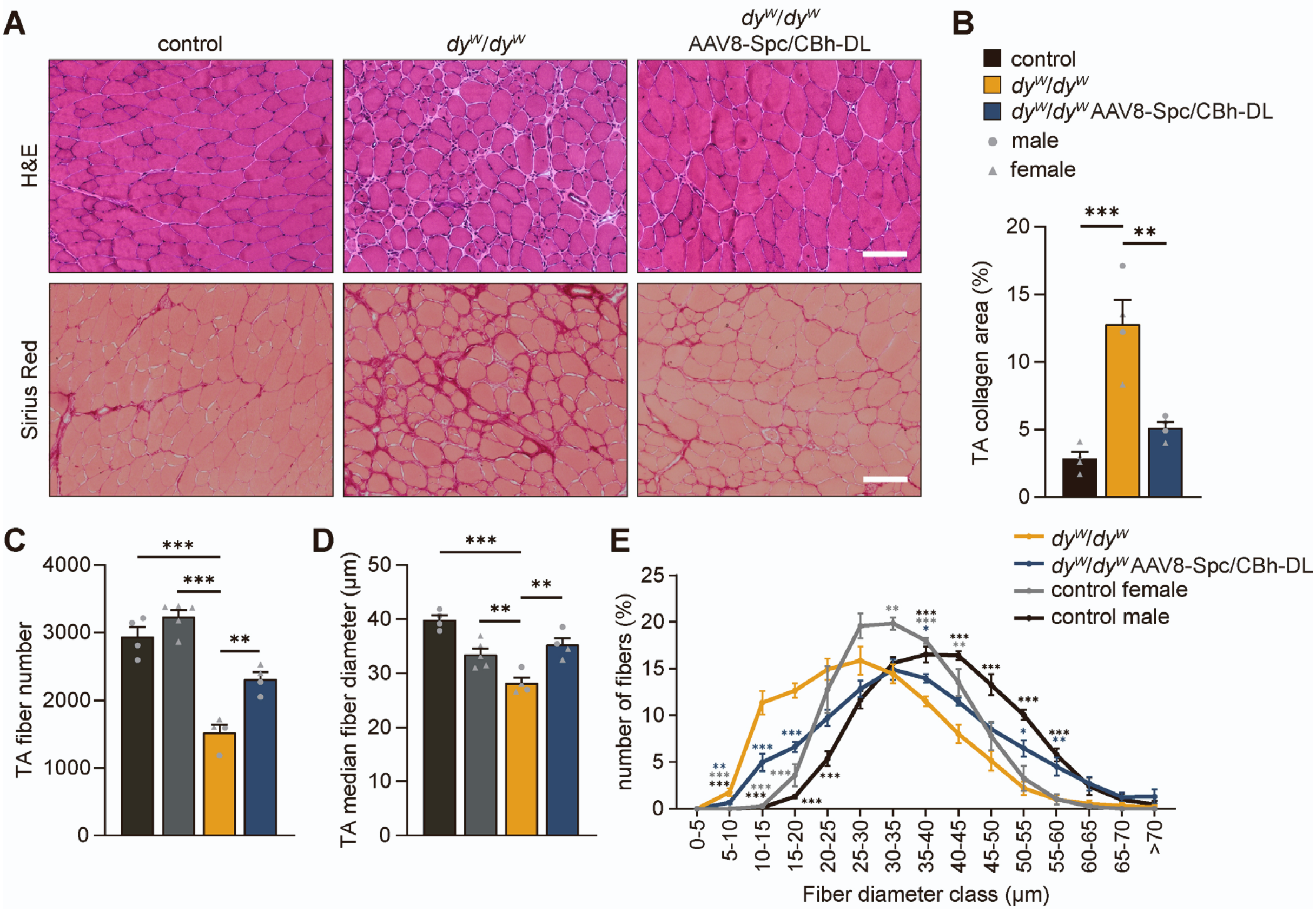

Figure S11. Improvements of TA muscle histology by AAV8-mediated co-expression of mag in muscle and αLNNdΔG2 in muscle and nerve.
(**A-E**) Analysis of TA muscle histology from 8-week-old wild-type, *dy*^{W}/*dy*^{W} or *dy*^{W}/*dy*^{W} mice injected at P1 with AAV8-Spc/CBh-DL (1×10^{14} vg/kg per construct). (**A**) Representative images of H&E- and Sirius Red-stained TA muscle cross-sections. (**B**) Quantification of muscle fibrosis based on Sirius Red-positive area in TA cross-sections. (**C**) Total muscle fiber number. (**D**) Median muscle fiber diameters. (**E**) Distribution of muscle fiber diameters. Data are presented as mean ± SEM. ***P* < 0.01, ***P* < 0.01, ****P* < 0.001 by one-way ANOVA with Dunnett's post hoc test, comparing each group to untreated *dy*^{W}/*dy*^{W} mice. Scale bar: 100 µm. *n* = 4 mice per group.

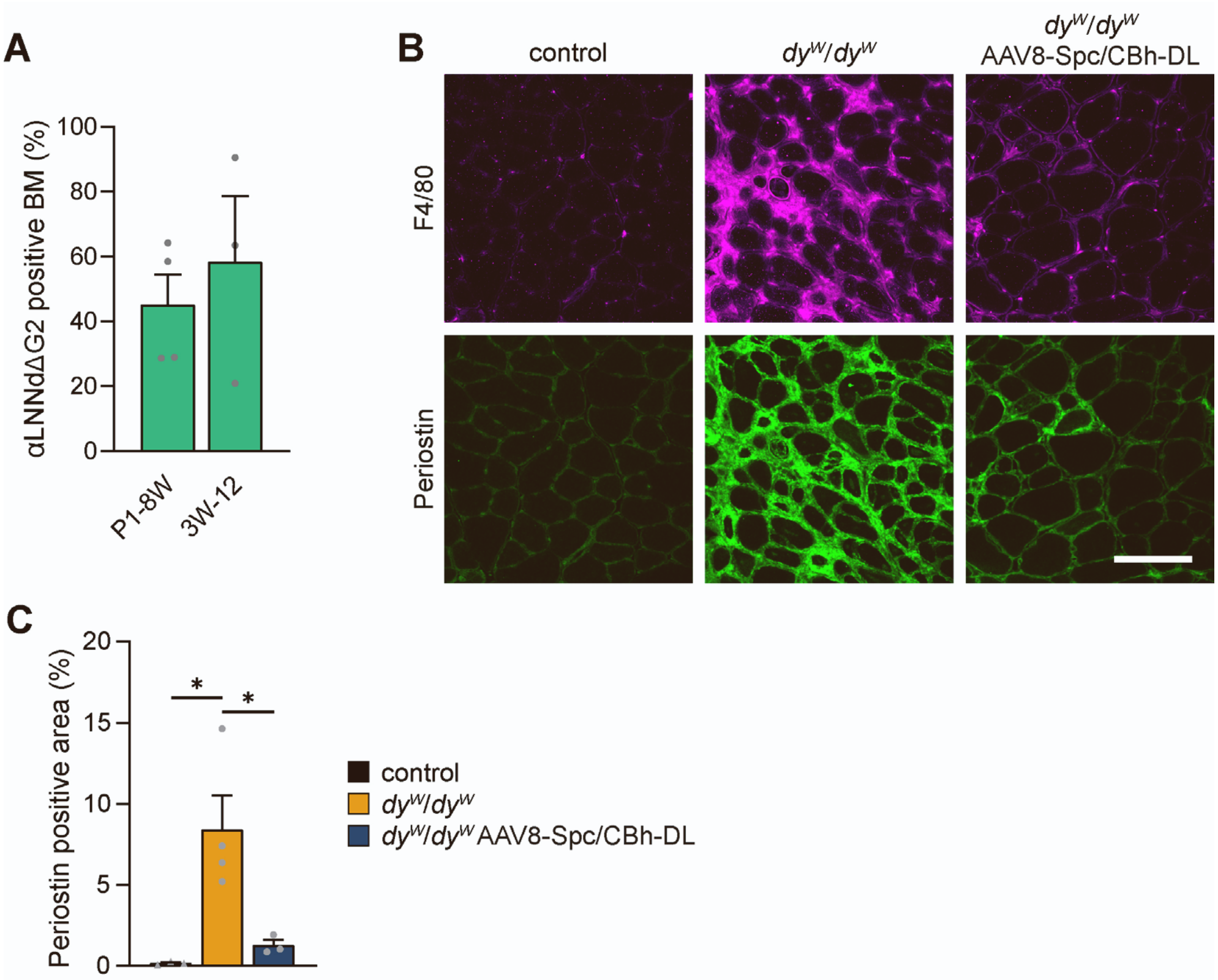

Figure S12. Linker protein basement membrane coverage in peripheral nerve and histological improvements with late treatment.
(**A**) Quantitative analysis of αLNNdΔG2 basement membrane (BM) coverage in sciatic nerve cross-sections based on immunostaining (representative images shown in Figure S10B and Figure 8C). BM coverage was measured as the percentage of laminin-α4–positive endoneurial BM that also contained αLNNdΔG2 following either P1 injection with 1×10^{14} vg/kg and analysis at 8 weeks of age, or treatment at 3 weeks of age with 5×10^{13} vg/kg AAV8-Spc/CBh-DL and analysis at 12 weeks of age. (**B**) Representative immunofluorescence images of TA muscle cross-sections from 12-week-old heathy control, *dy*^{W}/*dy*^{W} or *dy*^{W}/*dy*^{W} mice injected at the age of 3 weeks with AAV8-Spc/CBh-DL (5×10^{13} vg/kg per construct). Cross-sections were stained for F4/80 (macrophage marker) and Periostin (fibrosis marker) (**C**) Quantification of Periostin-positive area. Data are presented as mean ± SEM. $^{*}P < 0.05$ by one-way ANOVA with Dunnett's post hoc test, comparing each group to untreated *dy*^{W}/*dy*^{W} mice. Scale bar: 100 µm. n = 3-4 mice per group.

Video S1: Phenotype of 5-week-old *dy^W^/dy^W^* mice treated with AAV9-Spc-m.DL

Representative video of a 5-week-old healthy wild-type, untreated dy^W/dy^W mouse and dy^W/dy^W mouse intravenously injected at P1 with AAV9-Spc-m.αLNNd and AAV9-Spc-m.mag (together referred to as AAV9-Spc-m.DL) at a dose of 1.5×10^{14} vg/kg per construct.

Video S2: Hindlimb paralysis in 8-month-old dy^W/dy^W mouse treated with AAV9-Spc-DL

Representative video of an 8-month-old dy^W/dy^W mouse intravenously injected at P1 with AAV9-Spc-DL (1.5×10^{14} vg/kg per construct).

Video S3: No signs of peripheral neuropathy in 8-month-old dy^W/dy^W mouse transgenically expressing αLNNd under a ubiquitous promoter

Representative video of an 8-month-old dy^W/dy^W CAG-αLNNd transgenic mouse showing normal gait and no evidence of peripheral neuropathy.

Video S4: Peripheral neuropathy in 8-week-old dy^W/dy^W mouse transgenically expressing mag under a ubiquitous promoter

Representative video of 8-week-old dy^W/dy^W CAG-mag transgenic mouse, displaying signs of peripheral neuropathy.

Video S5: Close-to-normal phenotype in dy^W/dy^W mice treated with AAV9-Spc/CBh-DL

Representative video of 8-week-old wild-type, dy^W/dy^W and dy^W/dy^W mice injected intravenously at P1 with AAV9-Spc/CBh-DL (1×10^{14} vg/kg per construct).

Video S6: Improved neuromuscular phenotype by late treatment

Representative movie of 12-week-old untreated dy^W/dy^W mice and dy^W/dy^W mice injected intravenously at 3 weeks of age with AAV8-Spc/CBh-DL (5×10^{13} vg/kg per construct). Treated mice show improved motor performance.
